# Engaging with artificial intelligence in mammography screening: Swedish breast radiologists’ views on trust, information and expertise

**DOI:** 10.1177/20552076241287958

**Published:** 2024-10-07

**Authors:** Charlotte Högberg, Stefan Larsson, Kristina Lång

**Affiliations:** 1 Department of Technology and Society, 101103 Faculty of Engineering, Lund University, Lund, Sweden; 2 535389 Department of Translational Medicine, Diagnostic Radiology, Lund University, Lund, Sweden; 3 Unilabs Mammography Unit, Skane University Hospital, Malmö, Sweden

**Keywords:** Artificial intelligence, breast cancer, mass screening, radiologists, trust, transparency, information, literacy, expertise

## Abstract

**Objectives:**

Lack of trust and transparency is stressed as a challenge for clinical implementation of artificial intelligence (AI). In breast cancer screening, AI-supported reading shows promising results but more research is needed on how medical experts, which are facing the integration of AI into their work, reason about trust and information needs. From a sociotechnical information practice perspective, we add to this knowledge by a Swedish case study. This study aims to: (1) clarify Swedish breast radiologists’ views on trust, information and expertise pertaining to AI in mammography screening and (2) analytically address ideas about medical professionals’ critical engagement with AI and motivations for trust in AI.

**Method:**

An online survey was distributed to Swedish breast radiologists. Survey responses were analysed by descriptive statistical method, correlation analysis and qualitative content analysis. The results were used as foundation for analysing trust and information as parts of critical engagements with AI.

**Results:**

Of the Swedish breast radiologists (*n* = 105), 47 answered the survey (response rate = 44.8%). 53.2% (*n* = 25) of the respondents would to a high/somewhat high degree trust AI assessments. To a great extent, additional information would support the respondents’ trust evaluations. What type of critical engagement medical professionals are expected to perform on AI as decision support remains unclear.

**Conclusions:**

There is a demand for enhanced information, explainability and transparency of AI-supported mammography. Further discussion and agreement are needed considering what the desired goals for trust in AI should be and how it relates to medical professionals’ critical evaluation of AI-made claims in medical decision support.

## Introduction

The work of healthcare professionals is increasingly impacted by digital clinical decision support systems.^1^ However, using artificial intelligence (AI) to support or automate medical decisions comes with a multitude of concerns, including how to maintain and promote core principles of medical ethics and evidence-based clinical practice.^2^ Medical professionals’ trust in AI is moreover claimed to be an important missing component for successful responsible implementation of AI.^3^ Commonly, distrust in AI is argued to be fuelled by a lack of transparency, explainability and accountability.^4–9^

Within the field of radiology, AI is rapidly advancing, stressing the question of how medical professionals should engage with AI, and how to address aspects of clinical judgement, transparency, literacy and trust. One area targeted as especially suited for AI application is the reading of mammograms in breast cancer screening, which shows promising results in terms of reduced screen-reading workload and a potentially higher accuracy in cancer detection.^10^ This is as AI can be deployed to support the interpretation of the screening exam by deep learning based image analysis, able to provide a risk score (high versus low probability of malignancy) that can be used as triage and region of interest in the image. Exactly how AI can be integrated, and what the radiologist uses, in the screen reading process varies both depending on system capabilities and local preferences of the radiology department.^11^ By population-based mammography screening, breast cancer can be detected earlier, reducing the mortality and morbidity of the disease.^12^ However, it also entails potential harms of unnecessary call-backs and overdiagnosis of indolent cancers.^13,14^ The social, ethical and legal consequences of integrating AI in mammography screening are in need of more exploration,^11,15–17^ and for radiology in general, these are argued to be paramount hurdles for clinical implementation of AI.^18–21^

Recent research stress the need for transparency of AI in radiology to justify diagnosis, treatment and outcome prediction.^22^ Moreover, clinicians’ trust is emphasized as needed for AI to work as a support in radiology.^23^ Breast radiologists’ trust in AI has also been identified as a main challenge to overcome.^16,24^ However, there is little common understanding of the meaning and motives underpinning calls for trust in medical AI and what is required to achieve it.^25^ One instrument stressed as able to improve trust in AI in the clinic^26^ and medical imaging^27^ is increased transparency. Specifically, explainability is claimed to be necessary for assessing trustworthiness of AI applications in sociotechnical systems of high-stake decision-making.^28^ Judging by stakeholder perspectives, transparency has been identified as a main condition for trust in AI in radiology^23^ and while the communication of AI systems has been stressed as a main component in breast cancer detection, the studies exploring factors impacting adoption and usability from the clinicians’ perspective are still relatively scarce.^29^ This calls for more empirical research on trust and explainability in clinician-facing AI, as argued by Asan et al.: ‘in what capacity can AI assist clinicians and will clinicians be able to use and assess the reliability of an AI system?’.^26^ Considering the current development of AI for breast cancer detection, breast radiologists are likely to be amongst the first to navigate how to incorporate AI decision support in their clinical judgements.^10^ Moreover, the specific context of mammography screening entails applying AI in high-stake decisions on a large population.

By drawing from information studies and science and technology studies, we acknowledge AI in mammography screening not only as a technical or medical concern, but as a sociotechnical and sociomaterial development.^30^ We thus argue that there is a need for more empirical knowledge of different actors’ views regarding AI and medical professionals’ *critical engagements with AI*. This can be understood as in line with what Lebovitz et al. call engaged augmentation,^31^ referring to practices of interrogating AI claims, in relation to one's own expertise and judgement, in contrast to disregarding or accepting AI knowledge claims without any further consideration.

Guided by the identified need for knowledge, we conducted a survey on Swedish breast radiologists’ views on AI in mammography screening. The results that have previously been published show that a majority of the radiologists are positive towards integrating AI,^11^ yet do they trust AI assessments and what kind of information can support evaluations of trust? This study aims to:

1. clarify Swedish breast radiologists’ views on trust, information and expertise pertaining to AI in mammography screening, and
2. analytically address ideas about medical professionals’ critical engagement with AI and motivations for trust in medical AI.

While the first aim relates to the empirical ambition of this study, the second aim requires a more comprehensive theoretical discussion. With these aims, we strive to expand the knowledge on trust, transparency and informational aspects of integrating AI into breast cancer screening and medical decision-making, and how it is, or can be, transforming digital healthcare work and professional expertise.

## Background and theoretical framework

In this section, we situate trust and information within the context of medical AI and breast cancer screening and introduce the theoretical framework of this study. From a *sociotechnical* perspective, we expand on the concepts of trust and AI, the human-in-the-loop, AI literacy and information practices, as well as explanations and trust configurations, in relation to the field of radiology.

### AI and trust relations

The concept of trust is multi-layered, relational and situational. In this study, we build on the characteristics provided by Gille et al.:

‘Trust’ is relational, highly complex and involves at least two actors: one actor trusts the other actor to do, or not to do, an activity. This relationship is influenced by diverse framing factors — culture, belief systems, context, to name a few — and by the traits of the individual actors in the relationship. Therefore, trust is highly situational and difficult to develop as a ‘general concept’.^25^

Trust in AI for implementation in healthcare has been conceptualized in different ways.^32^ Whether or not it is possible to trust AI, as a non-human entity for medical use, or if trust requires human reciprocity, is subject for debate. Some argue that AI at best can be relied upon if an acceptable level of performance has been confirmed.^33,34^ To add to the complexity, there is a difference between well-informed trust and blind (potentially unwarranted) trust. Moreover, trust is more than an individual endeavour or issue for human–computer interaction, also involving multiple human and non-human agents and organizations, structures and processes.^35^

Within mammography screening, trust is an important matter of concern in relation to both the vulnerability of the screening participant as a care-receiver in a high-stake situation, and the vulnerability of the radiologist as the one liable for medical decision-making. With the integration of AI as a screen-reading support, the radiologist could be assessing AI findings along with their own interpretations of the screening exam, depending on the chosen layout of the workflow. In the same manner as with current practice of double reading, where two human radiologists assess each screening exam, the assessment made by the system (presented as risk score and/or markers in the image) could be either fully or partly hidden to the radiologist in the screen reading interface and hanging protocol. This corresponds with the way that the first reader's decision is concealed to the second reader in independent blinded double reading. However, the AI output can also be visible to the radiologist as a way to support the medical decision-making and augment the radiologists’ capabilities. This depend on the workflow strategies for AI implementation.^24^ While AI is commonly expected to be integrated as replacement of a second reader, some breast radiologists would prefer that AI is integrated in the workflow as an additional support to human double reading.^11^

Previous research however suggests that a lack of trust and perceived opacity of AI could result in radiologists not making use of the diagnostic findings provided by AI systems.^31^ If the system's outcome is disregarded by default due to a general lack of trust, it can represent a missed opportunity to take advantage of the AI-technologies capabilities and potential augmentation of clinical work,^31^ and a potentially improved patient outcome. In addition, it would imply a waste of resources to implement systems that are not used or perceived as useful. In their study on stakeholders’ perspectives on prerequisites and obstacles for achieving trust in AI in radiology, Bergquist et al.^23^ furthermore identify transparency as one of the main conditions for trust. When Norwegian breast radiologists were asked to list potential challenges of introducing AI in mammography screening, the lack of trust in the AI system was the most frequent answer.^24^ Overall, how trust in AI is used, and what the concept is expected to carry, is also a discursive matter of valuation, by asking when the algorithmic modelling should be considered good enough to be trustworthy, and what a good distribution of agency between humans and technology is.^36^

### Sociotechnical meaning-making and information landscapes

The importance of including radiologists in the development of AI has been stressed, but also that time, resources, expertise and trust are vital aspects to realize the radiologists-in-the-loop.^37^ Clinicians’ involvement in validating AI can also help to build trust towards technology.^38^ Moreover, the views of experts and their epistemic cultures^39^ impact the implementations of new technologies. This illustrates how introducing AI is a sociotechnical and sociomaterial matter, combining traits of technological artefacts, work and organizations, and components of language, interaction and different practices forming this arrangement.^30^

AI literacy is furthermore intertwined with other types of literacies.^40^ With regard to transparency, information literacy is key in the process of drawing from sites of knowledge that can be social, epistemic, physical, analogue or digital, and part of a workplace, education or community. Moreover, literacy can require knowledge of particular technologies and visual presentations.^41^ In our strife to make sense of information, we increasingly have to make sense of algorithms, and how they are part of information, data and knowledge infrastructures.^42–44^ This is also the case for healthcare workers depending on, or working in tandem with, algorithmic systems. Yet, for AI transparency to facilitate accountability a critical audience needs to be able to review information^45^ within a wider landscape of information and trust relations and be able to assess sources.^46^ Infrastructural meaning-making could work to empower users.^42^ That is, to get to know and reason about the infrastructure behind AI development and system certification, and thereby providing an increased understanding, literacy and competence.

However, medical technologies are not to be regarded as objects that are passively registering facts and supporting knowledge tasks, but as entities that are intervening in the situations in which they are put to use, as argued by Mol.^47^ AI findings need to be considered in relation to pattern recognition training of radiologists,^48^ as they have to work together towards a clinical judgement in screen reading that is not blinded to the AI assessment, and work in tandem in blinded screen reading, while replacing the radiologist's gaze in full automation.^31,49^ AI should thus be considered in relation to the epistemologies of the eye,^50^ in terms of how knowledge is produced by the act of seeing and its cognitive and sociomaterial underpinnings.^48^ Yet, medical imaging is always technologically mediated and part of organizational structures and practices. Dealing with multiple sources of information and knowledge is also not a new process in medical decision-making, but a difference is possibly the claimed abilities of AI as a disease detecting, and diagnosis setting, partner.

### Information, explanations and trust configurations in medical AI

The deficient transparency of AI is argued to be due to intentional secrecy, a lack of literacy, and black-boxed technology, contributing to a cognitive mismatch caused by the large size of computational systems and the complexity of models.^51,52^ Moreover, AI transparency is a concept of multiple understandings and often vaguely defined.^53,54^ It is generally approached as a binary – either opaque or transparent – and static state, whilst there is a need to acknowledge AI transparency as a relational, non-binary, context-bound process.^55,56^ As such, meaningful transparency of AI in healthcare, situated in information practices and practices of knowledge production, can be considered as needed due to ethical and legal concerns.^9^ Moreover, a lack of AI literacy has been identified as a hurdle for AI implementation.^18–20,57^

What information and explainability is provided in the use of an AI system could thus impact trust and conditions for responsible AI integration.^27,28^ The importance of appropriate transparency has been stressed by radiological societies^4^ and perceived opacity risks to result in dismissal of AI findings in the radiologists’ diagnostic assessment.^31^ Communication of AI in breast cancer screening (by information, visuals and explanations of classifications, performance, lesion segmentation) could furthermore result in a higher perceived accuracy and help clinicians to control results, presumably increasing the trust in AI classifications.^58^ To add saliency maps of area of importance for classifications is argued to be one way of enhancing explicability.^17^ Adapting the communicated assertiveness of AI findings to the experience of the clinician has also been suggested as a way to increase trust and reduce medical errors.^29^

There are many reasons for why explanations of AI outputs are considered as needed, especially so in healthcare and medicine. Yet, to provide explanations of AI outputs risk to also instil an over-reliance in AI. This is one motivation for building a calibration of trust.^59^ Cabitza et al. found that example-based post hoc reflexive explainable AI support for radiologists could offer a long-run mitigation of both over-reliance in AI and erosion of agency, knowledge and skills.^60^ A lack of information may however not be perceived as an issue until cases out of the expected, or severe failures, occur in the clinical setting, possibly requiring consultation and interpretation of different information sources. Making sense of a system output entails a renegotiation of trust, the normal and expected, as AI becomes incorporated into clinical decision-making processes.^61^

While managing uncertainty is not a new issue in clinical practice,^62,63^ it has been claimed that levels of uncertainty of machine learning outputs need to be better communicated. Clinicians’ trust in AI would potentially increase if there were more calibrated models of uncertainty of the given prediction, and set thresholds for when a system should abstain from providing a prediction due to too high of an uncertainty.^64^

## Material, method and setting

This study is an empirical case study, by survey-based data collection of the views of Swedish breast radiologists. By using the empirical results as a foundation, we develop an understanding of trust and information as parts of critical engagements with AI as clinical decision support.

### Setting and target population

The setting for this study is Sweden, one of the most digitalized countries in the European Union,^65^ also representing early adopters of technology and breast cancer screening, with burgeoning studies of AI in that field.^10,66,67^ These factors make the Swedish breast radiologists a particularly relevant group to study as a potentially indicative case of AI in the clinic. In Sweden, the healthcare system is primarily universal, tax-financed and state-controlled, with mostly regional public healthcare providers.^68^ Through the national population-based breast cancer screening program, all with female as their legal gender, between the ages of 40–74, are invited to mammography screening every 18 to 24 months. In line with European guidelines,^69^ each screening exam is read by two radiologists (double reading) to ensure a high sensitivity.

The target population for the study comprised of all Swedish breast radiologists. The majority of the target group are part the Swedish Society of Breast Imaging (SSBI), providing an approximated size by the number of members (*N* = 105). No additional inclusion or exclusion criteria were applied. To reach the breast radiologists, we distributed an online survey (by the use of Sunet Survey) to the members of the SSBI. The survey was open for responses during one month in late 2021.

### Survey questionnaire and data collection

Based on the relevance for the social, ethical, and legal aspects of AI use, 45 questions were included in the questionnaire (Appendix A in the supplemental materials). Out of these, a subset concerned the themes of trust, literacy and information, forming the results presented in this article (for other results deriving from the survey and full questionnaire, see Högberg et al.^11^). The respondents were not given a definition of the concept of trust at the point of answering the survey, leaving the interpretation of the concept open. The response options were of Likert-scale, representing degrees (to a low degree, to a somewhat low degree, uncertain, to a somewhat high degree, to a high degree), except for the background questions that had categorical response options and two questions with free-text response option only. In addition, the respondents were invited to provide free text comments to all questions.

In accordance with the Swedish Ethical Review Act (2003:406, Sections 3–5), the study did not require ethical approval since no sensitive personal data was collected. Informed consent was obtained by a standard procedure for anonymous online research surveys. Initially, respondents were presented with an information letter describing the scope of the research, the voluntary nature of participation, data processing, legal basis for data collection, respondent's rights and project researcher's contact details for more information. Subsequently, consent was obtained by click response (‘I agree’ or ‘I do not agree’), forwarding consenting respondents to answer the survey.

### Data analysis

Responses were quantitatively analysed using descriptive statistical analysis, and correlations analysis by Spearman's rho (with 95% confidence interval [CI] and *p* values <0.05 as statistically significant), using IBM SPSS Statistics for Mac (Version 28.0, IBM Corp.). The responses are presented as proportions, frequencies and percentages. Free text responses and comments were analysed by inductive approach and content analysis. Comments were used as triangulation to gain additional understanding of the viewpoints and engagements with AI focused in the survey questionnaire.

## Results

The survey was answered by 47 of the 105 members of the SSBI, giving a response rate of 44.8%. Of the respondents, 53.2% (*n* = 25) were females, 46.8% (*n* = 22) were male, and none identified as other gender. The age of the respondents was fairly high, with 66.5% (*n* = 31) being over 50 years old. The majority had extensive experience in breast imaging (70.2% > 11 years of experience), and 76.6% (*n* = 36) performed more than approximately 5000 screen readings per year. 53.2% (*n* = 25) of the respondents had experience of using AI in breast radiology, out of which 31.9% regarded it as little/somewhat little and 21.3% as somewhat large/large (for comprehensive respondent characteristics, see Högberg et al.^11^).

In the subsequent sections, the results are presented thematically in the following order: (a) trust, (b) information to support trust evaluation and (c) literacy and expertise.

### Trust in AI assessments

**Figure 1.**
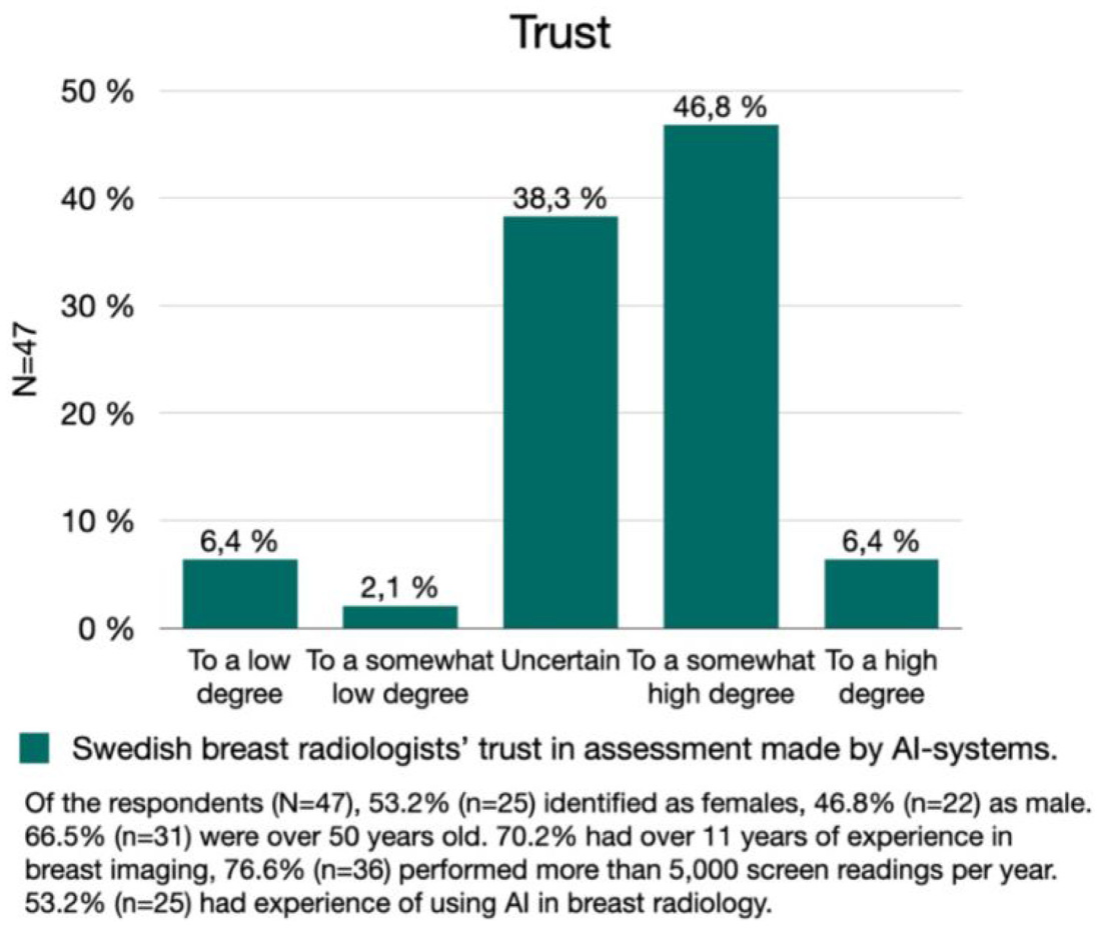
Trust. The Swedish breast radiologists' degree of trust in AI assessments of mammography screening exams.

Of the respondents, 53.2% (*n* = 25) would to a high/somewhat high degree trust AI assessments of screening exams. However, a considerable proportion, 38.3% (*n* = 18), were uncertain and 8.5% responded that they would to a low/somewhat low degree trust AI assessments (Figure 1).

Comments provided by the breast radiologists suggest that the level of trust could depend on under which conditions, and for what type of case, that AI is used:

My experiences are that you can to somewhat high degree trust AI-assessments on easy cases. But when the breast tissue is a little more difficult to assess, you can to a somewhat low degree trust the AI-assessment [P12].

Others indicated that the degree of trust assigned to AI assessments depends on which specific AI system is used. A respondent, stating to trust AI assessments to a somewhat high degree, added in a comment that: ‘…it is totally dependent on the choice of system. Only a few are good enough. Most need to improve’ [P18]. Moreover, some respondents stress that proven validity is a necessary condition for trust, as well as documentation proving that the system is performing as good as, or better, than a radiologist, and is thoroughly tested against the specific mammography equipment in use at the screening site.

In addition, the importance of first-hand experience was prevalent in several comments, expressed in statements such as ‘[I] need to see, with my own eyes, if it's up for the task …’ [P7], and ‘you need to test the system on your own material first’ [P4]. This presumes that trust in AI is something that is established as performed in practice. One aspect mentioned in the respondents’ comments was that the AI system needs to be able to compare with previous exams of the screening participant, before it can be trusted in screening. This points to the radiological practice of comparing images of current screening exams with previous ones, to decipher changes indicating malignancy. Several comments stress that a prerequisite for implementing an AI system for screen-reading in mammography screening is that it can be trusted.

If the breast radiologists’ assessments would be used to contribute to the AI system's continuous learning, by a feedback loop to re-train the AI model, a large proportion (63.8%, *n* = 30) considered that it would to a high/somewhat high degree increase their trust in the system. More than one out of four, 27.7% (*n* = 13), were uncertain as of whether it would have any impact on their trust.

**Figure 2.**
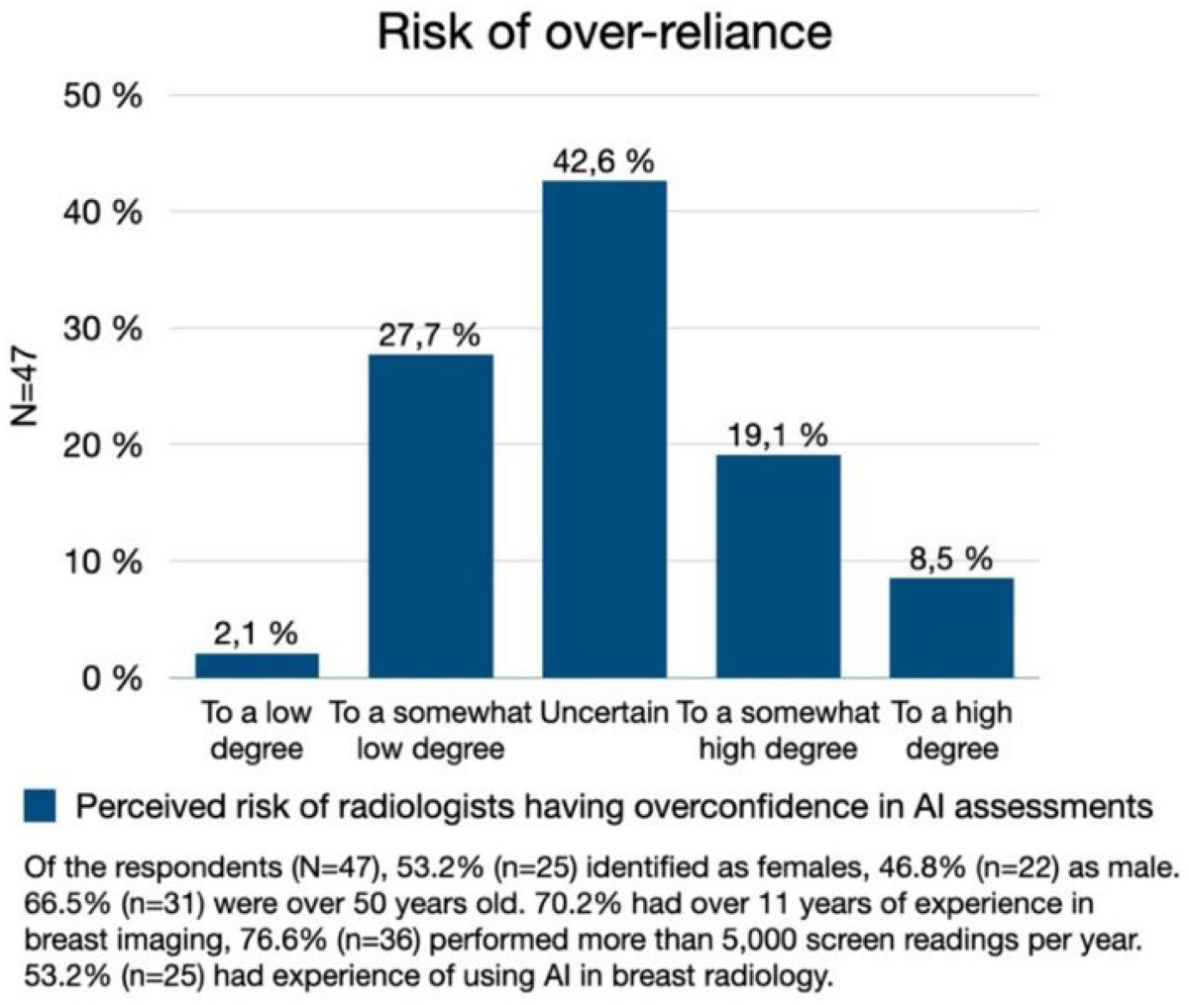
Over-reliance. The Swedish breast radiologists' perceived risk of radiologists becoming overconfident in AI assessments of mammography screening exams.

There is also a risk of overreliance in AI. When asked to what degree the respondents believe there is a risk of breast radiologists having overconfidence in assessment made by AI systems, 29.8% (*n* = 14) stated there to be a risk only to a low/somewhat low degree. Nearly as many considered there to be a risk to a somewhat high/high degree, while 42.6% (*n* = 20) were uncertain (Figure 2). Comments suggested that the respondents believe the risk of overconfidence in AI to be higher amongst inexperienced breast radiologists. However, others stressed that the AI assessments need to be used sensibly, and that it has to be established *how* AI systems can be trusted before integrating them into the screen reading process.

### Information to support the evaluation of trust

More than half (*n* = 16, 53.4%) of the respondents with previous experience of using AI in screening stated that they had to a low/somewhat low degree received the information needed to be able to review the accuracy of AI assessments ahead of or during the use of the system, or were uncertain as of whether they had received such information. If AI was to be used in screen reading, a majority (*n* = 35, 74.4%) of the breast radiologists stated that they would like to receive information regarding the system's development (e.g., about training data) to a high/somewhat high degree.

**Figure 3.**
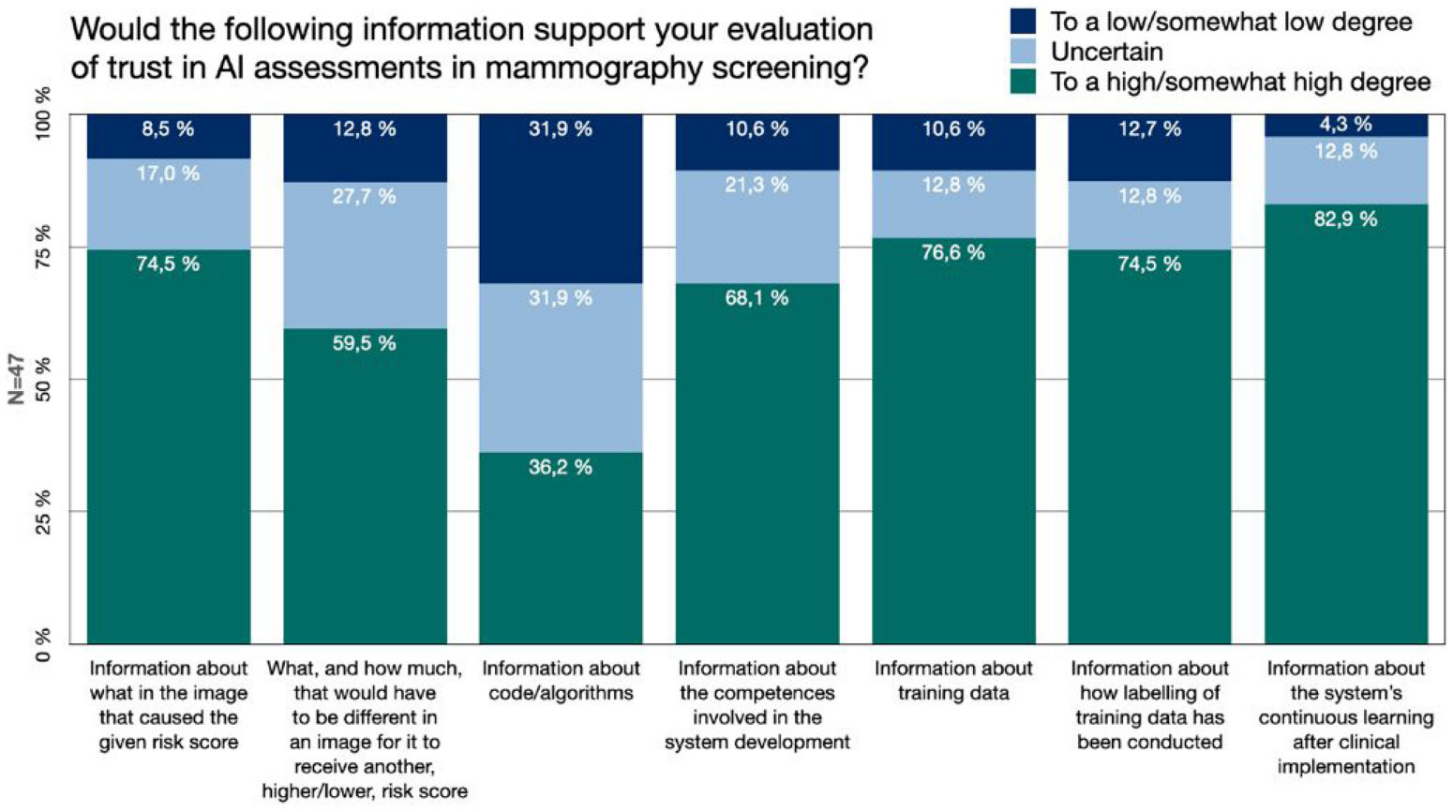
Information. The Swedish breast radiologists’ perceived value of different types of additional information about AI development, function and use, for the purpose of evaluating trust in AI assessments.

Accuracy levels are expected to be of value for the clinician in the use of medical AI, for example in the form of performance measures such as the sensitivity and specificity of the AI model. For the sake of gaining further knowledge about what would be meaningful transparency, and what information that would potentially be useful for the radiologists, we asked if certain types of additional information would be of support for the breast radiologists’ evaluation of whether, or to what extent, they should trust an AI system's assessment in mammography screening (Figure 3).

The responses showed that 74.5% (*n* = 35) of the Swedish breast radiologists would to a high/somewhat high degree be supported by information regarding what in the image caused the given risk score (Figure 3). This could potentially be visual information from image analysis, such saliency maps. 59.6% (*n* = 28) stated that it would to a high/somewhat high degree be of support for their trust evaluation to receive information about what, and how much, that would have to be different in the image for it to receive another (higher versus lower) risk score. This can be regarded as a potential contrastive, counter-factual, type of explanation.

Information about the AI developing process, its different steps and involved actors was highly valued for trust evaluation. More than 68.1% (*n* = 32) would be supported by information regarding what competences were involved in the development of the AI system (such as breast radiologists, computer scientists, statisticians, etc.), information about which training data was used (76.6%) and information regarding how labelling of the training data been conducted (e.g. how mammograms of cancer have been identified and annotated pre-training of the AI model) (74.5%) (Figure 3).

The value of information about the system's continuous learning after clinical implementation received the highest value. 83% (*n* = 39) of the radiologists considered that it would to a high/somewhat high degree support their trust assessment. Out of the information categories included in our questionnaire, the one perceived to be of least value to support the breast radiologists’ trust evaluation was information about the system's underlying code or algorithms. Still, more than a third (36.2%, *n* = 17) considered that it would support their trust assessment to a high/somewhat high degree (Figure 3).

In some comments, it was expressed that the radiologist should not need to consider any of these kinds of information after a system has received a certification for medical devices. As stated by one respondent: ‘I do have an interest in this [information], but if you don’t have that, you don’t need to delve into it, if the technology and the use of it has been approved’ [P27]. Moreover, regarding if more information about what in the image caused the given risk score would support the trust evaluation, one respondent made a comparison between AI and other medical technologies already in use to support the radiologists’ clinical judgement, without them necessarily having a large understanding of how the technology functions:

It is not really the radiologist's task, in the same sense as few radiologists understand the fundamental physics and algorithms used in, for example, the production of MR [Magnetic Resonance] images [P39].

On the other hand, other comments suggested that it is of great importance for the breast radiologists to have more insight, and to be furthered reassured of the underpinnings for the levels of sensitivity and specificity, in the use of AI. This suggests that there are diverse standpoints with regard to what knowledge and critical engagement with AI outputs that breast radiologists are supposed to perform.

### Perceived competence and AI literacy

Technical knowledge has been stressed as a factor impacting trust in AI. A majority (*n* = 31, 66%) of the Swedish breast radiologists considered that they would to a high/somewhat high degree have sufficient competence to evaluate whether the assessment made by an AI system is correct. However, more than a fourth (*n* = 13, 27.7%) were uncertain and 6.4% (*n* = 3) considered to have enough of competence for the task only to a low/somewhat low degree.

With regard to literacy, none of the Swedish breast radiologists considered their self-estimated technical literacy in everyday life or at work to be low. In everyday life, 42.6% (*n* = 20) stated it to be neither high nor low, nearly as many (*n* = 19, 40.4%) as somewhat high and 14.9% as (*n* = 7, 14.9%) high. The self-estimated technical literacy at work was slightly more frequently perceived as higher, with 36.2% (*n* = 17) estimating it to be neither high nor low, 44.7% (*n* = 21) estimating it to be somewhat high and 17.0% (*n* = 8) as high.

The self-estimated AI literacy was more frequently considered to be lower. 8.5% (*n* = 4) stated it to be low, 27.7% (*n* = 13) as somewhat low and 38.3% (*n* = 18) as neither high nor low. In comparison, 17% (*n* = 8) estimated their AI literacy to be somewhat high and 8.5% (*n* = 4) as high. Correspondingly, 21.3% (*n* = 10) of the breast radiologists stated their experience of using AI in their work to be large or somewhat large, while nearly a third (*n* = 15, 31.9%) had little/somewhat little experience, and 46.8% (*n* = 22) stated that they had no experience of using AI in their work.

With regard to any correlation between the respondents’ level of trust in AI and experience of using AI as well as their literacy, we did not find any clear relation. We could not establish a statistically significant correlation between the respondents’ degree of trust in AI assessments and their self-estimated AI literacy (Spearman's *r* = −0.111, 95% CI [−0.393, 0.191], *p* = 0.460), nor between trust and technical literacy at work (Spearman's *r* = −0.010, 95% CI [−0.286, 0.304], *p* = 0.946). A significant correlation between trust and experience of using AI could not be established either (Spearman's *r* = −0.048, 95% CI [−0.338, 0.251], *p* = 0.749).

## Discussion

### Relevance of trust

By investigating Swedish breast radiologists’ views on trust, information, literacy and expertise concerning AI in mammography screening, this study shows that a little more than half of the respondents would trust AI assessments of screening exams to a high/somewhat high degree. Yet, the number of respondents that were uncertain as of whether to trust the AI output was also notable. This corresponds with previous research stressing breast radiologists’ trust in AI interpretations as a main challenge for implementing AI in breast cancer screening.^16,24^ However, since the respondents were not provided with a definition of trust, they could have based their answers upon somewhat differing views of what it means to trust a technological output and a medical decision-support system.

Comments by the respondents suggest that the uncertainty of trust was not necessarily due to a general insecurity of whether AI assessments can be reliable, but that the level of trust, or whether to trust, depends on which particular system is used. Furthermore, that it depends on the specific conditions of the case upon which the AI screen reading is applied. Previous results show that Swedish breast radiologists believe there to be a higher risk of inferior AI performance on certain risk groups, or specific type of cases that are harder to assess.^11^ These aspects are likely impacting the level of trust, and the urge to evaluate trust, in AI assessments on a case-by-case basis. This can furthermore point to a practice of sound critical evaluation. Especially so, if one considers that clinical *reasoning* support is what AI systems are best to provide, rather than clear-cut deliverance of decisions.^70^ However, when systems are used not as support, but for stand-alone automation as a triage process only, sorting out the low-risk cases from being object to human radiologist screen-reading, trust must be presupposed in a rather different manner.

A majority of the respondents indicate that their trust in AI would increase if the radiologists’ assessment would contribute to a continuous learning of the algorithm. This do not only express a confidence in the accuracy of fellow radiologists’ judgement, but also point to a practice still rare in clinical implementation, presumably due to that AI systems that are adaptive to ongoing clinical assessments are not easily reconciled with regulatory frameworks on data sharing and certification.

### Information and trust relations

Our results emphasize that different types of information are perceived as highly valued for the breast radiologists’ evaluation of trust in AI assessments in mammography screening. Yet, not all information were as highly valued. Access to code is often a main focus for AI transparency policy discussions, despite transparency being a broad concept encompassing several other processes.^53^ Code and algorithms may be of great importance in audits and certification processes, but seem to be of somewhat less value for the clinician in the use of the system in the clinical setting. Instead, our results stress the value of local case-specific explainability, visual cues, information about system development and details about training data. This corresponds with the potential value of explainability measures in clinician-facing AI that has been proposed by previous studies, and the suggestion to incorporate an adaptability that can accommodate for variability between medical cases and reader experience to improve trust.^60,71^ The AI training and guidance frameworks, which have been requested by radiographers, could also work to support information types that is not easily incorporated into a system's interface and improve AI literacy.^72^

In general, our study shows that there is a large demand for information to support trust evaluations of AI assessments. This supports the notion that an increased understanding of the workings of AI-based clinical decision-support systems is important for clinicians’ calibration of trust.^73^ Moreover, it is in line with research suggesting the need for a holistic view on trust for implementation of healthcare AI.^32^ Still, the high degree of perceived support from the kinds of information included in this survey could also be an expression of a current anxiety about deep learning based AI as a newly introduced technology, and an uncertainty about the capabilities AI possesses as support in screen reading at the point of possible implementation. This is likely to change over time, as the institutional maturity grows with structural solutions rather than individual dependencies. It is worth considering if there is a continuous need, and an existing ability and possibility to review different types of information.

### Interplay of literacies, critical engagements and informed trust

Under the second aim of this study, we problematize what type of critical engagement with AI that the medical professionals are expected to perform, including what goals trust in medical AI should fulfil. The perception of not having had enough of information ahead of or when using AI systems, as shown in our study, resonates with previous findings amongst radiologists about not receiving sufficient information about AI in general.^74^ What information is needed for trust evaluation of AI assessments is entangled with what literacies and competences that it would require. Previously, a lack of knowledge about AI has been identified as a hurdle for AI implementation.^18–20,57^ We were not able to establish a statistically significant correlation between literacy and trust in AI amongst our respondents. There however seem to be various expectations of how critical and thorough the evaluation of AI findings should be, as performed by the radiologist.

Comments by the Swedish breast radiologists reveal that it appears unclear how to, or to what extent, critical engagement with AI finding is supposed to be executed. In this study, several were uncertain as of whether they had the competence needed to review AI findings, and previous research on radiologists has indicated a lack of training in AI.^74^ This could be signalling a need to clarify both what review is expected and what competence is needed when working with AI as clinical decision support. Moreover, there could be a need for other types of explainability methods in the interaction with AI systems, such as ways to more clearly express when there are high uncertainty in the assessment.^64^ Introducing intelligent agents with different assertiveness and design to customize explanations have also showed to impact clinicians’ trust, emphasizing the need for user involvement in the design process.^75^

There is also a need for clarification of what a certification of an AI system as a medical device implies, judging by the diverse statements that, on the one hand, information of the kinds included in the survey is not something that the radiologist needs to consider after a system's certification, and on the other hand, that it is important for the radiologist to have more insight. Furthermore, whether there is a difference in what level of caution AI outputs should be treated with, in comparison to previously introduced technologies. How much critical engagement is needed, or should be needed, after an AI decision support system has been certified for clinical use?

A higher level of AI literacy and training for work tasks is likely of great value for breast radiologists concerning current developments within the field. AI transparency and literacy is yet highly intertwined with ethical, legal and medical risks of using AI in mammography screening, such as if cancers are missed when AI is trusted or disregarded, or when radiologists are interpreting unclear AI findings. The multitude of uncertainties, regarding such matters as what information is to be considered, how or if to trust AI assessments, what review is necessary to perform, could represent hurdles to trust and implementation of AI. Furthermore, information work might be needed especially in the initial phases of AI implementation in the clinical setting. The need for continuous monitoring and professional development, considering the fast-moving field and increase in implementation, is also stressed by medical imaging societies.^76^ As becomes apparent from our study, trust in AI is also outsourced into medical certification and healthcare management. Overall, this speaks for the need of infrastructural meaning-making practices in AI trust evaluation.^42^

### Uncertainty and trust calibrations

Within the sociotechnical assemblages of clinical work, the AI findings become a new unit of information to consider, with uncertain grounds for credibility due to its novelty in the screening setting. In the same way as ‘alliterative errors’ potentially could be introduced if the second reader is influenced too heavily by the colleague's previous judgement in unblinded screen reading,^77^ an over-reliance or over-trust in AI can impact the radiologist's assessment, commonly referred to as ‘automation bias’. This can be the result of blind trust if no critical evaluation of AI findings is performed. The perceived risk of overconfidence, shown in our study, points to diverse views and a high number of uncertain respondents, even though comments suggest the risk is perceived as prevalent not with regard to the respondent's own judgement, but that of less experienced radiologists. Contestations of expertise and frictions in relation to technology have been the case also in previous technological advancements in radiology and clinical work, pitting technologists against medical professionals regarding what expertise is relevant and how to make sense of new diagnostic signs.^78,79^

The value assigned to algorithms and algorithmic outputs in clinical decision-making needs more exploration to better navigate the distribution of agency between humans and technology, as in studies of the biosciences.^36^ Furthermore, this relates to when technology should be considered trustworthy, and if we are willing to accept a technology that is considered trustworthy only in easy cases. If so, how do we establish in which cases the AI assessment cannot be trusted, and human judgement to be needed? And what practices and thresholds should be used to single out those cases at the point of medical screening? A blind trust in medical AI could be dangerous by the loss of valuable critical intervention from humans, whilst mistrust would render the use of AI support systems unjustified or result in non-use. These questions boil down to what level of trust and what type of trust mediation is desired for medical AI. What the appropriate goal for trust in medical AI should be needs to be agreed upon amongst the different stakeholders involved with implementation of AI in healthcare at large.

### Strengths and limitations

A limitation of this study is the potential lack of generalizability that the case and setting of the Swedish context of mammography screening may represent. Yet, there are clear motivations for using it as a potentially indicative case to gain important knowledge about different sociotechnical perspectives on clinical AI implementation, in terms of Sweden being a forerunner of digitalization, breast cancer screening and clinical AI trials.

The number of respondents is also a limitation of this study, even though the response rate can be considered sufficient in relation to similar studies^74^ and meta reviews (showing an average response rate of 44.1% for online surveys in published research).^80^ The Swedish breast radiologists is a rather small group of individuals, making it a limited target group. However, the respondents had long experience of working in breast imaging, and half of the breast radiologists had experience of using AI in radiology. It is also of importance to acknowledge that bias could be introduced in the results, by a likely inclination amongst individuals with strong opinions about AI integrations to participate in a survey like this. Consequently, this could result in both positive and negative bias, with regard to trust in AI.

## Conclusions

More than a half of the Swedish breast radiologists would trust AI assessments of mammography screening exams to a high/somewhat high degree. Still, the number of respondents uncertain as of whether to trust AI assessments was also notable. The respondents referred to differences in performance between systems, and between hard versus easy cases for AI to review, as impacting their trust. To a rather large extent the respondents also stated that additional information and explainability, such as information about AI-system development and detailed image-based explanation of what in the image caused the given risk score, would support their evaluation of trust in AI assessments. Information about code and algorithms was considered of less support for the breast radiologists in their trust evaluation. In terms of expertise, most of the Swedish breast radiologists considered themselves to have competence enough to review AI assessments of screening exams, but more than a fourth of the respondents were uncertain as of whether they had the competence needed.

This study shows that there is a need to clarify if, and how, medical professionals are expected to critically engage with AI assessments. Especially so, in relation to certification of medical devices, and what critical review and information work that is still needed post-certification to remain liable for medical decision-making with AI as decision support. What engagement is required also impacts how to reason about, and establish demands for, explainability of clinician-facing AI systems for high stakes decisions. In addition, what literacies and competences are needed for breast radiologists and other medical professionals working with AI in diagnostic assemblages depend on what is expected of them as humans-in-the-loop. Altogether, this study shows the need for sufficient information and explainability of AI aligned with the work of radiologists, especially in the implementation phases. Moreover, it stresses the importance of continuous information work and professional training to enable radiologists’ active engagement with AI as it is rapidly evolving. The goals for, or the role of, trust in medical AI need to be considered with a greater complexity in mind, paying more attention to enhanced trust calibration and what information and explainability that can support it. Overall, the objectives for trust in medical AI need further discussion and agreement amongst the relevant stakeholders. If aiming to live up to ideals of trustworthy AI, the significance of context-specific dimensions of trust and information needs cannot be overlooked.

## Supplemental Material

sj-docx-1-dhj-10.1177_20552076241287958 - Supplemental material for Engaging with artificial intelligence in mammography screening: Swedish breast radiologists’ views on trust, information and expertise Supplemental material, sj-docx-1-dhj-10.1177_20552076241287958 for Engaging with artificial intelligence in mammography screening: Swedish breast radiologists’ views on trust, information and expertise by Charlotte Högberg, Stefan Larsson and Kristina Lång in DIGITAL HEALTH
