# Supplementary material for: Engaging with artificial intelligence in mammography screening: Swedish breast radiologists’ views on trust, information and expertise
Source: Digit Health. 2024 Oct 7;10:20552076241287958. doi: 10.1177/20552076241287958 (PMC11459539; doi:10.1177/20552076241287958)
Supplement: sj-docx-1-dhj-10.1177_20552076241287958 - Supplemental material for Engaging with artificial intelligence in mammography screening: Swedish breast radiologists’ views on trust, information and expertise [file sj-docx-1-dhj-10.1177_20552076241287958.docx]

**Enkät: Bröstradiologers syn på användning av artificiell intelligens i mammografiscreening**


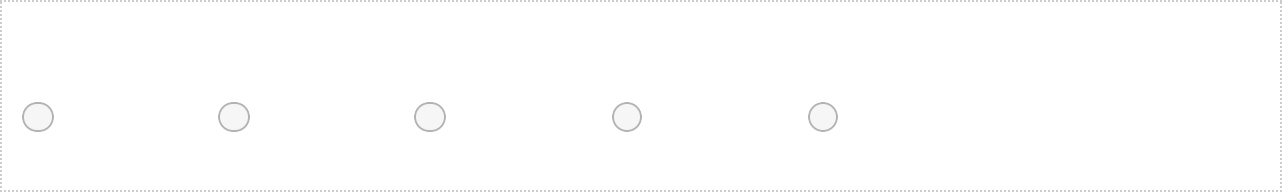


1. Ålder

Under 31 år

31-40 år

41-50 år

51-60 år

Över 60 år

# 


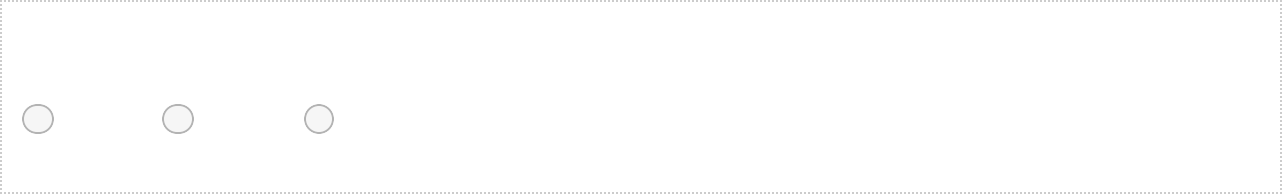


2. Kön

Kvinna

Man

Annat




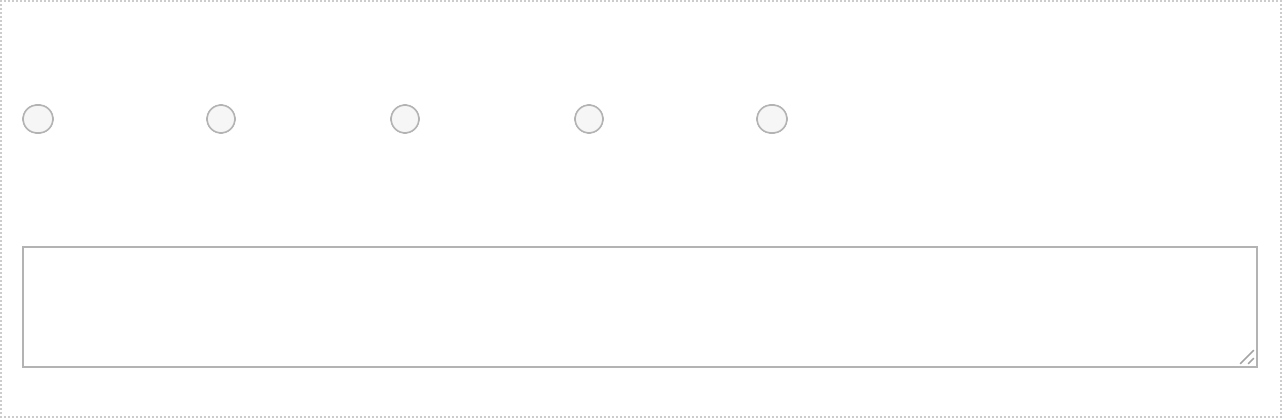


3. Hur lång erfarenhet har du av bröstradiologi?

Under 5 år

5-10 år

11-20 år

21-30 år

Över 30 år

Kommentar

# 


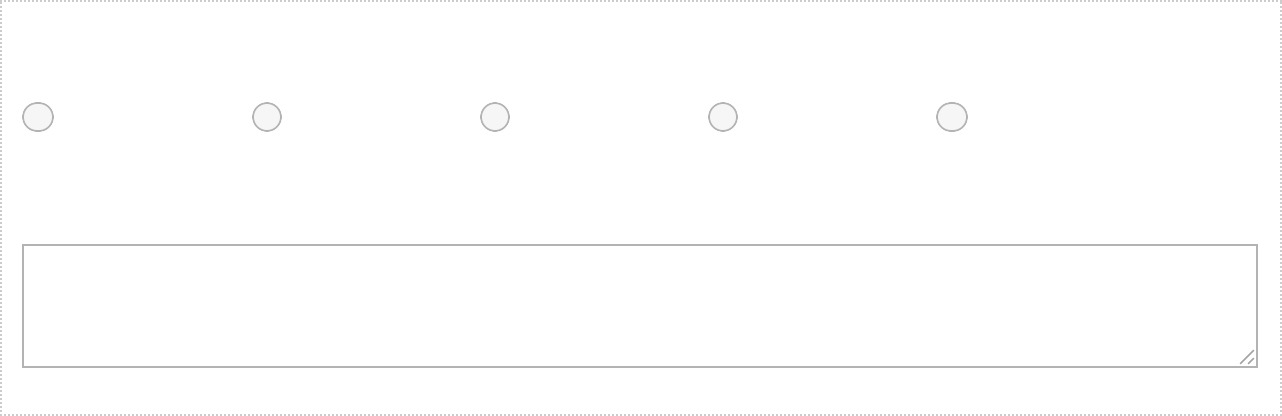


4. Hur många mammografiscreeningundersökningar granskar du uppskattningsvis per år?

Inga

Färre än 2 000

2 000 - 5 000

5 000 - 10 000

Fler än 10 000

Kommentar

# 


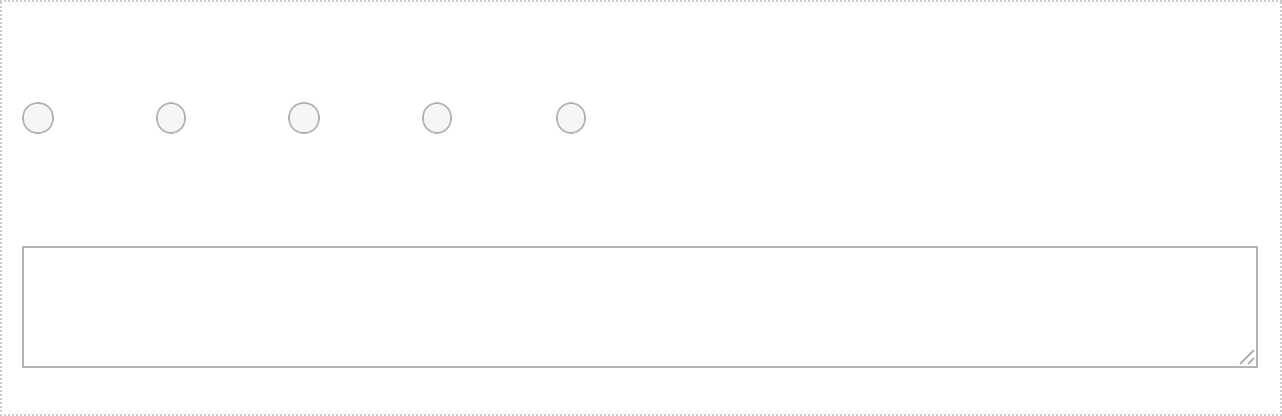


5. Upplever du att det är svårt att hinna granska screeningundersökningarna?

Aldrig

Sällan

Ibland

Ofta

Alltid

Kommentar


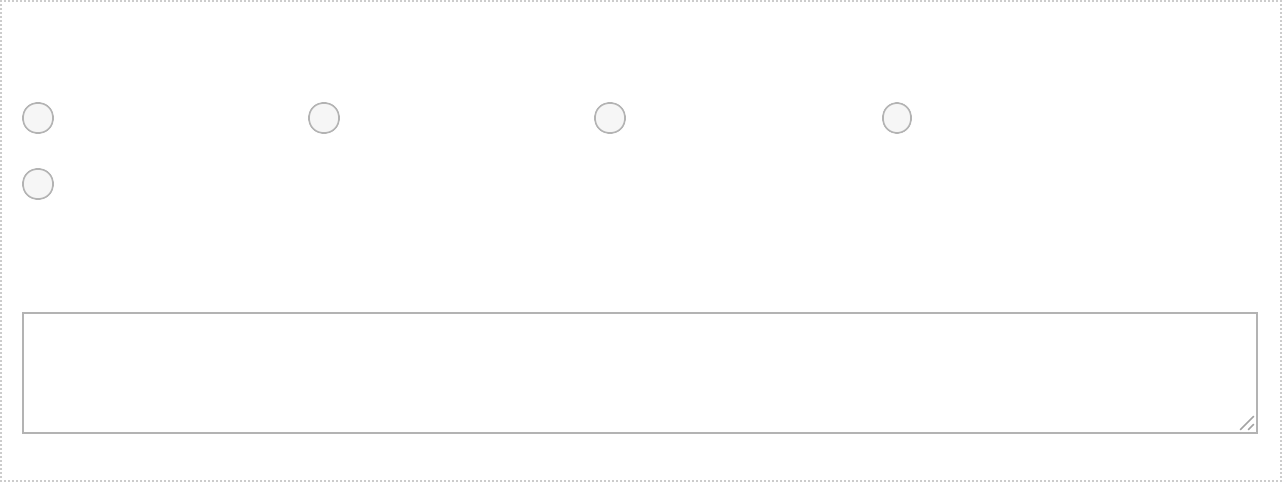


6. Hur bedömer du din egen teknikkunnighet generellt i vardagen?

Låg

Ganska låg

Varken hög eller låg

Ganska hög

Hög

Kommentar

# 


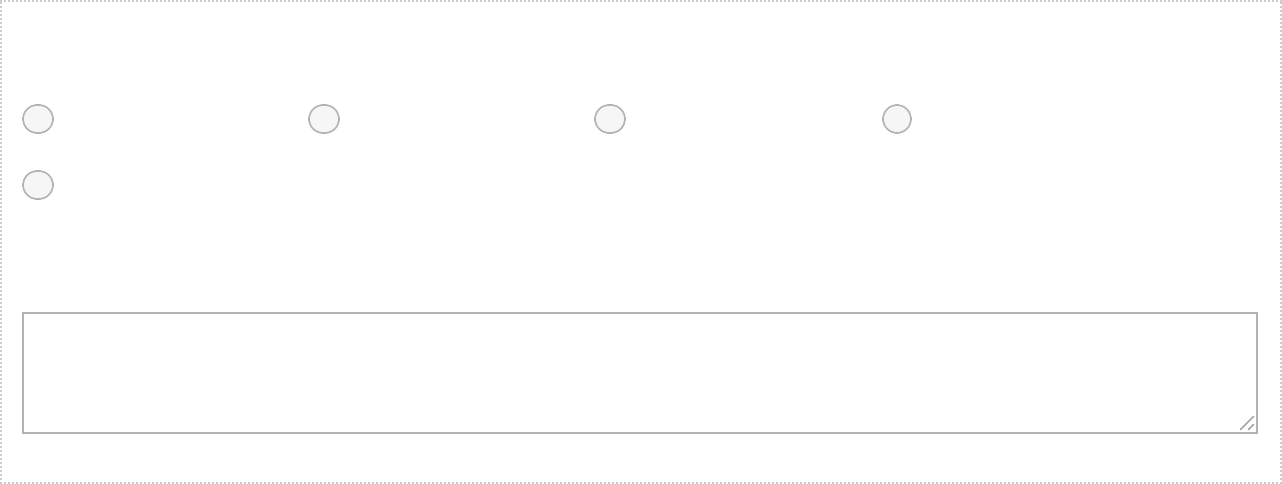


7. Hur bedömer du din egen teknikkunnighet generellt i arbetet?

Låg

Ganska låg

Varken hög eller låg

Ganska hög

Hög

Kommentar

# 


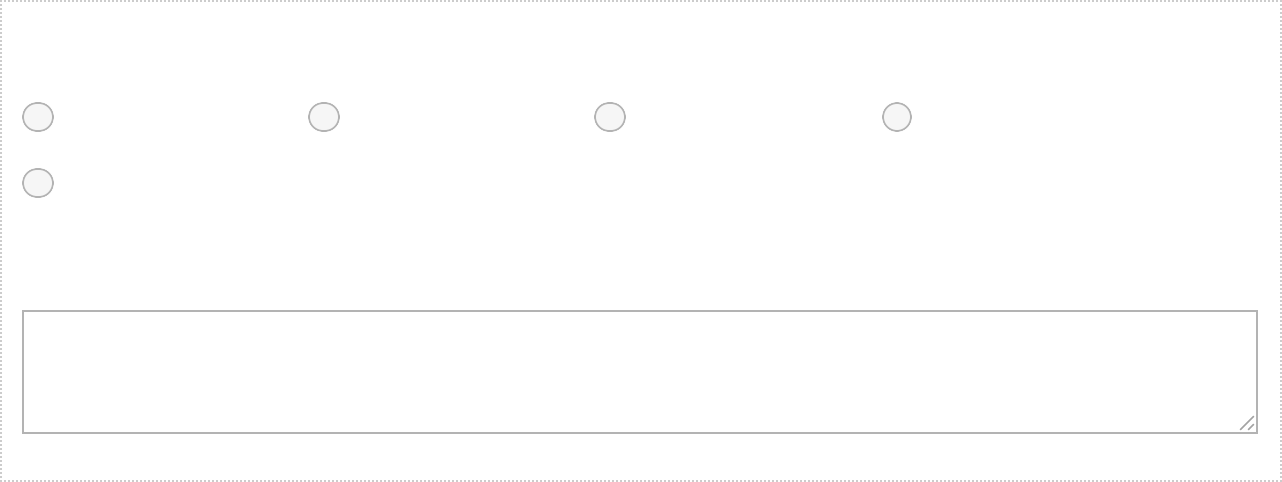


8. Hur bedömer du din egen kunskap om AI, artificiell intelligens?

Låg

Ganska låg

Varken hög eller låg

Ganska hög

Hög

Kommentar

# 


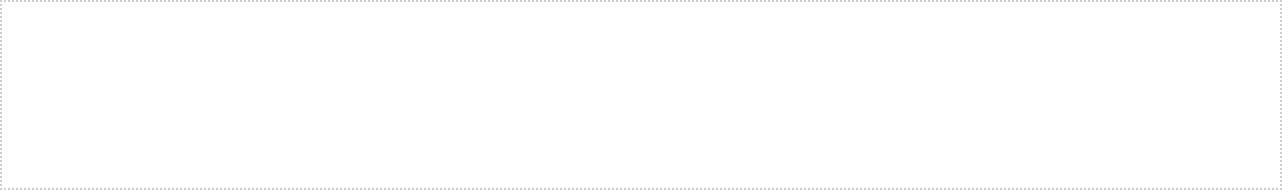


Inställning till artificiell intelligens i arbetet

# 


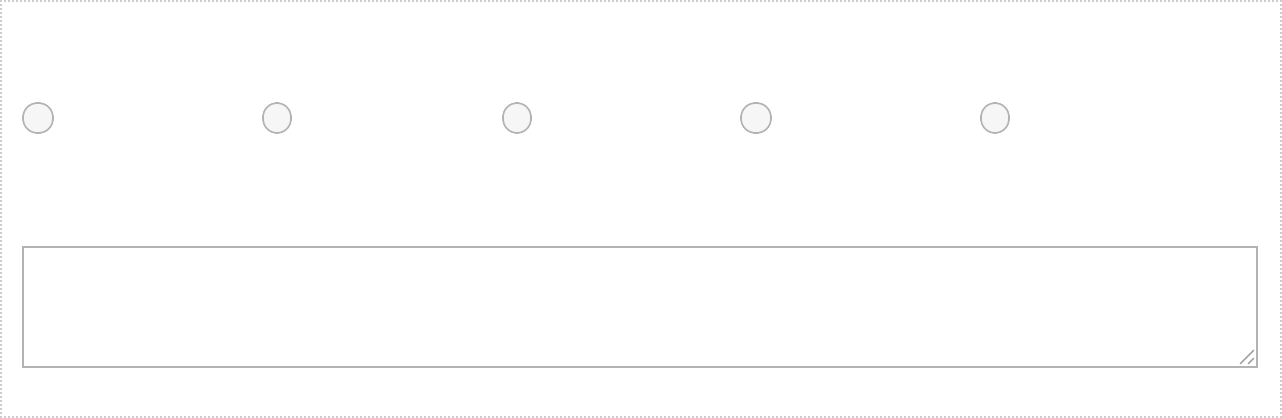


9. Vilken är din inställning till användning av AI-stödd mammografiscreening?

Negativ

Ganska negativ

Tveksam

Ganska positiv

Positiv

Kommentar


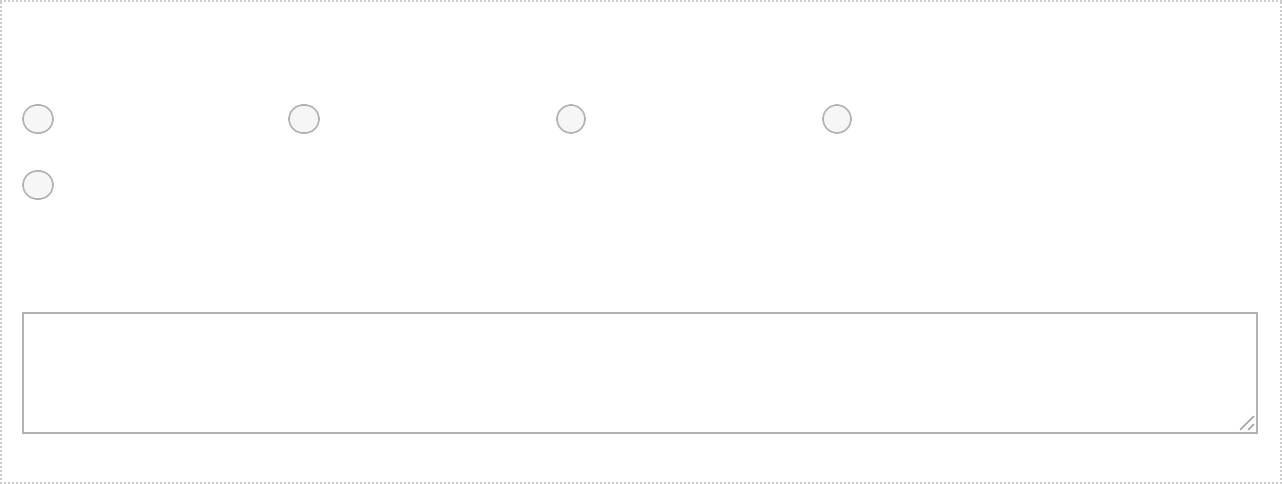


10. Anser du att det finns potentiella vinster med AI-stödd mammografiscreening?

I låg grad

I ganska låg grad

Tveksam

I ganska hög grad

I hög grad

Kommentar

# 


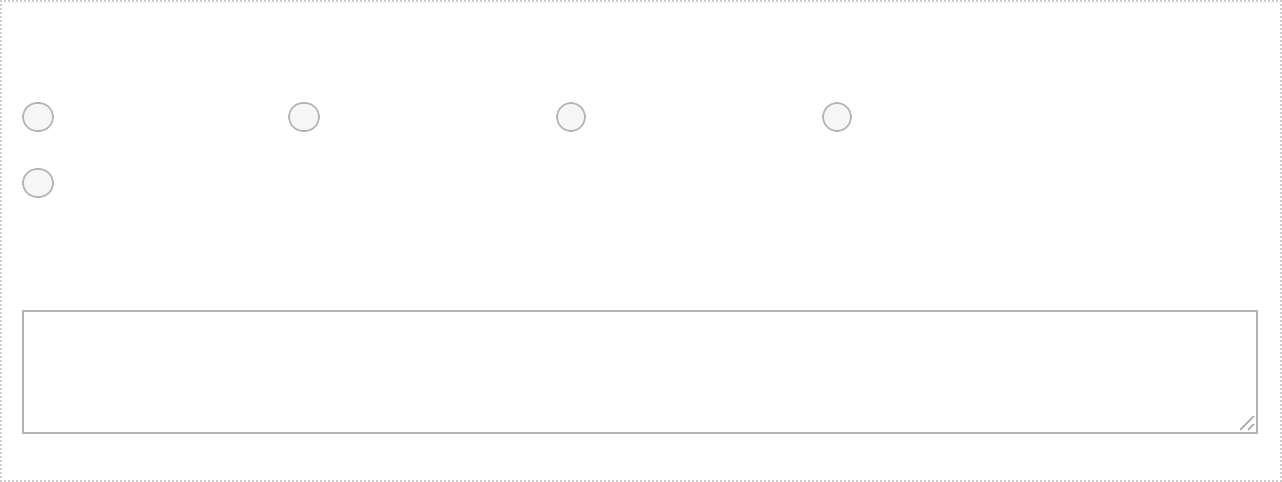


11. Anser du att det finns potentiella risker med AI-stödd mammografiscreening?

I låg grad

I ganska låg grad

Tveksam

I ganska hög grad

I hög grad

Kommentar

# 

# 


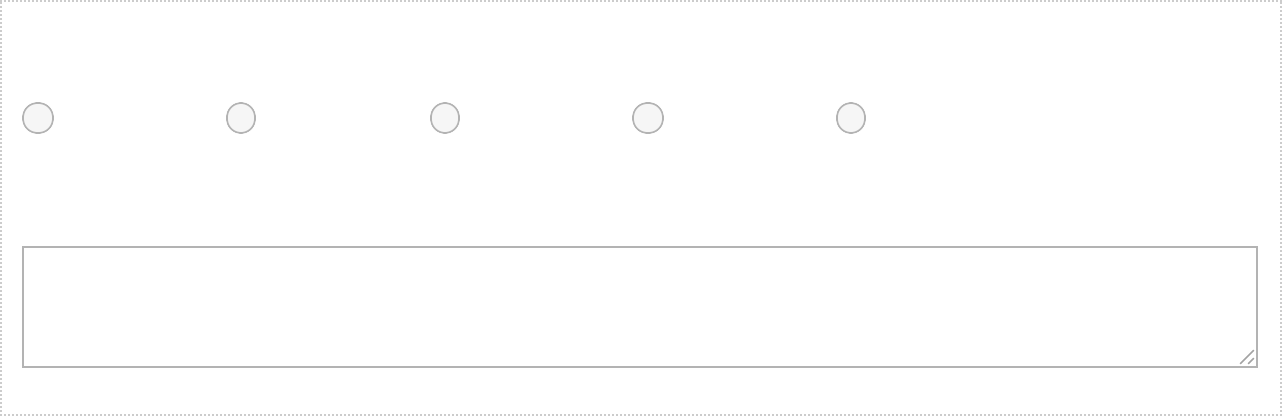


12. Vilken erfarenhet har du av att använda AI i ditt arbete som mammografiläkare?

Ingen

Liten

Ganska liten

Ganska stor

Stor

Kommentar

# 


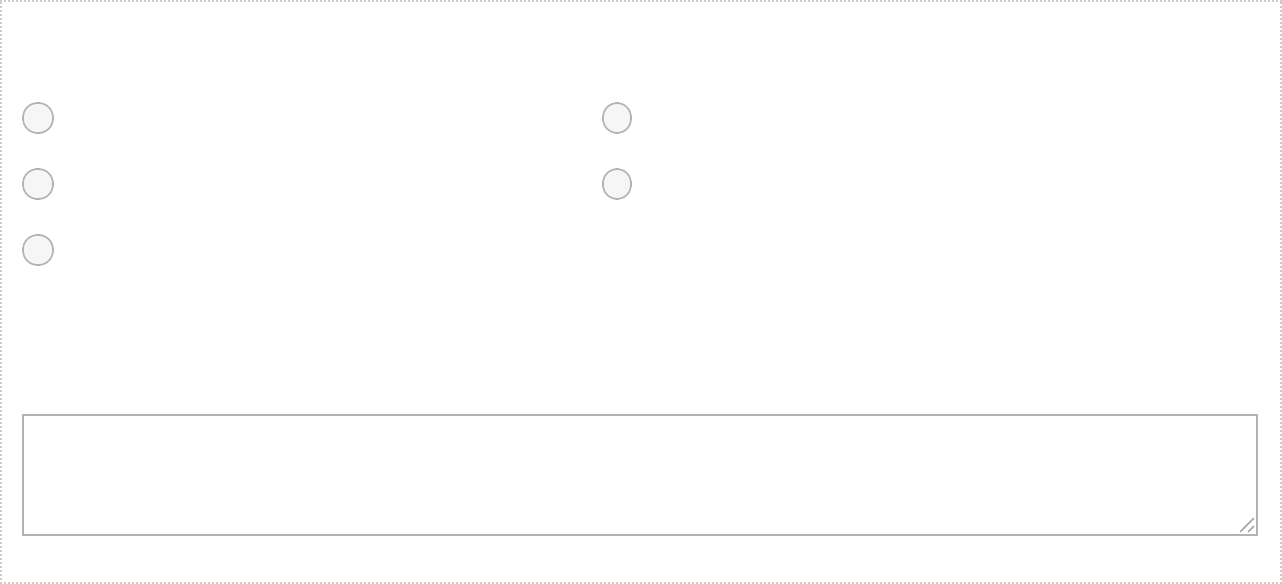


13. Hur skulle du vilja använda AI i mammografiscreening?

AI som verktyg för triage

AI som enda granskare

AI ersätter en granskare vid dubbelgranskning

AI används i tillägg till dubbelgranskning

Inte alls

Det finns flera olika möjligheter att använda AI i screeningen. Skriv gärna egna förslag eller kommentarer till

ovan nämnda förslag, t.ex. hur du skulle vilja kombinera alternativen.

# 


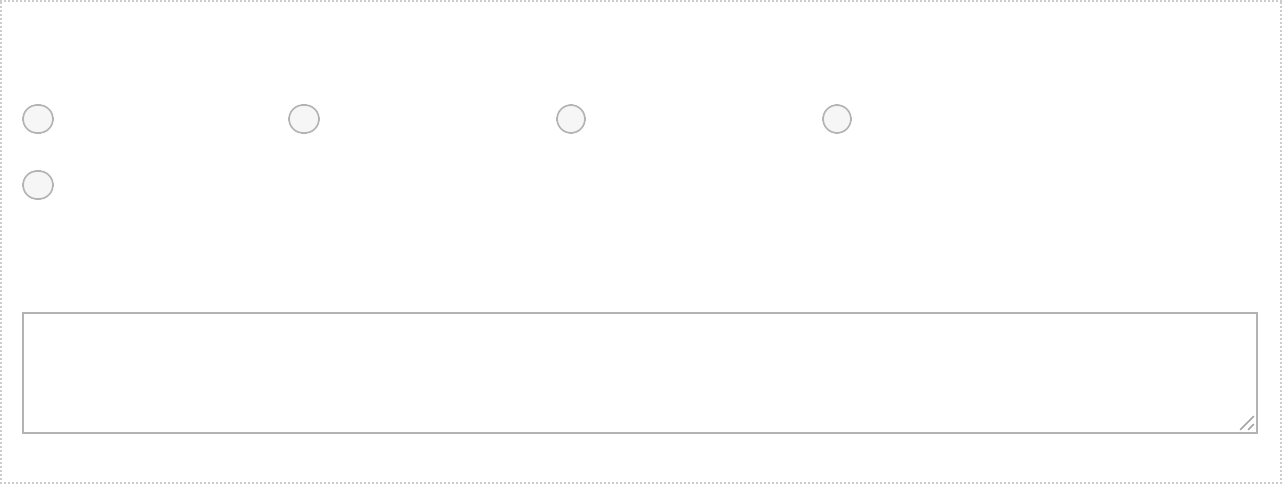


14. I vilken grad skulle du lita på AI-verktygs bedömningar?

I låg grad

I ganska låg grad

Tveksam

I ganska hög grad

I hög grad

Kommentar

# 


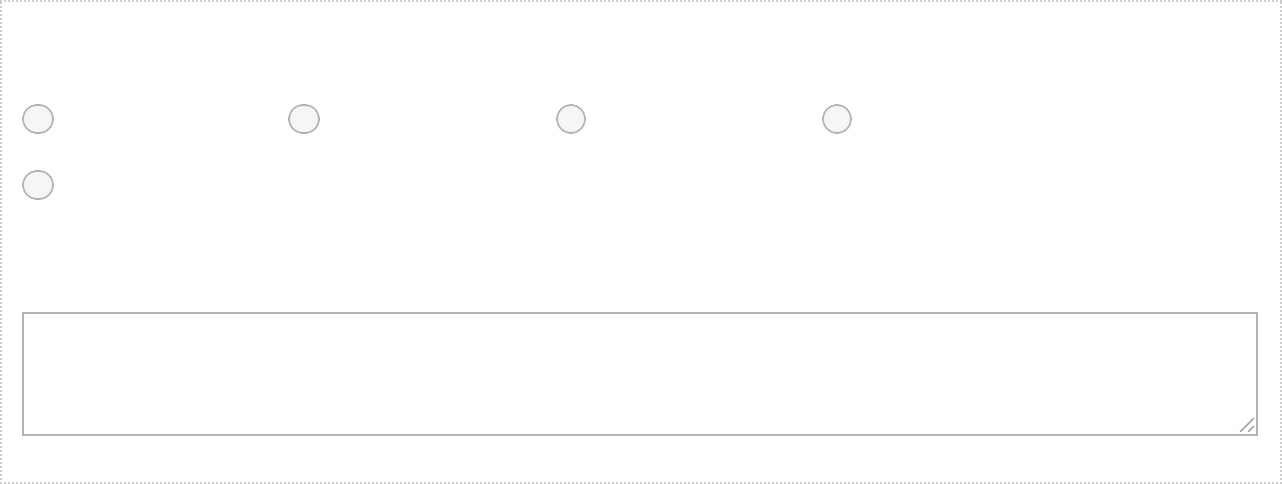


15. Tror du att det finns en risk att radiologer har en övertro till AI-verktygs bedömningar?

I låg grad

I ganska låg grad

Tveksam

I ganska hög grad

I hög grad

Kommentar


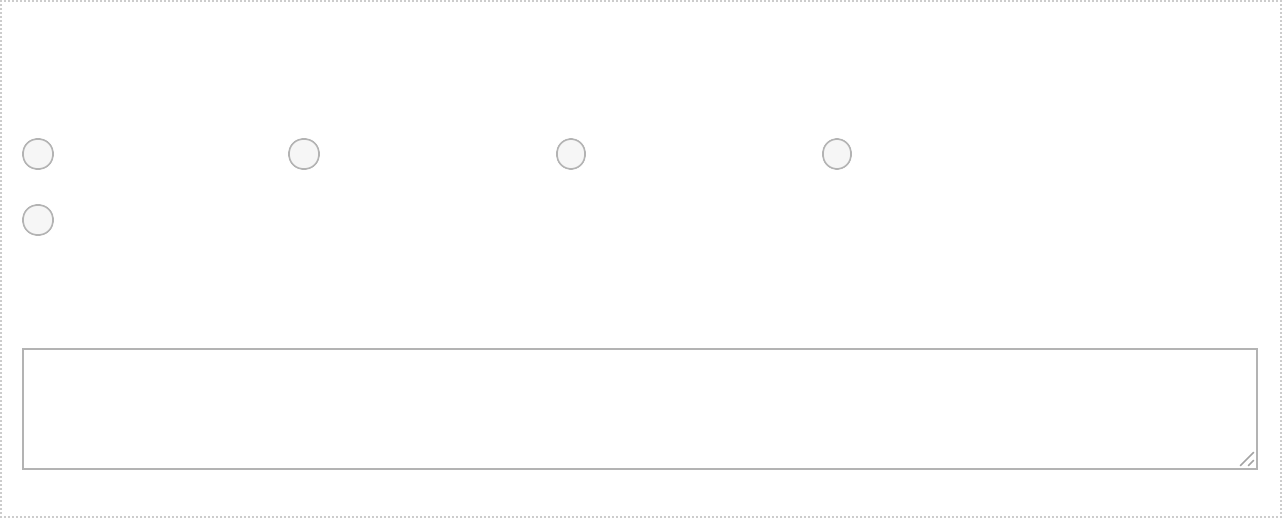


16. Skulle din tillit till AI-verktyget vara större ifall radiologernas bedömningar bidrog till fortsatt träning av AI-

verktyget efter klinisk implementering?

I låg grad

I ganska låg grad

Tveksam

I ganska hög grad

I hög grad

Kommentar


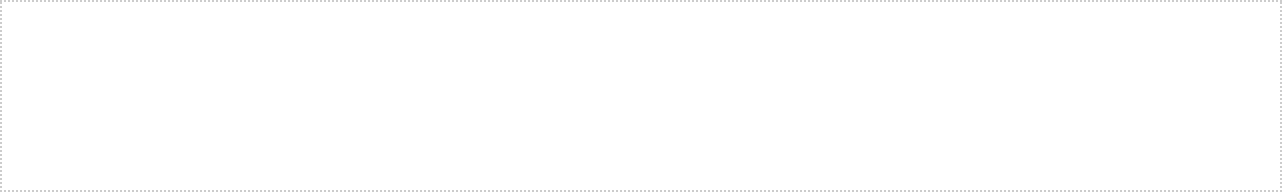


**Vem anser du bär ansvaret för bedömningar gjorda vid AI-stödd mammografiscreening?**


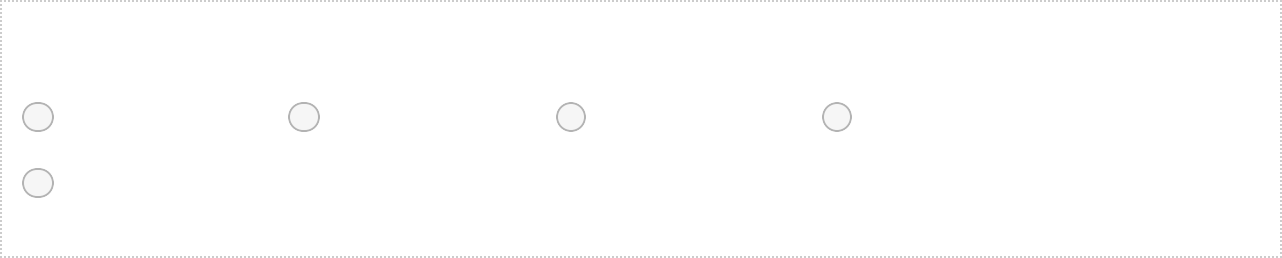


17. Den enskilda radiologen (ex. i egenskap av medgranskare)

I låg grad

I ganska låg grad

Tveksam

I ganska hög grad

I hög grad

# 


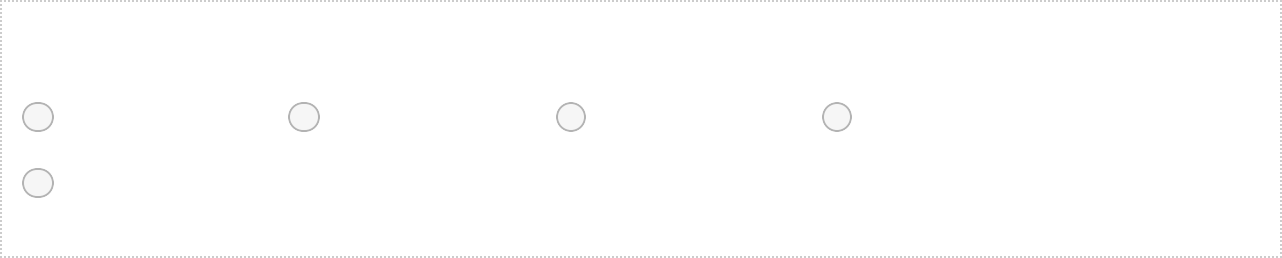


18. Vårdgivaren

I låg grad

I ganska låg grad

Tveksam

I ganska hög grad

I hög grad

# 


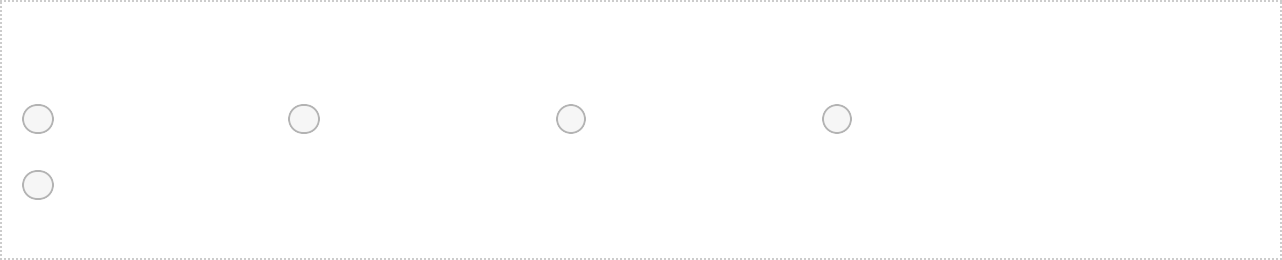


19. AI-verktyget

I låg grad

I ganska låg grad

Tveksam

I ganska hög grad

I hög grad

# 


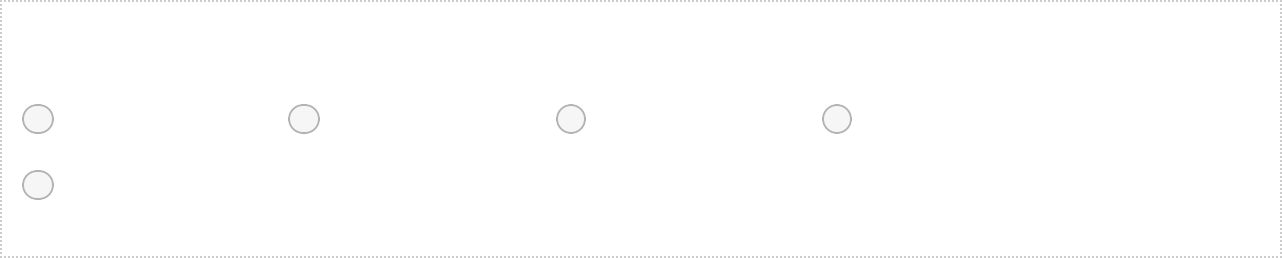


20. Utvecklare av AI-verktyget

I låg grad

I ganska låg grad

Tveksam

I ganska hög grad

I hög grad

# 


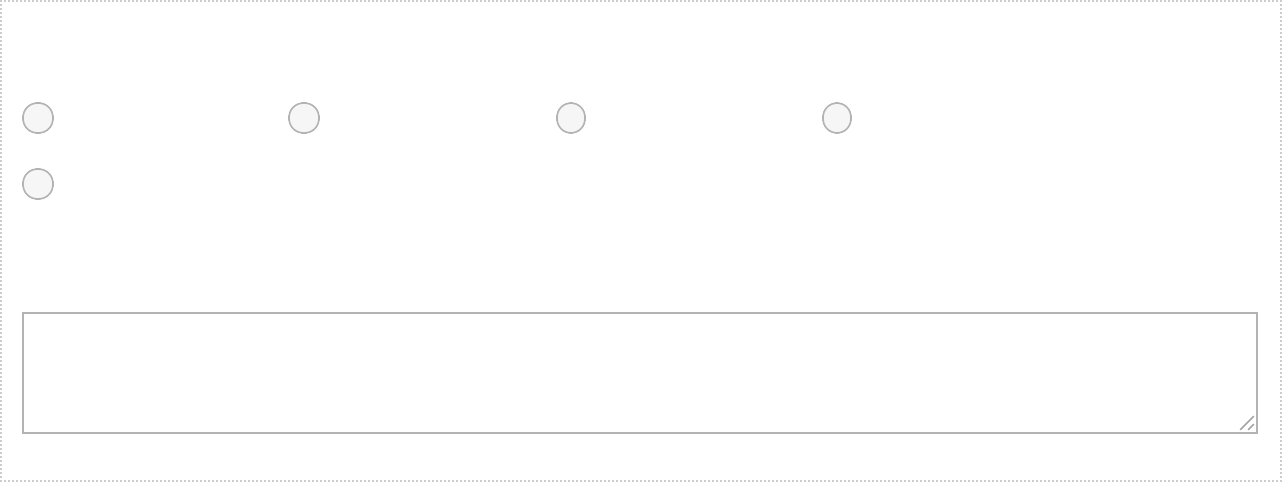


21. Delat ansvar

I låg grad

I ganska låg grad

Tveksam

I ganska hög grad

I hög grad

Om du anser att det är delat ansvar, specificera mellan vilka aktörer och varför:

# 


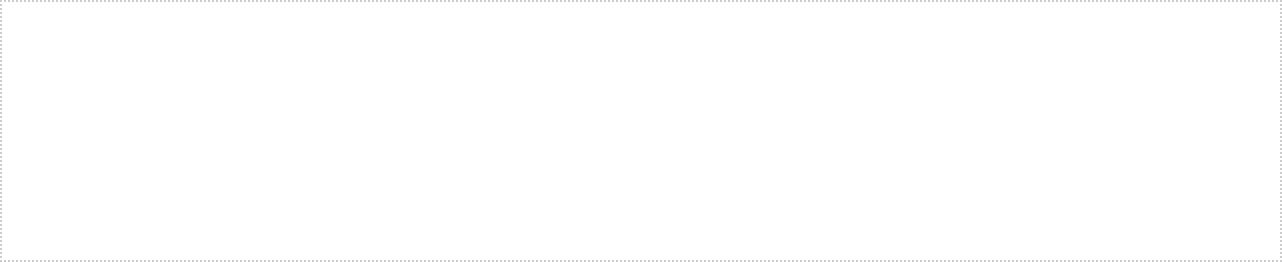


**Vem anser du bär ansvar för bedömningar gjorda vid mammografiscreening med enbart AI-granskning?**


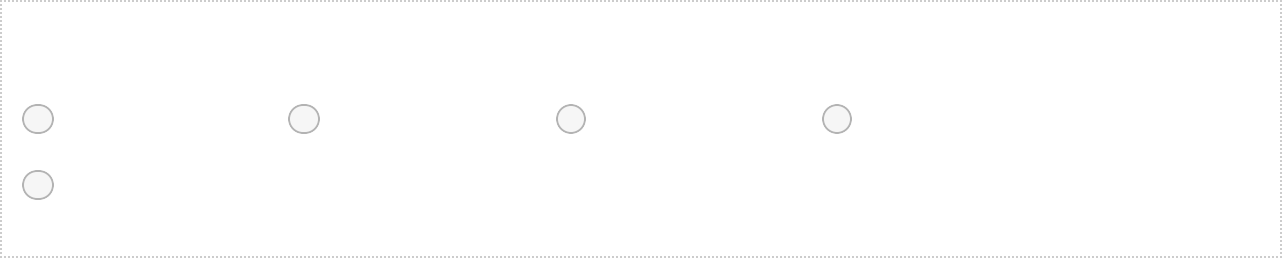


22. Den enskilda radiologen (ex. i egenskap av översyn av screeningprocessen)

I låg grad

I ganska låg grad

Tveksam

I ganska hög grad

I hög grad

# 


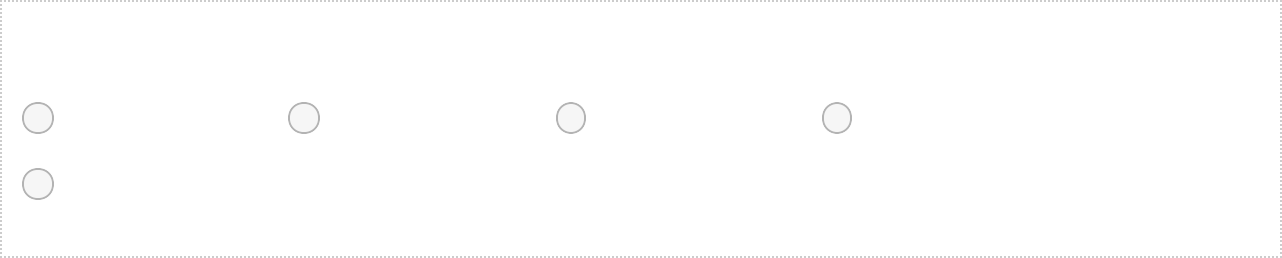


23. Vårdgivaren

I låg grad

I ganska låg grad

Tveksam

I ganska hög grad

I hög grad

# 


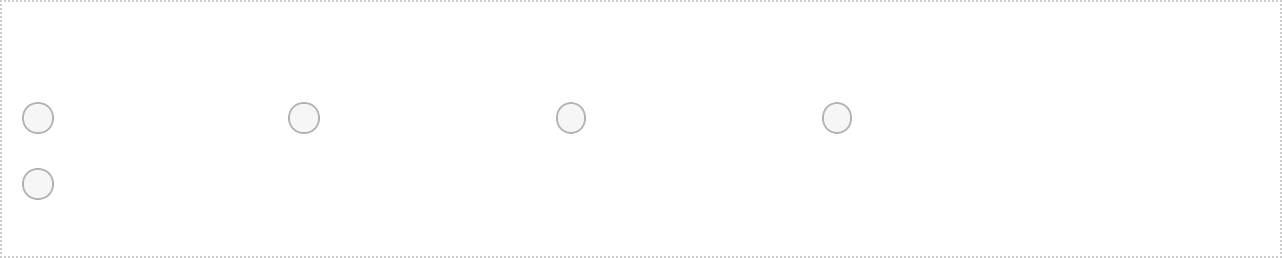


24. AI-verktyget

I låg grad

I ganska låg grad

Tveksam

I ganska hög grad

I hög grad

# 


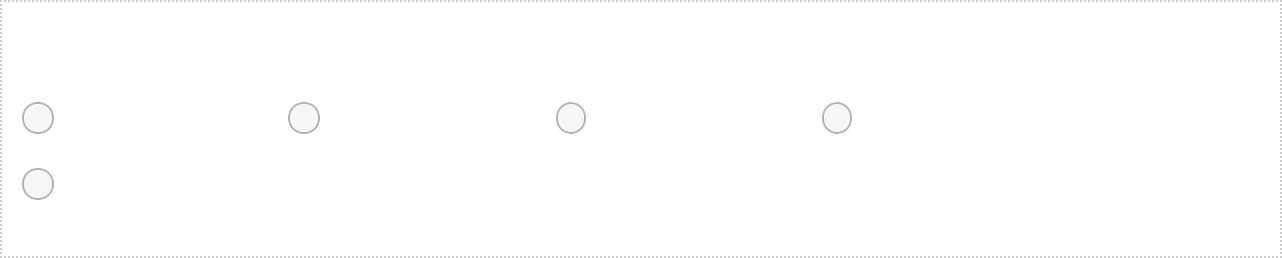


25. Utvecklare av AI-verktyget

I låg grad

I ganska låg grad

Tveksam

I ganska hög grad

I hög grad

# 


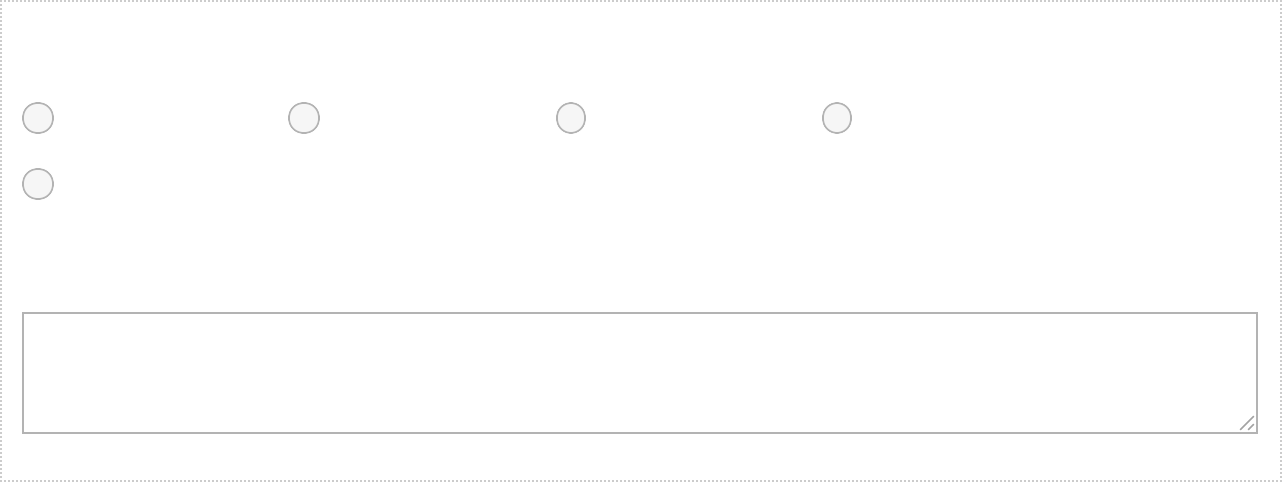


26. Delat ansvar

I låg grad

I ganska låg grad

Tveksam

I ganska hög grad

I hög grad

Om du anser att det är delat ansvar, specificera mellan vilka aktörer och varför:

# 


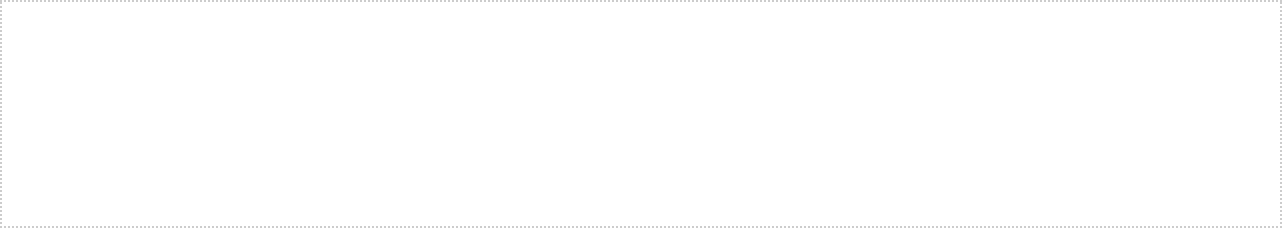


Om du använder/har använt AI i ditt arbete, anser du att du har fått den information du behöver för att kunna bedöma riktigheten i AI-verktygets bedömning:


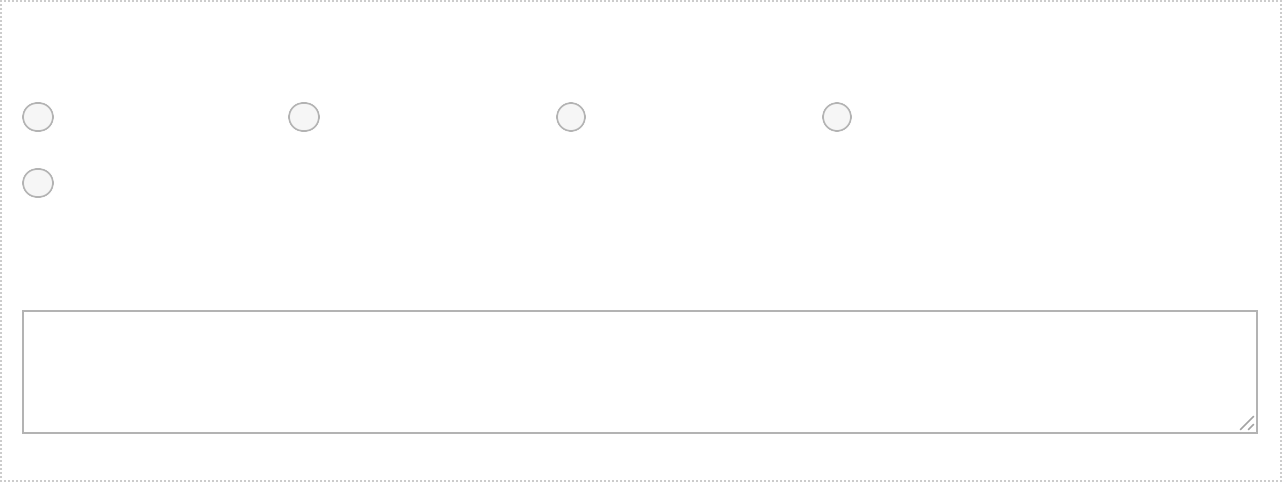


27. Vid användning av verktyget (information i användargränssnitt m.m.)?

I låg grad

I ganska låg grad

Tveksam

I ganska hög grad

I hög grad

Kommentar


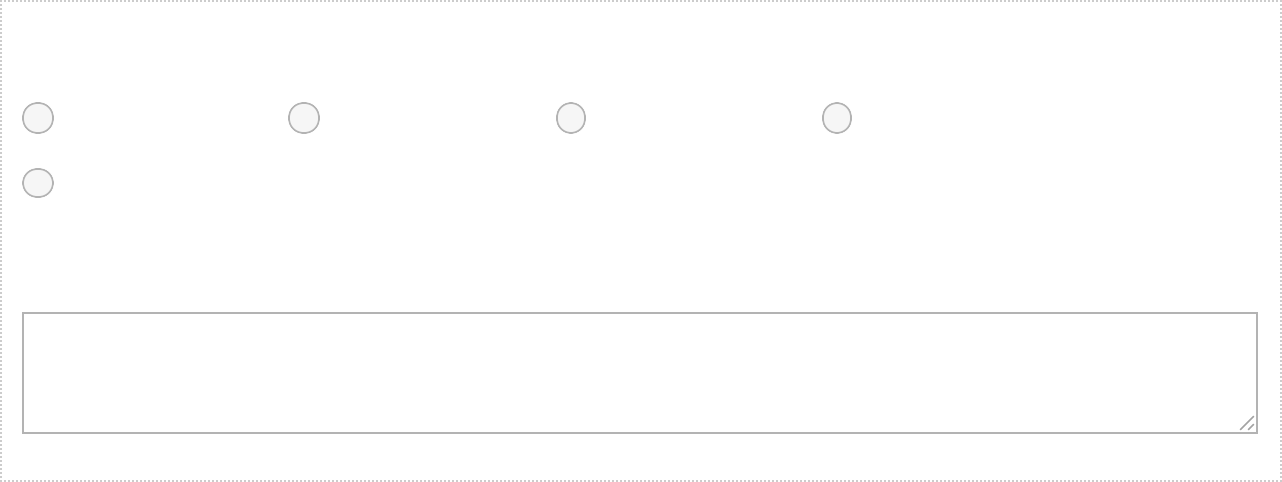


28. Inför användning (information från utvecklare m.m.)?

I låg grad

I ganska låg grad

Tveksam

I ganska hög grad

I hög grad

Kommentar


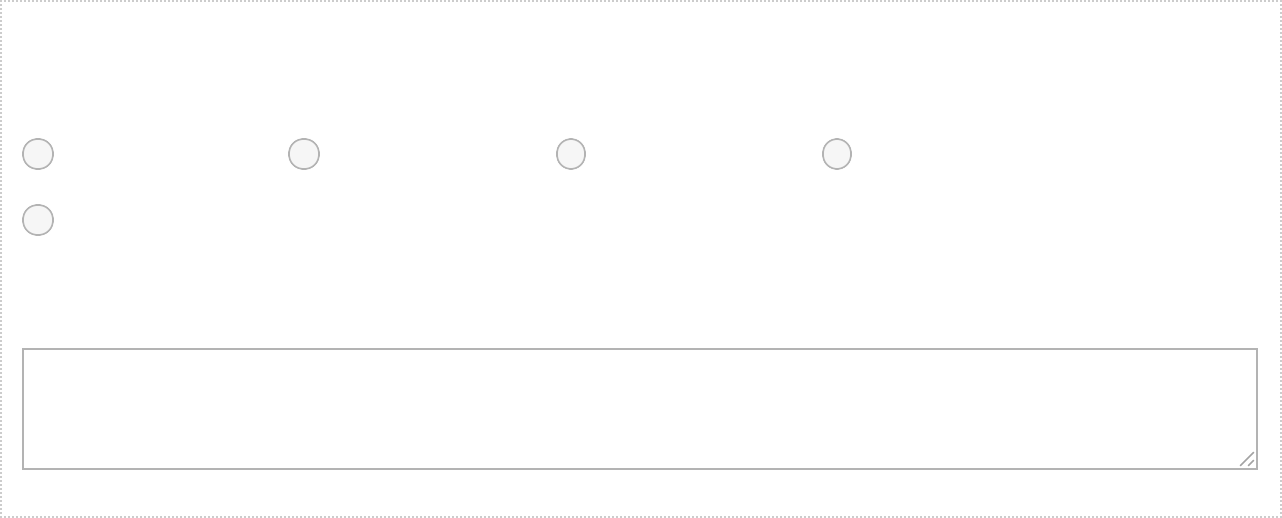


29. Om du skulle använda AI-verktyg i mammografiscreening, önskar du få information om hur det har

utvecklats (exempelvis vilken data som verktyget har tränats på)?

I låg grad

I ganska låg grad

Tveksam

I ganska hög grad

I hög grad

Kommentar

# 


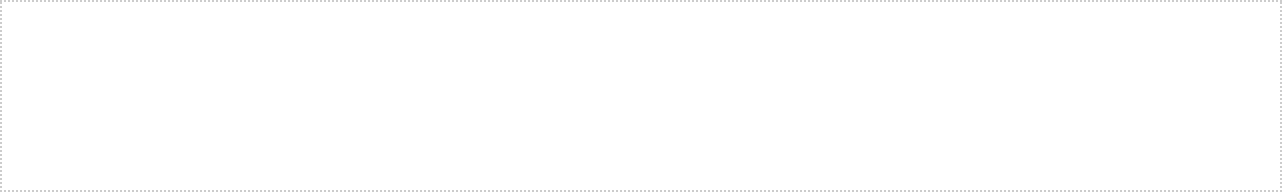


Tror du att följande information hade underlättat för dig att kunna avgöra tilliten till ett AI-verktygs bedömning:


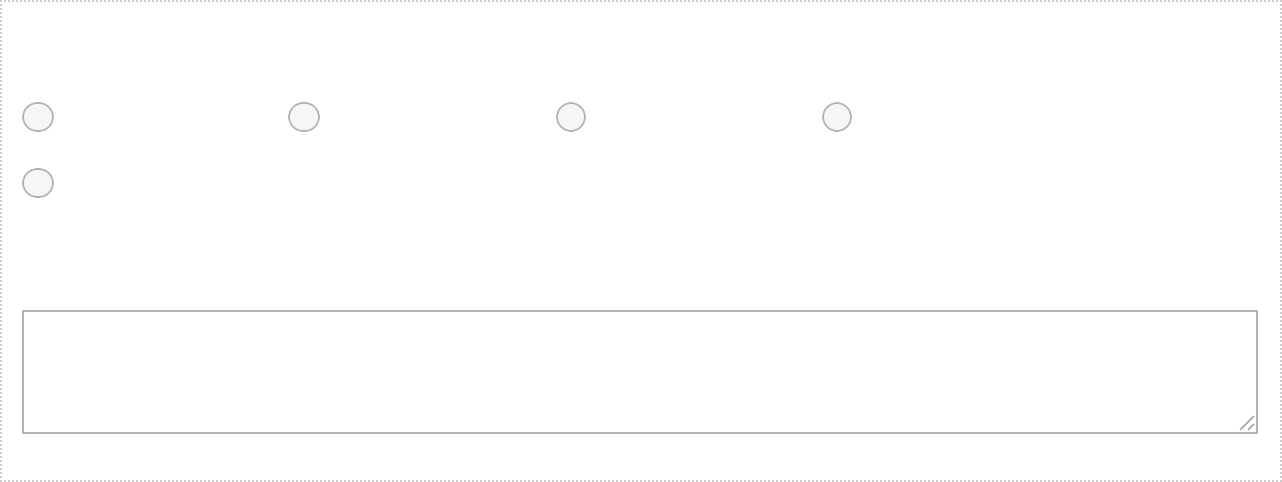


30. Information om vad i bilden som föranlett en viss riskgradering?

I låg grad

I ganska låg grad

Tveksam

I ganska hög grad

I hög grad

Kommentar

# 


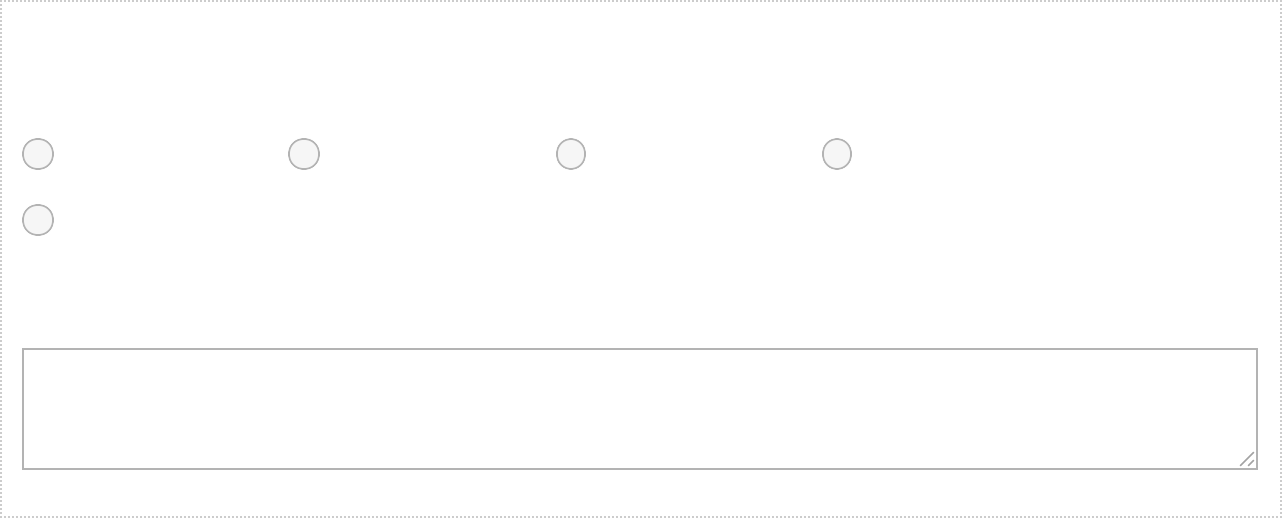


31. Information om vad och hur mycket i bilden som hade behövt vara annorlunda för att den istället skulle ha

graderats som en högre respektive lägre risknivå?

I låg grad

I ganska låg grad

Tveksam

I ganska hög grad

I hög grad

Kommentar

# 


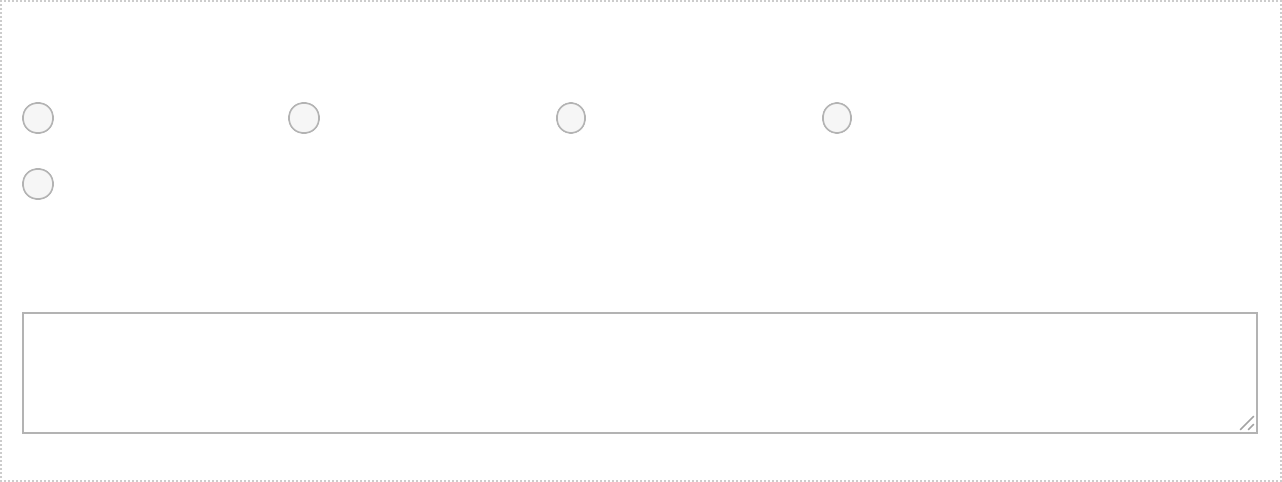


32. Information om bakomliggande kod/algoritmer?

I låg grad

I ganska låg grad

Tveksam

I ganska hög grad

I hög grad

Kommentar

# 


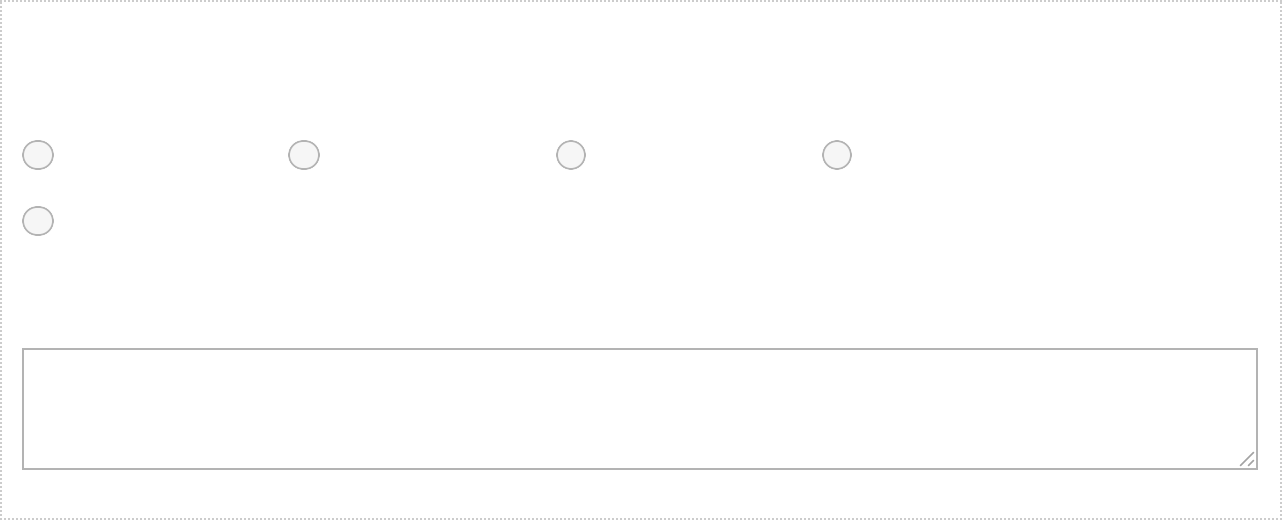


33. Information om vilka kompetenser (ex. bröstradiolog, datavetare, statistiker) som varit delaktiga i

utvecklingen av AI-verktyget?

I låg grad

I ganska låg grad

Tveksam

I ganska hög grad

I hög grad

Kommentar

# 


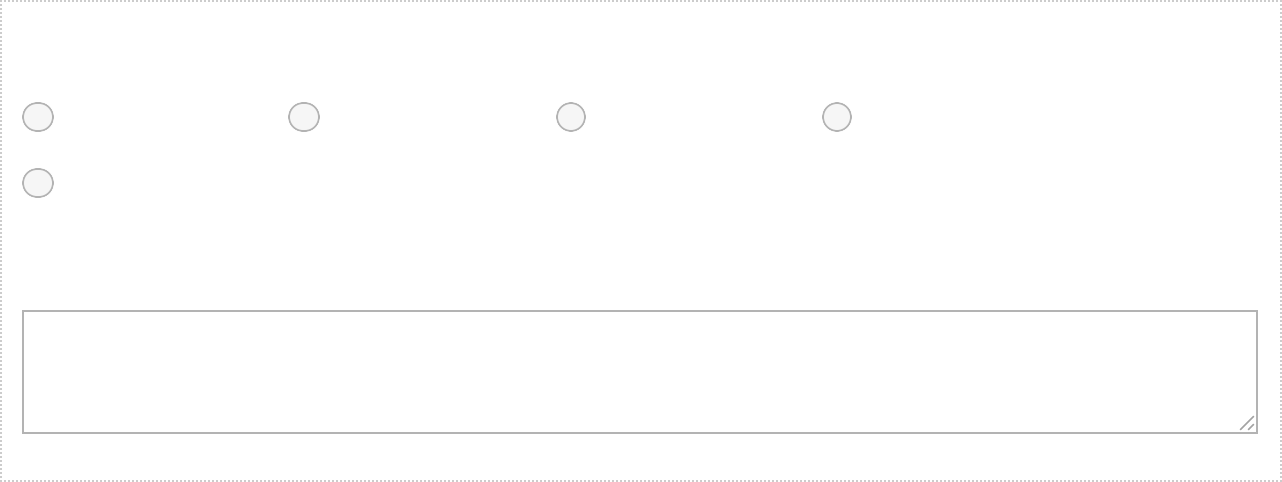


34. Information om vilken data AI-modellen har tränats på?

I låg grad

I ganska låg grad

Tveksam

I ganska hög grad

I hög grad

Kommentar

# 


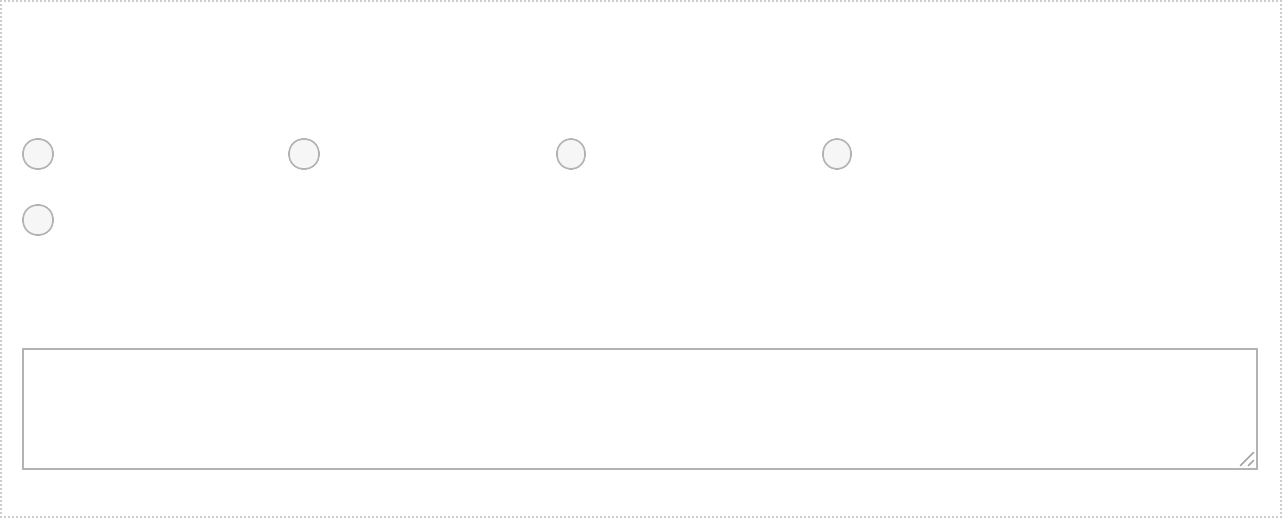


35. Information om hur eventuell uppmärkning av träningsdata gått till (t.ex. hur mammografier med cancer har

identifierats och annoterats inför träning av AI)?

I låg grad

I ganska låg grad

Tveksam

I ganska hög grad

I hög grad

Kommentar

# 


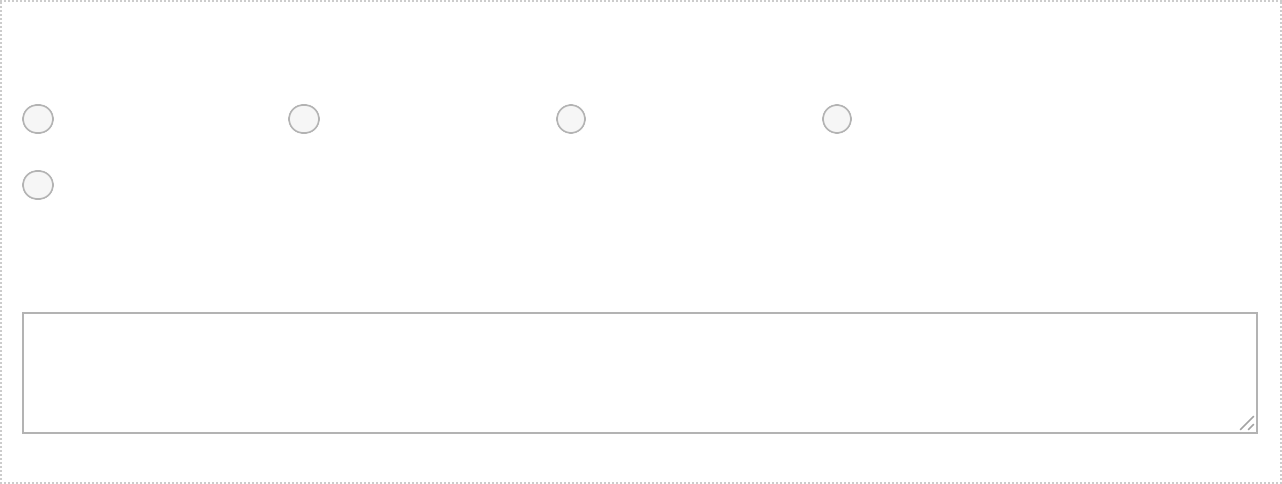


36. Information om verktygets fortsatta lärande efter klinisk implementering?

I låg grad

I ganska låg grad

Tveksam

I ganska hög grad

I hög grad

Kommentar

# 


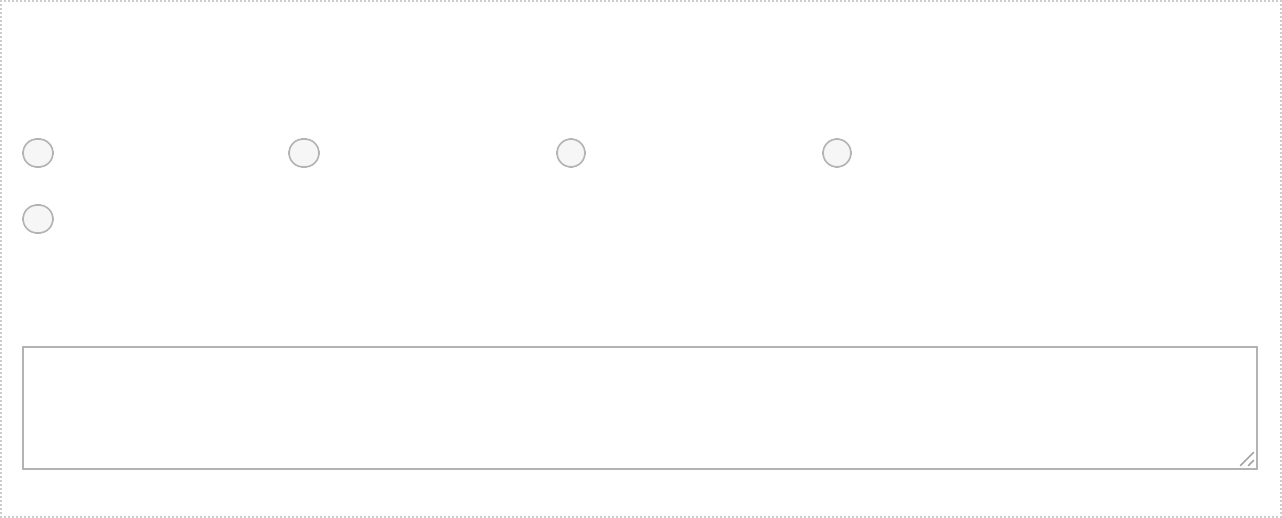


37. Tror du att du skulle ha kompetens att avgöra om ett AI-verktygs bedömning vid mammografiscreening är

korrekt?

I låg grad

I ganska låg grad

Tveksam

I ganska hög grad

I hög grad

Kommentar

# 


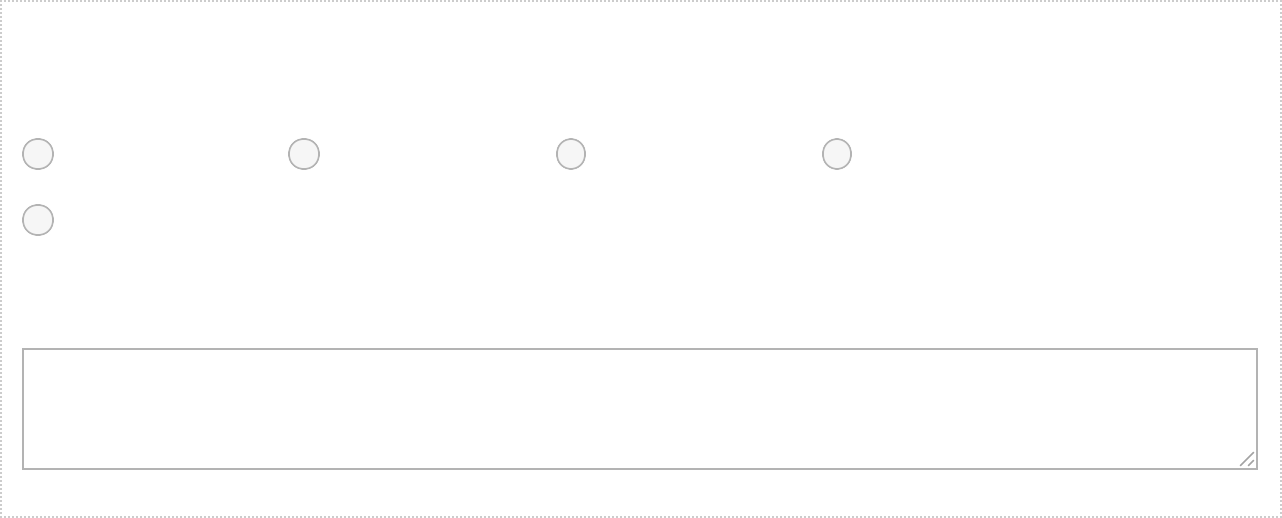


38. Tror du att det finns risk att de data som AI-verktyg har tränats på vid utveckling inte är representativ för

den befolkningsgrupp som verktyget appliceras på?

I låg grad

I ganska låg grad

Tveksam

I ganska hög grad

I hög grad

Kommentar

# 


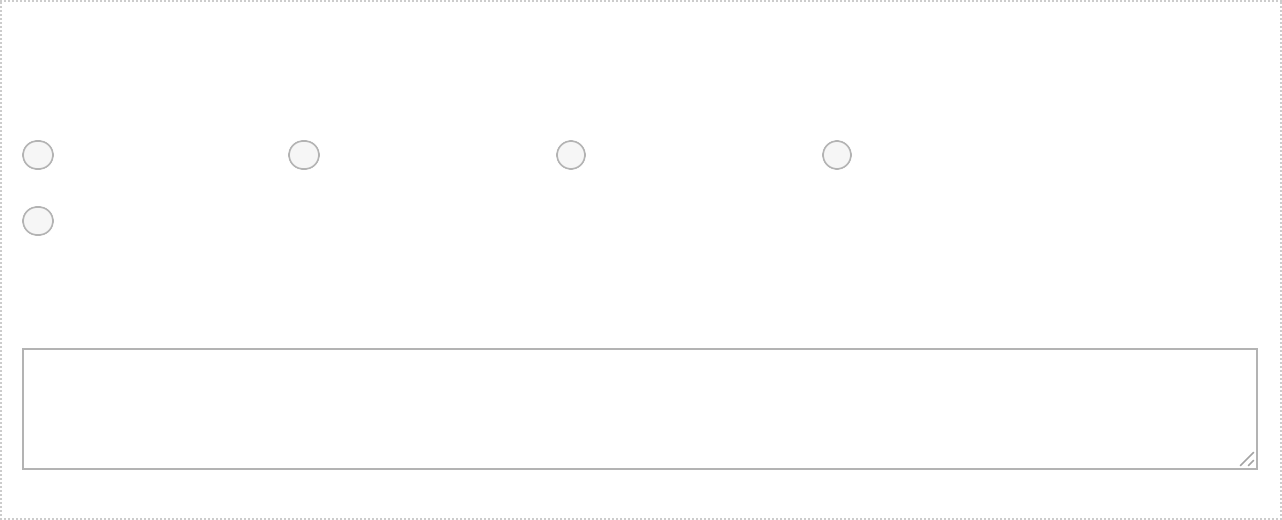


39. Tror du att det finns en risk att AI-verktyg presterar sämre än radiologer vid mammografiscreening för vissa

riskgrupper eller specifika typer av fall?

I låg grad

I ganska låg grad

Tveksam

I ganska hög grad

I hög grad

Beskriv i så fall vilka typer av riskgrupper/fall:

# 


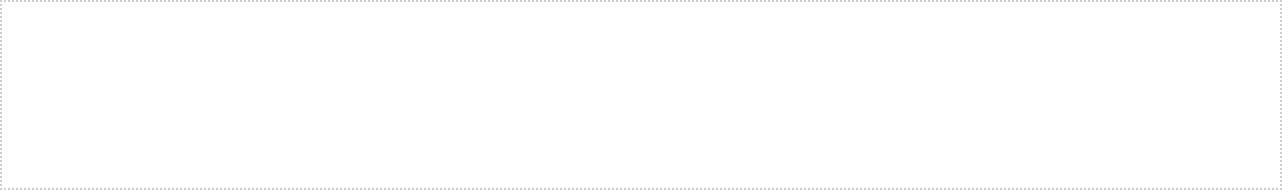


Professionens utveckling


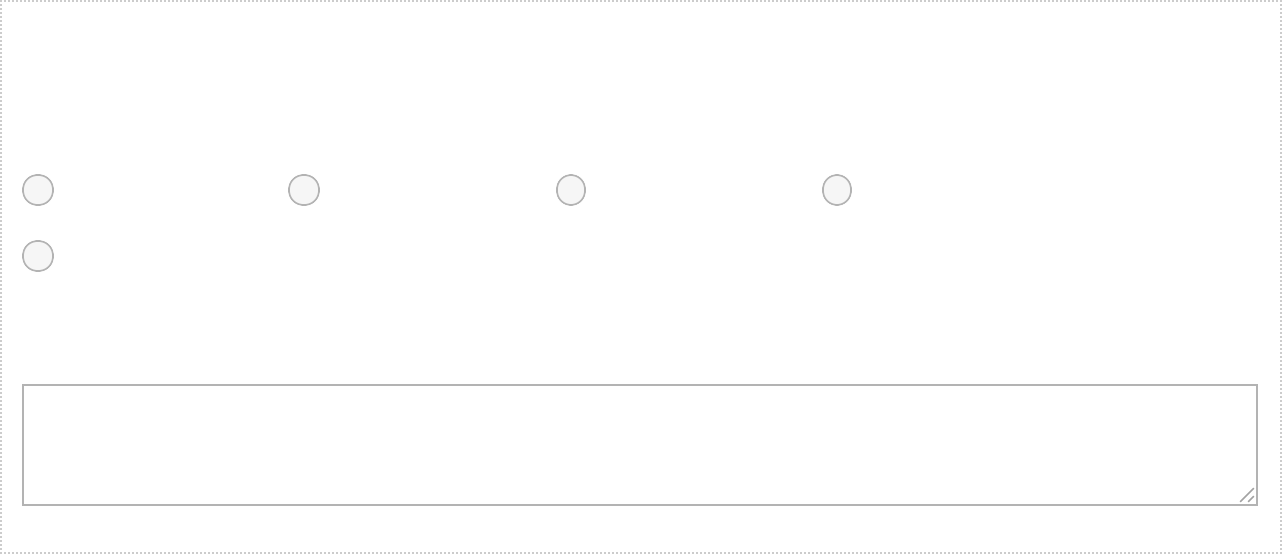


40. Om du skulle använda AI-verktyg i ditt arbete med mammografiscreening, skulle du önska att din utvärdering av verktygets bedömning skulle användas för att fortsätta träna och förbättra verktygets

precision?

I låg grad

I ganska låg grad

Tveksam

I ganska hög grad

I hög grad

Kommentar

# 


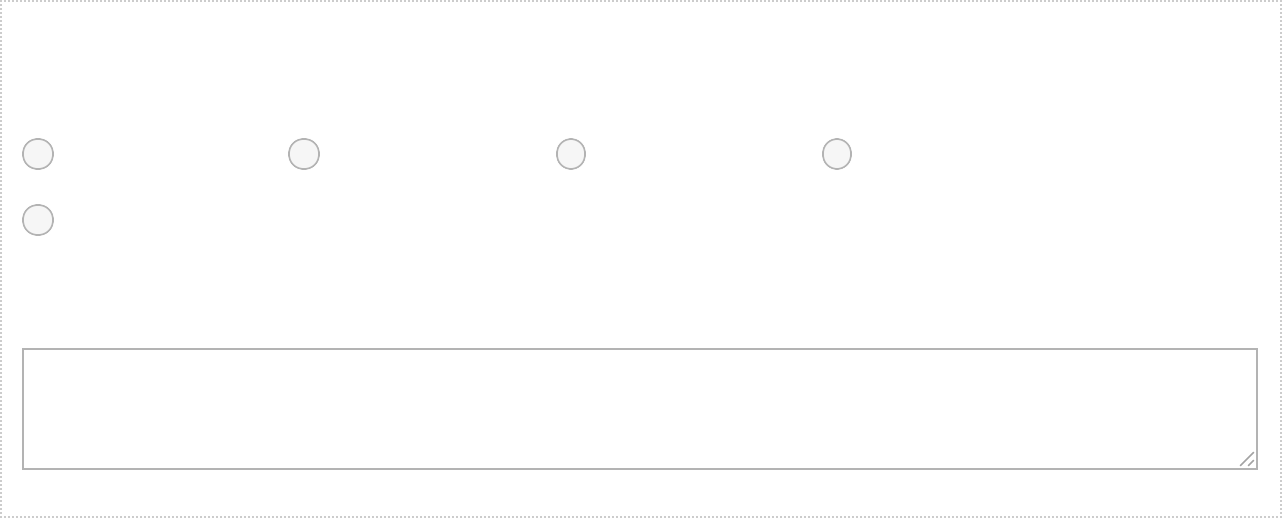


41. Anser du att det finns betydande skillnader i att implementera AI-verktyg i mammografiscreening jämfört

med annan tidigare teknikutveckling (t.ex. digital mammografi, tomosyntes)?

I låg grad

I ganska låg grad

Tveksam

I ganska hög grad

I hög grad

Kommentar

# 


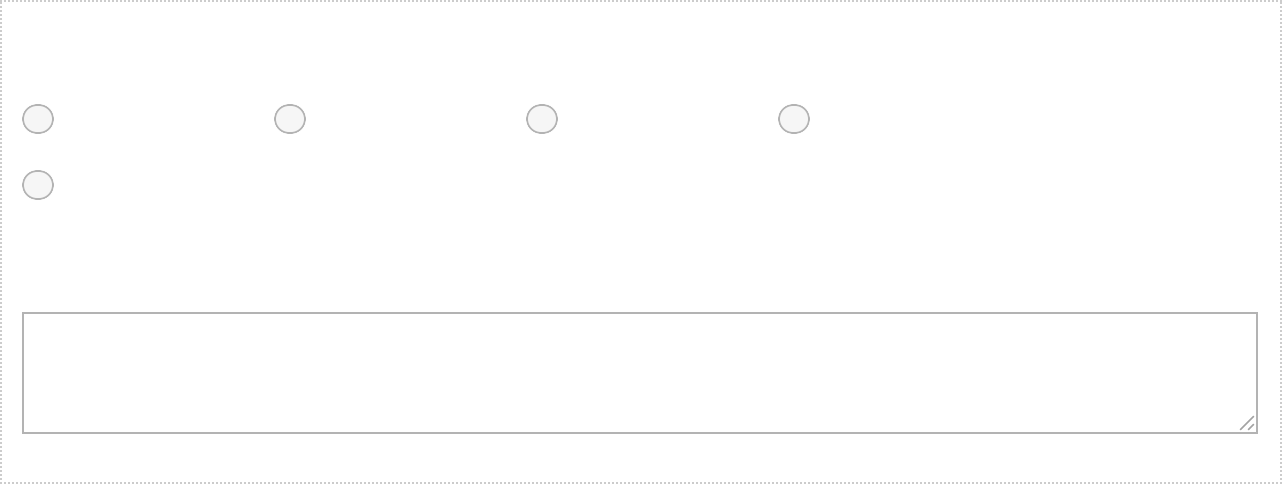


42. Hur tror du att mammografiläkarens roll skulle förändras om AI-stödd mammografiscreening införs?

Försvagas

Försvagas något

Inte alls

Stärkas något

Stärkas

Kommentar

# 


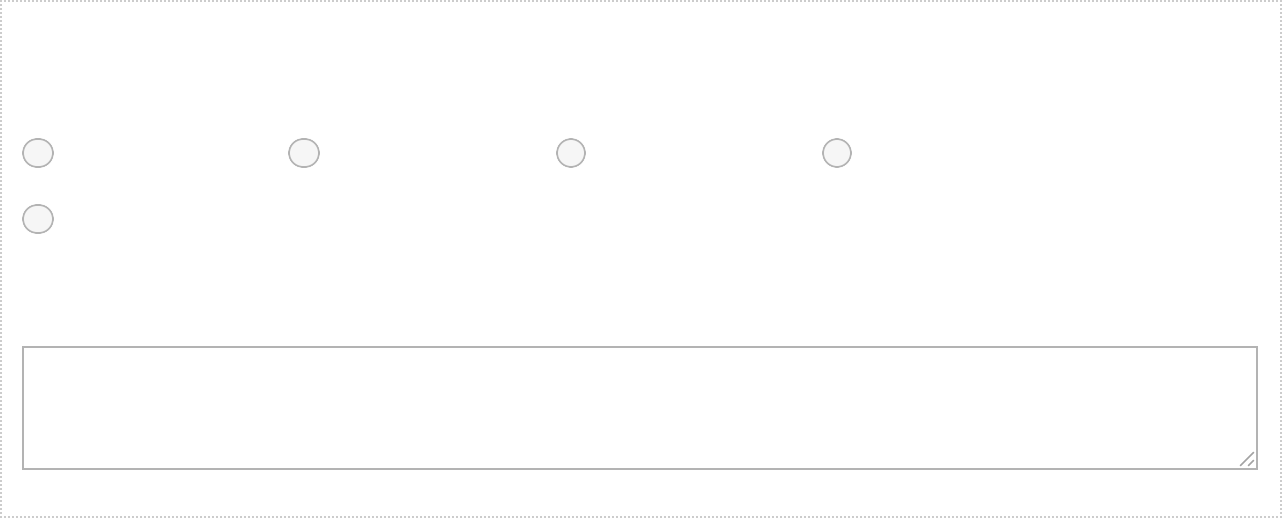


43. Tror du att införande av AI-stödd mammografiscreening skulle göra det lättare att rekrytera nya

bröstradiologer?

I låg grad

I ganska låg grad

Tveksam

I ganska hög grad

I hög grad

Kommentar

# 


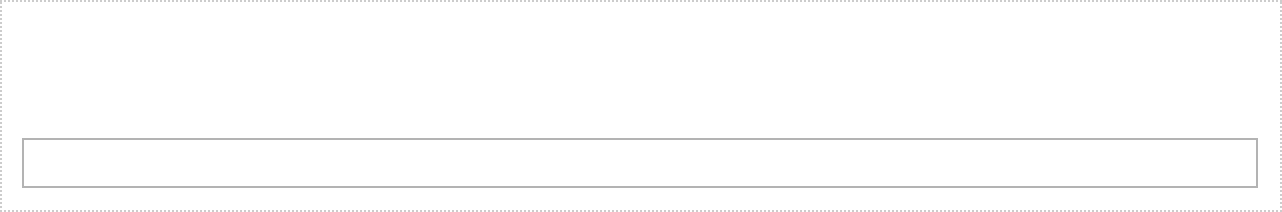


44. Tror du att införande av AI-stödd mammografiscreening skulle påverka relationen mellan läkare/sjukvård och individer som deltar i screening? I så fall, hur?


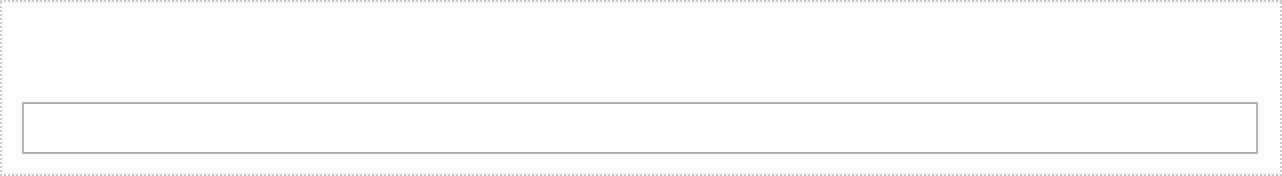


45. Hur tror du att professionen kommer att utvecklas, med tanke på rådande teknikutveckling med AI?

**Survey: Breast Radiologists’ Views on the use of Artificial Intelligence in Mammography Screening**


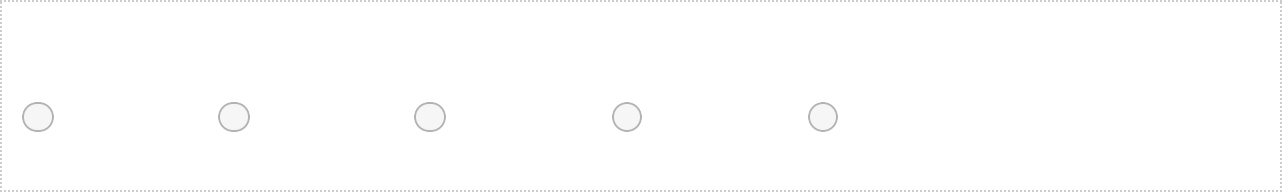


1. Age

<31 years

31-40 years

41-50 years

51-60 years

>60 years

# 


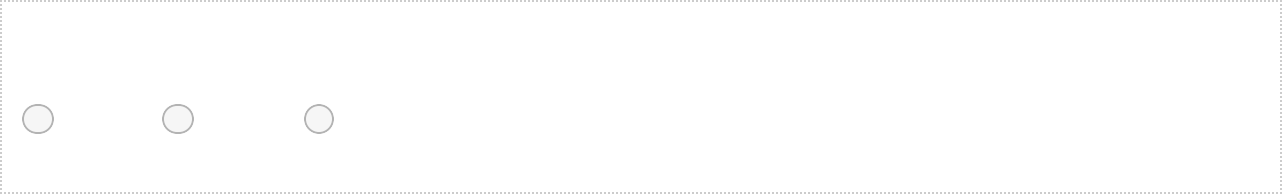


2. Gender

Female

Male

Other




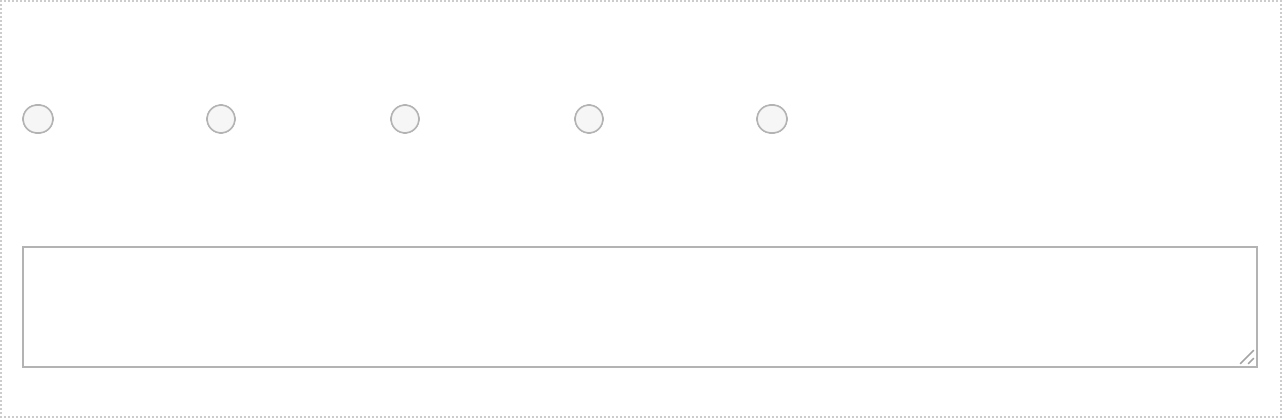


3. How long experience do you have of working with breast radiology?

<5 years

5-10 years

11-20 years

21-30 years

>30 years

Comments

# 


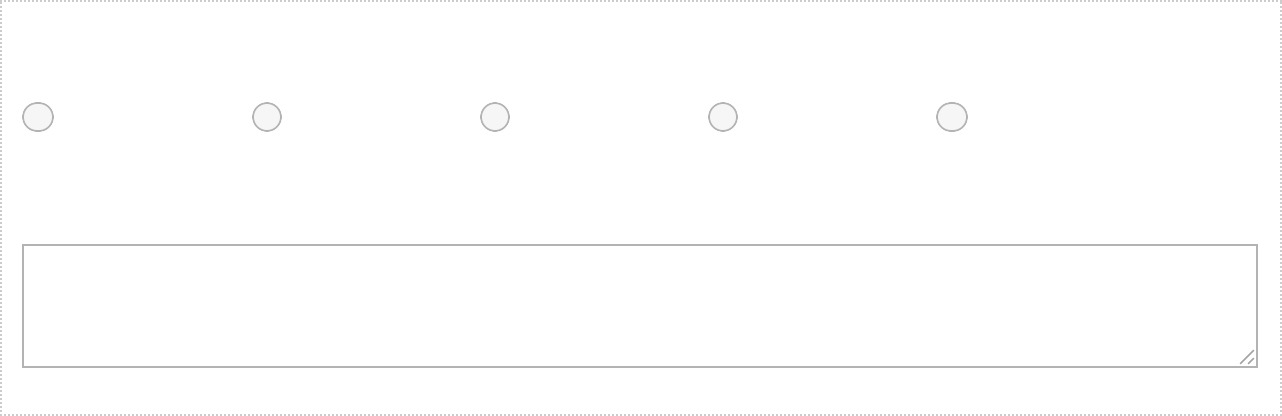


4. How many screening exams do you approximately review per year?

None

<2000

2 000 - 5 000

5 000 - 10 000

>10 000

Comments

# 


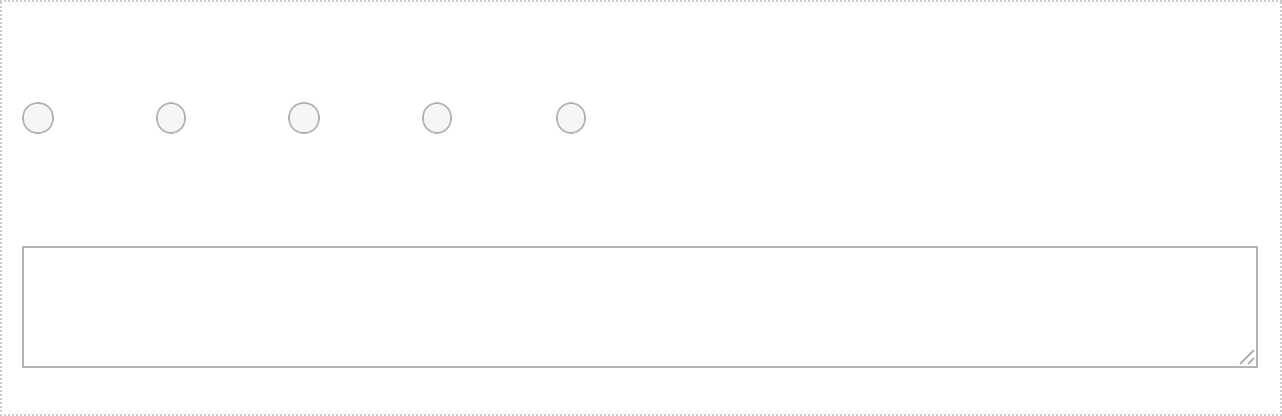


5. Do you experience difficulties finding the time to perform the screen-reading?

Never

Seldom

Some-times

Often

Always

Comments


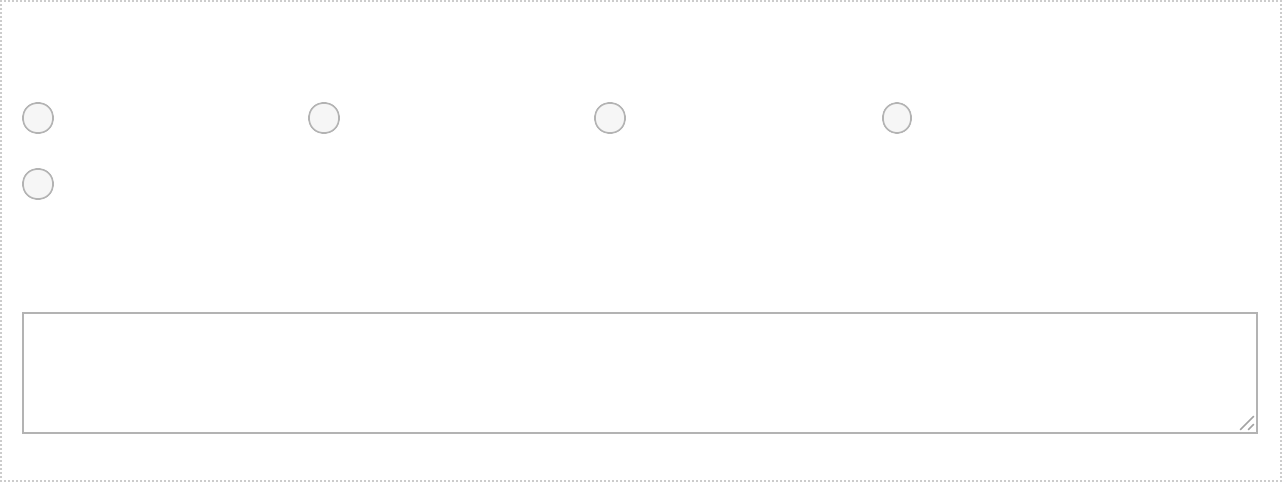


6. What is your estimated level of literacy of technology in everyday life?

Low

Somewhat low

Neither high nor low

Somewhat high

High

Comments

# 


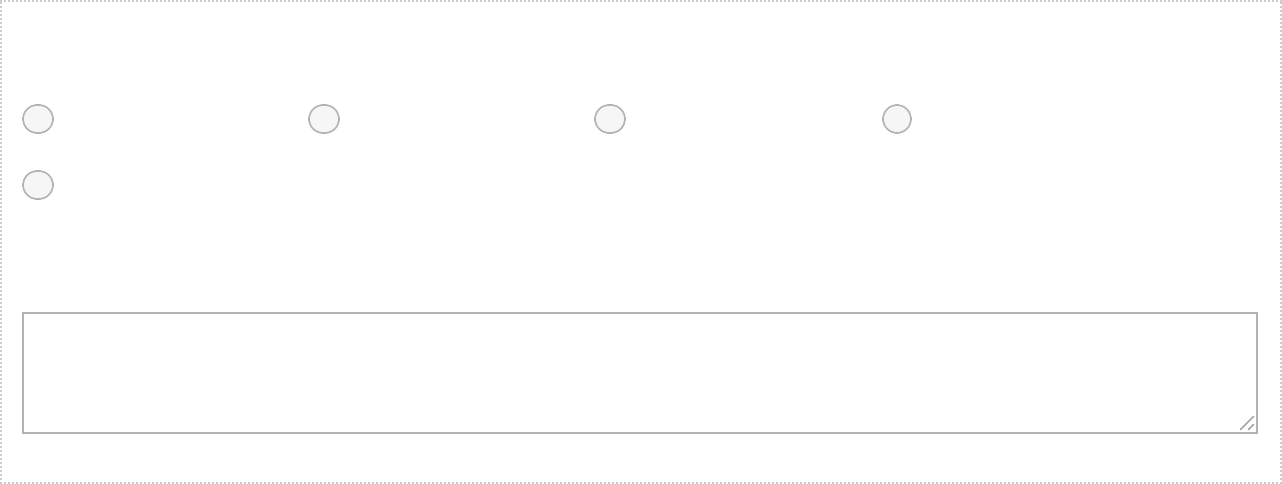


7. What is your estimated general level of literacy of technology at work?

Low

Somewhat low

Neither high nor low

Somewhat high

High

Comments

# 


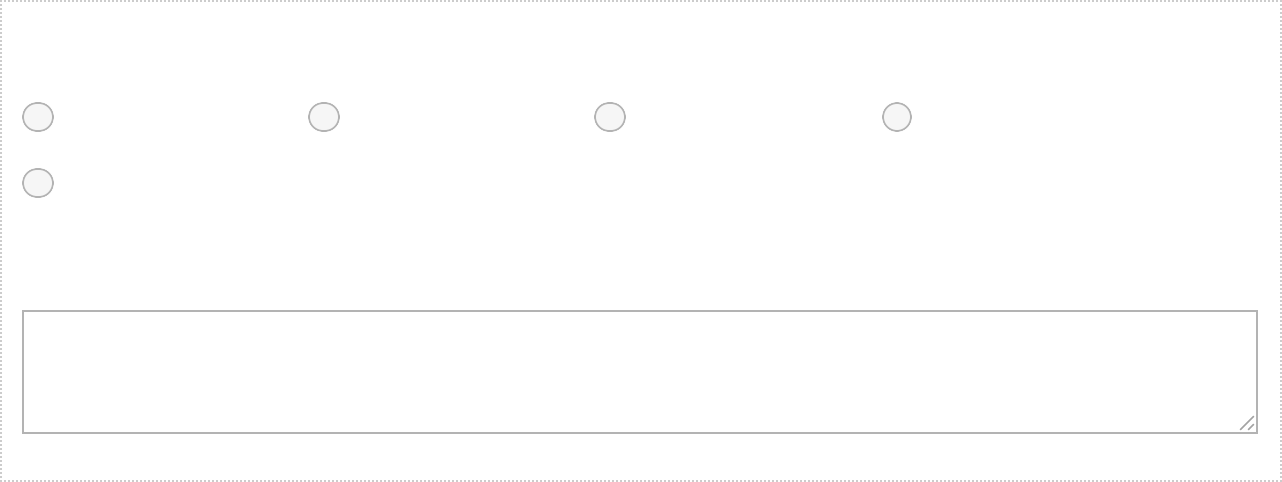


8. What is you estimated level of literacy of AI, artificial intelligence?

Low

Somewhat low

Neither high nor low

Somewhat high

High

Comments

# 


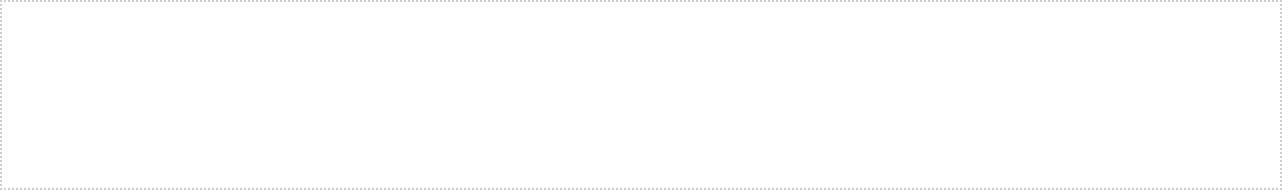


Attitudes towards artificial intelligence, AI, in the work

# 


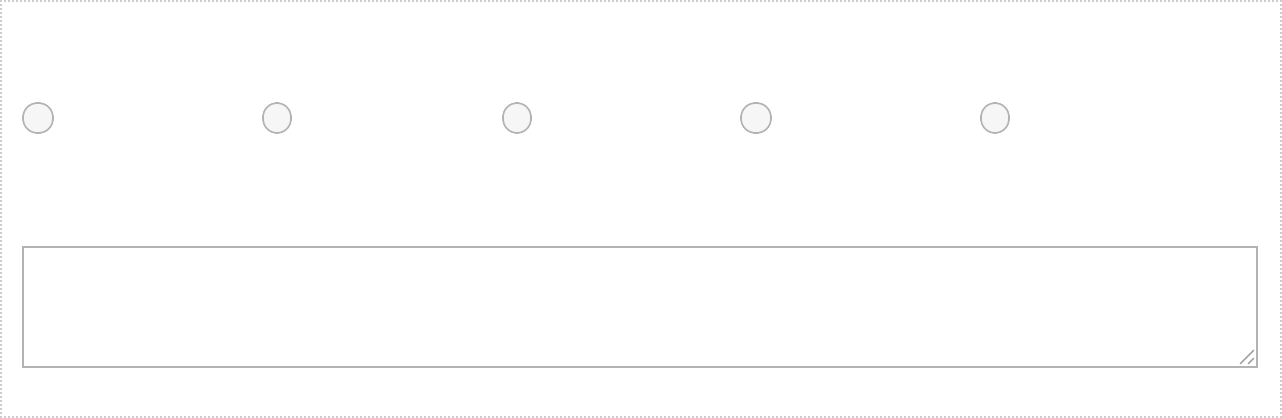


9. What is your attitude towards the use of AI-supported mammography screening?

Negative

Somewhat negative

Uncertain

Somewhat positive

Positive

Comments


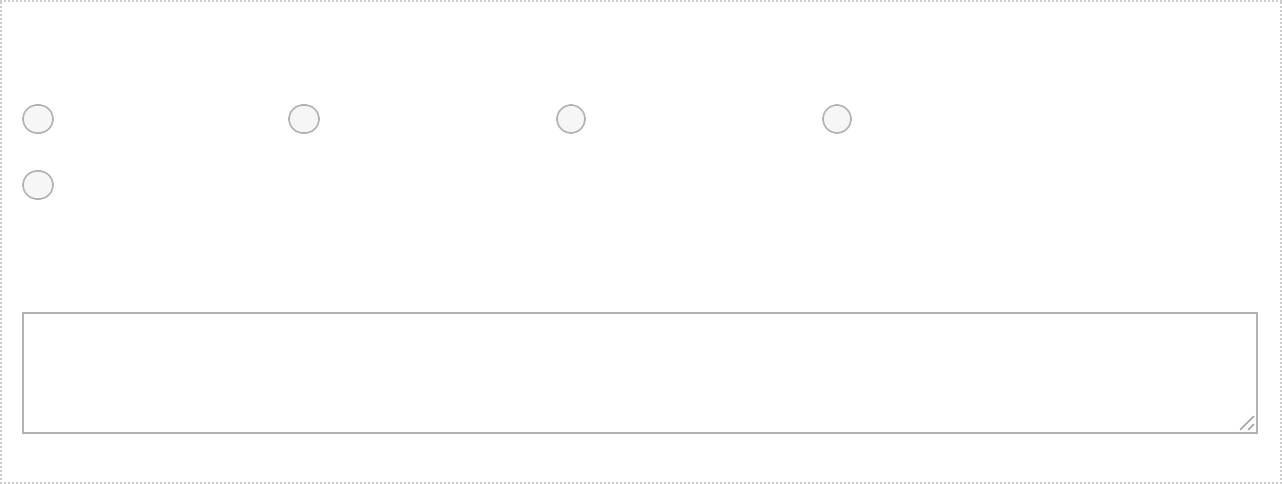


10. Do you consider that there are potential benefits of using AI-supported mammography screening?

To low degree

To somewhat low degree

Uncertain

To somewhat high degree

To high degree

Comments

# 


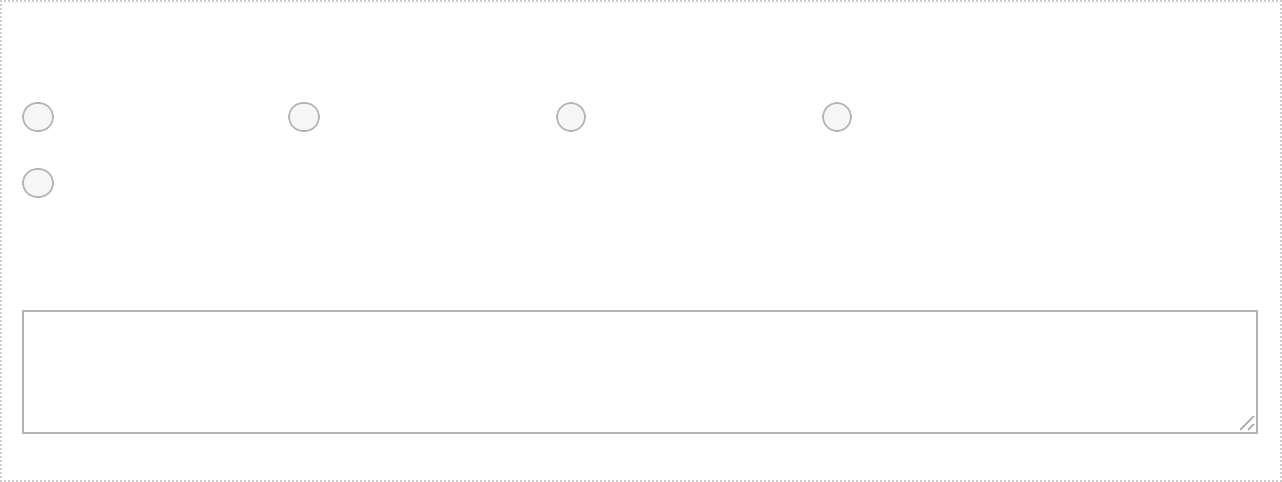


11. Do you consider that there are potential risks of using AI-supported mammography screening?

To low degree

To somewhat low degree

Uncertain

To somewhat high degree

To high degree

Comments

# 

# 


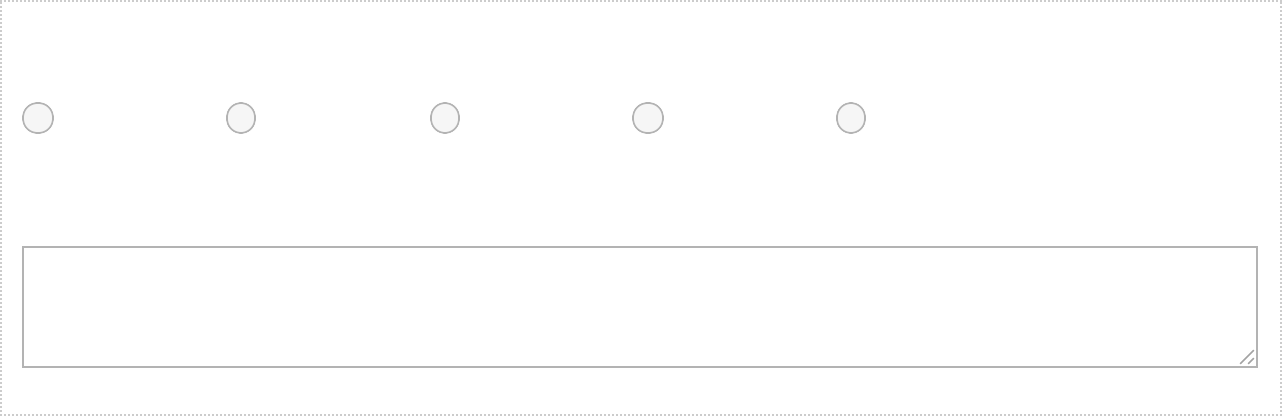


12. How much experience do you have of using AI in breast radiology?

None

Little

Somewhat little

Somewhat large

Large

Comments

# 


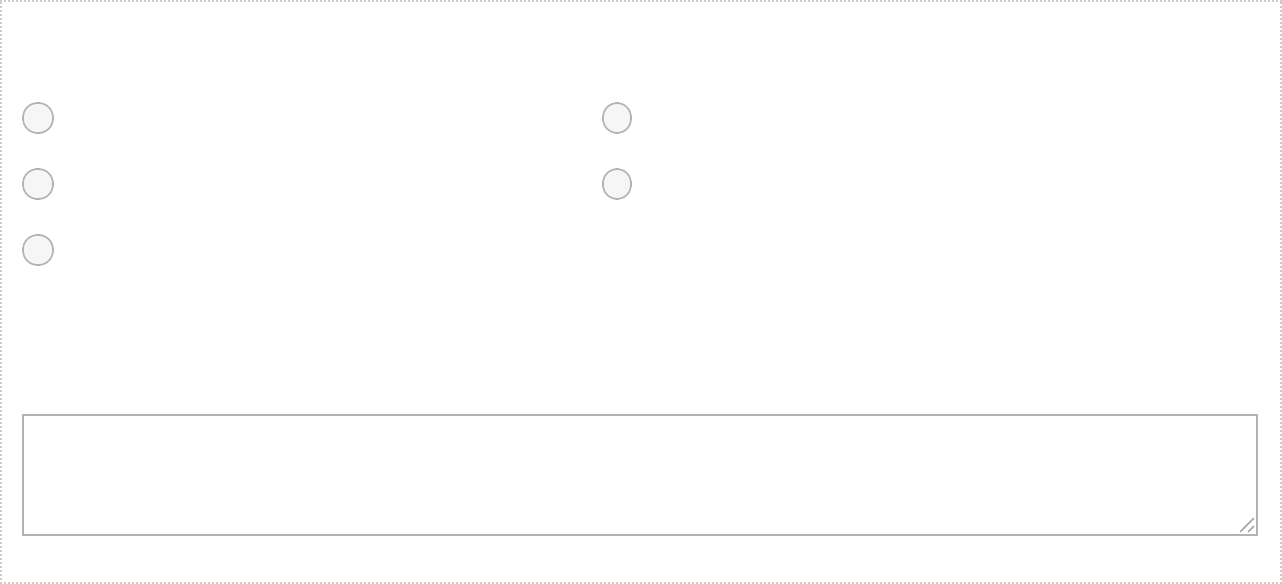


13. How would you like to use AI in mammography screening?

AI as triage tool

AI as stand-alone reader

AI as replacement of one radiologist in double screen-reading

AI as addition to double screen-reading

Not at all

There are several possibilities of how to use AI in screening, please, write your suggestion or comments to the options mentioned above, for example how you would like to combine different options.

# 


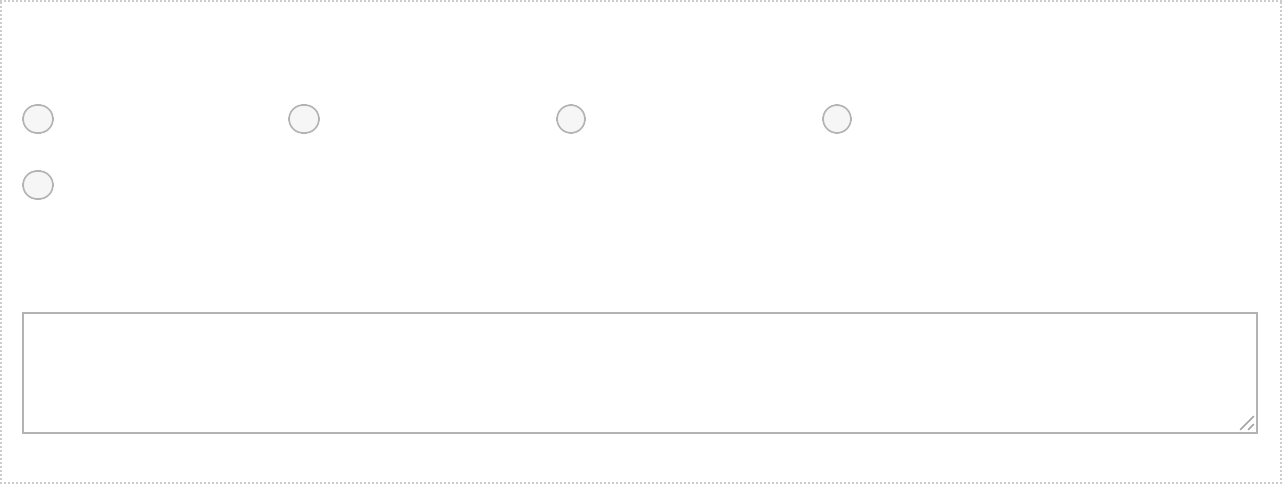


14. To what degree would you trust the assessments made by an AI-system?

To low degree

To somewhat low degree

Uncertain

To somewhat high degree

To high degree

Comments

# 


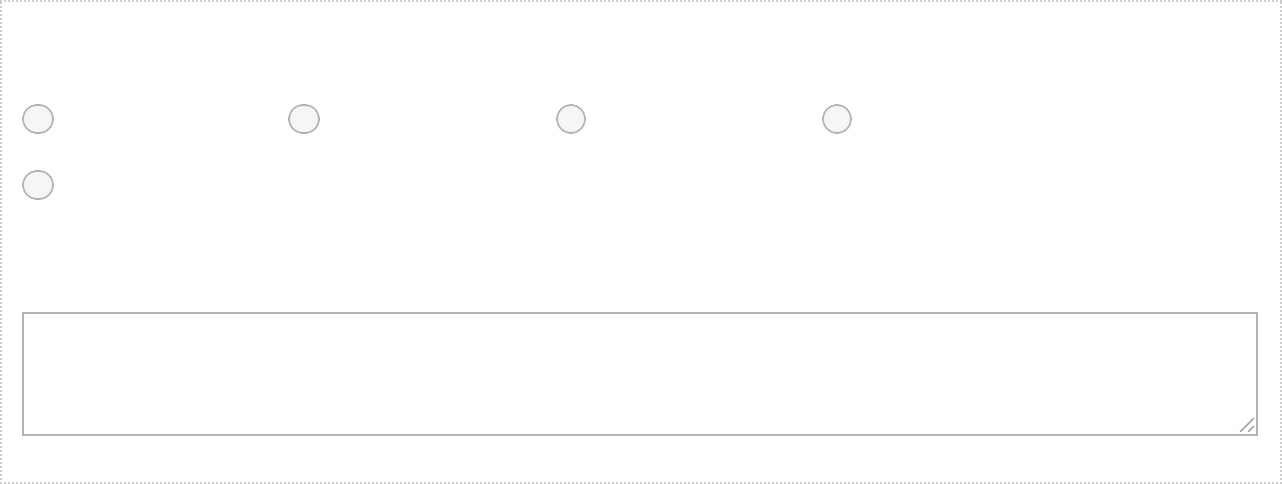


15. Do you think there is a risk of radiologists over-relying on assessments made by AI-systems?

To low degree

To somewhat low degree

Uncertain

To somewhat high degree

To high degree

Comments


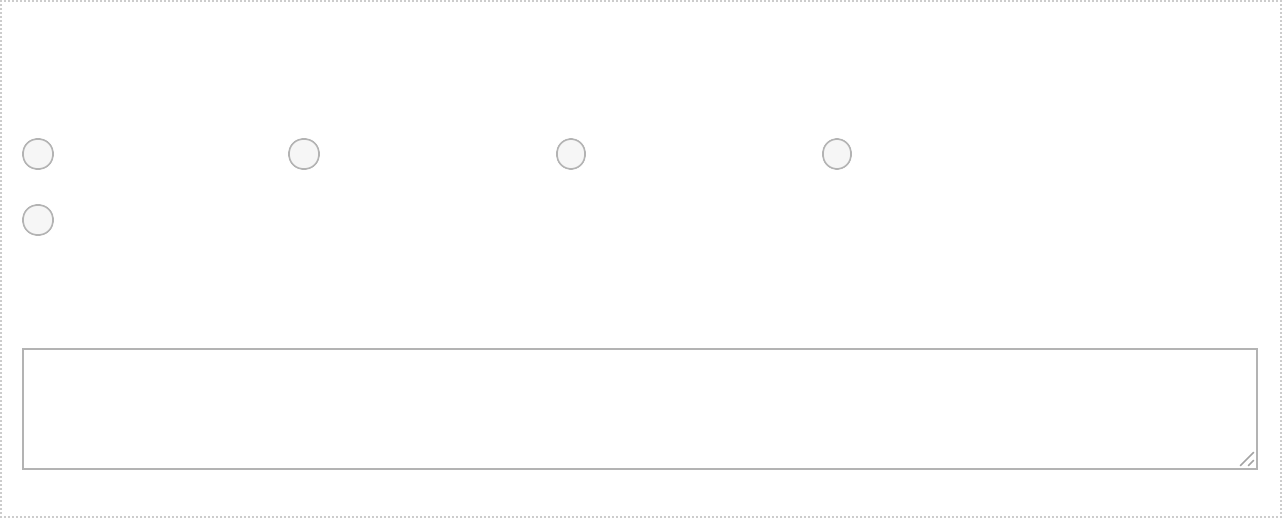


16. Would your trust in the AI-system be increased if your assessment contributed to train the AI-system after clinical implementation?

To low degree

To somewhat low degree

Uncertain

To somewhat high degree

To high degree

Comments


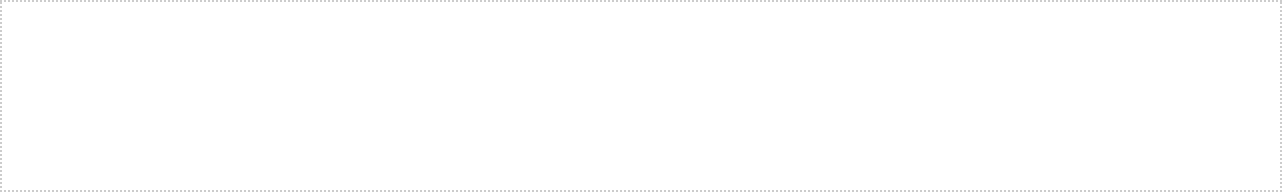


**Whom do you consider being responsible for assessments made by AI-supported mammography screening?**


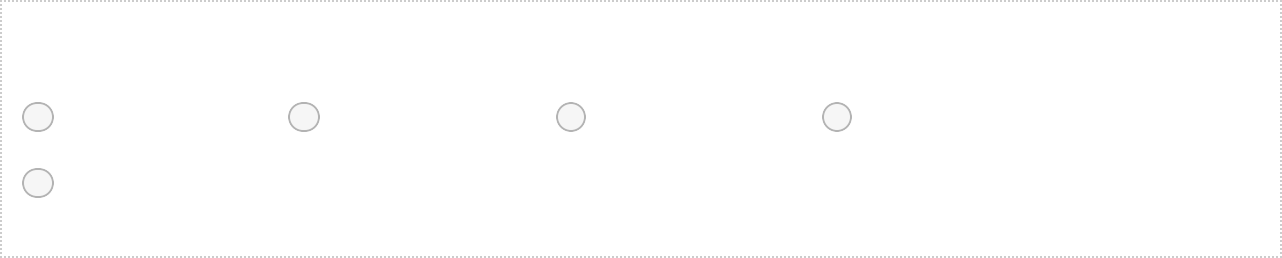


17. The radiologist (e.g., as co-reader)

To low degree

To somewhat low degree

Uncertain

To somewhat high degree

To high degree

# 


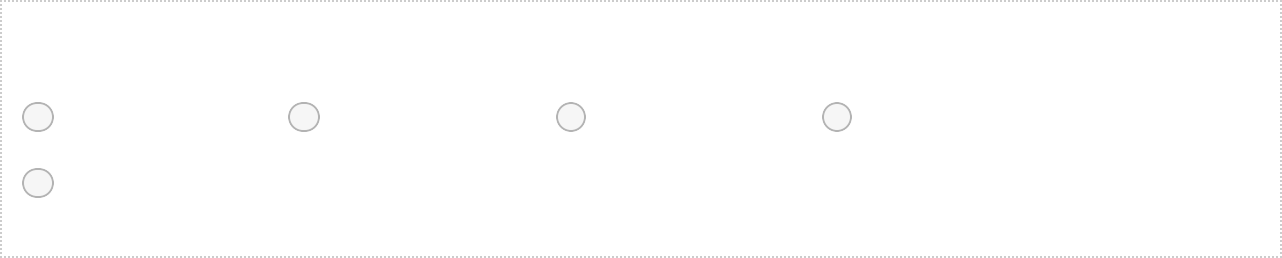


18. The health care provider

To low degree

To somewhat low degree

Uncertain

To somewhat high degree

To high degree

# 


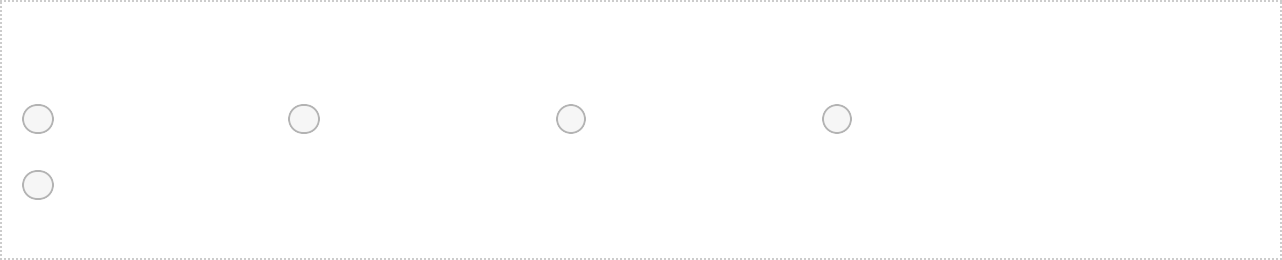


19. The AI-system

To low degree

To somewhat low degree

Uncertain

To somewhat high degree

To high degree

# 


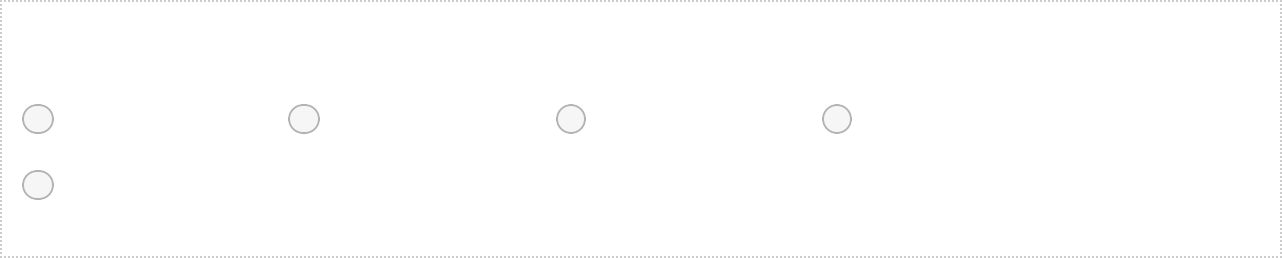


20. Developers of the AI-system

To low degree

To somewhat low degree

Uncertain

To somewhat high degree

To high degree

# 


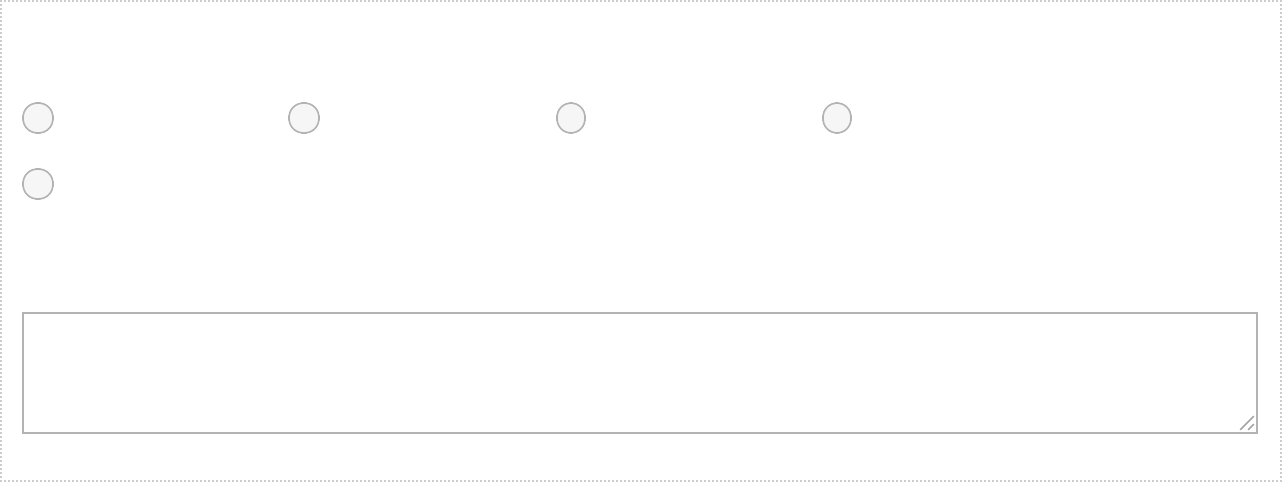


21. Shared responsibility

To low degree

To somewhat low degree

Uncertain

To somewhat high degree

To high degree

If you consider there to be shared responsibility, specify between which actors and why:

# 


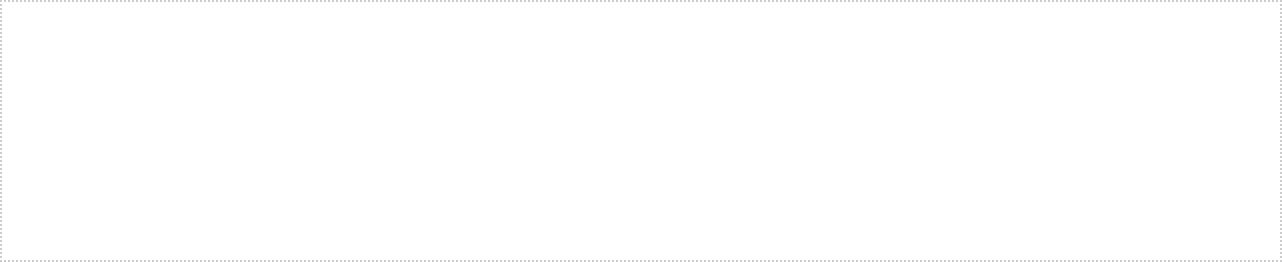


**Whom do you consider being responsible for assessments made by AI-supported mammography screening with AI as single reader?**


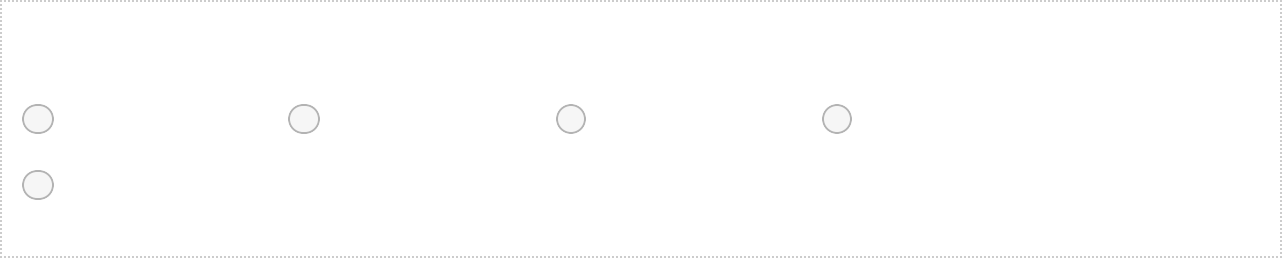


22. The radiologist (e.g., in terms of oversight)

To low degree

To somewhat low degree

Uncertain

To somewhat high degree

To high degree

# 


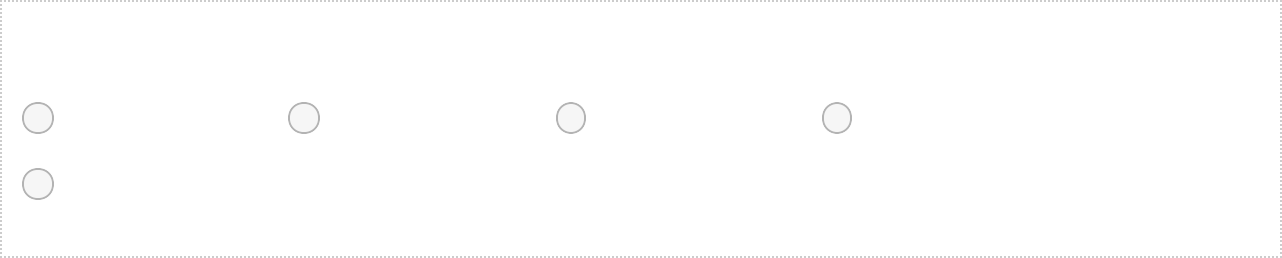


23. The health care provider

To low degree

To somewhat low degree

Uncertain

To somewhat high degree

To high degree

# 


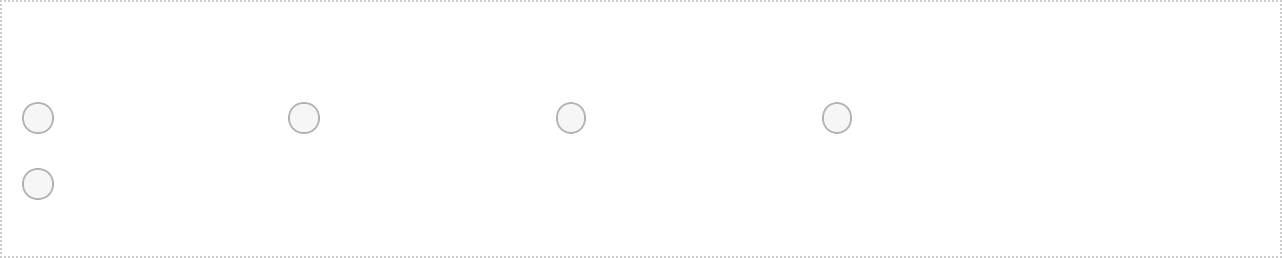


24. The AI-tool

To low degree

To somewhat low degree

Uncertain

To somewhat high degree

To high degree

# 


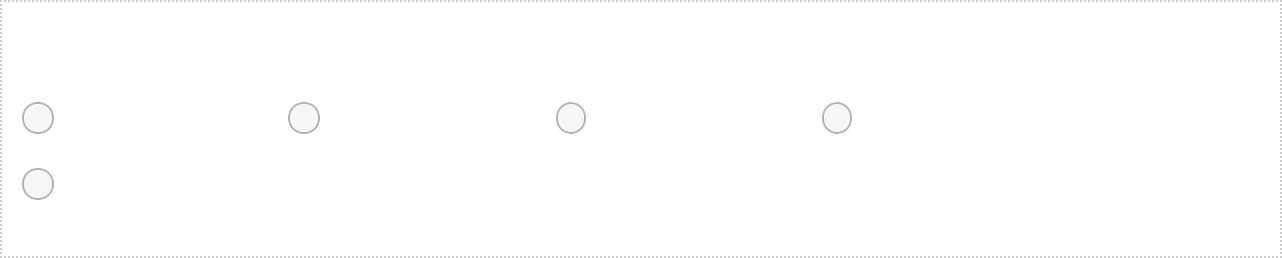


25. The developer of the AI-system

To low degree

To somewhat low degree

Uncertain

To somewhat high degree

To high degree

# 


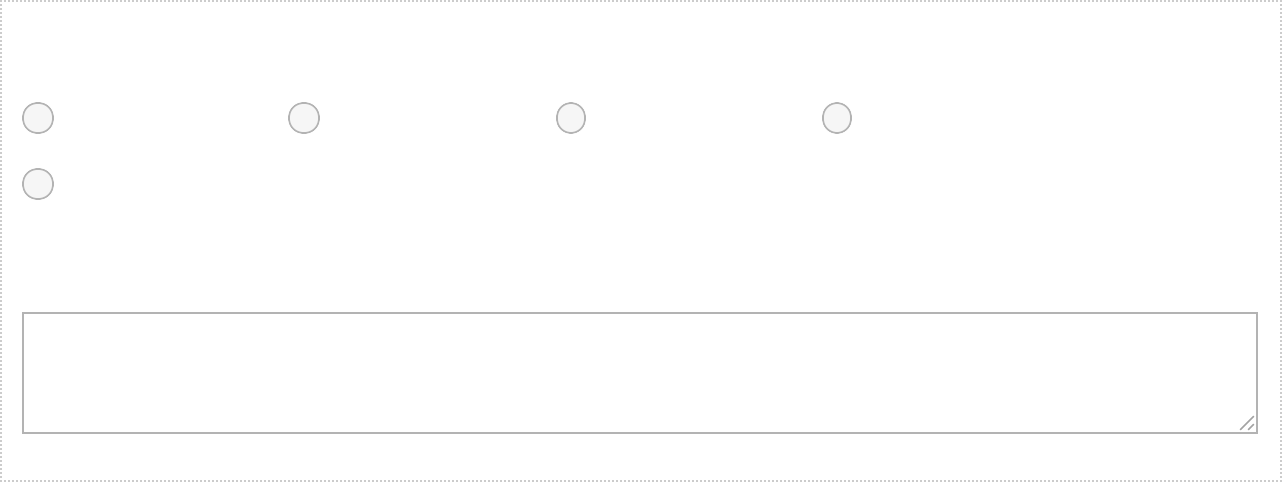


26. Shared responsibility

To low degree

To somewhat low degree

Uncertain

To somewhat high degree

To high degree

If you consider there to be shared responsibility, specify between which actors and why:

# 


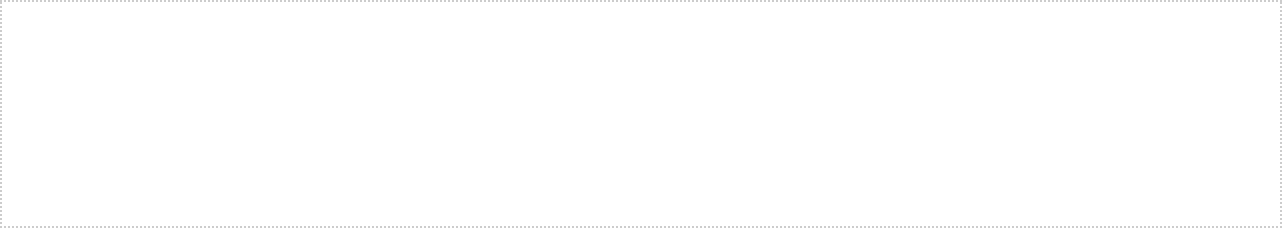


If you are using/have been using AI in your work, do you consider that you have recieved the information you need, to be able to evaluate the accuracy of the AI-system’s assessment:


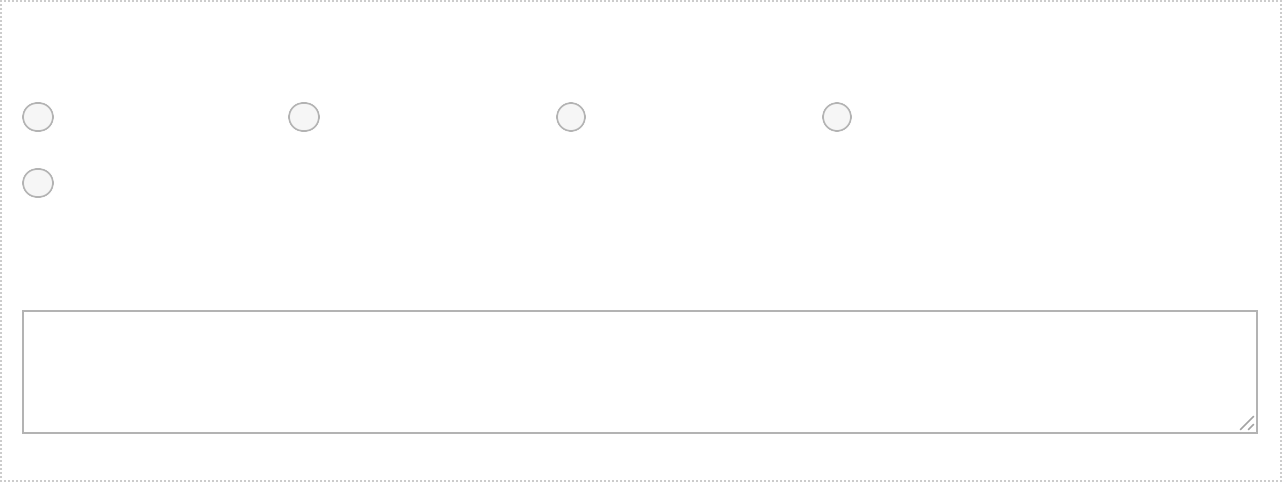


27. When using the system (information in user interface etc.)?

To low degree

To somewhat low degree

Uncertain

To somewhat high degree

To high degree

Comments


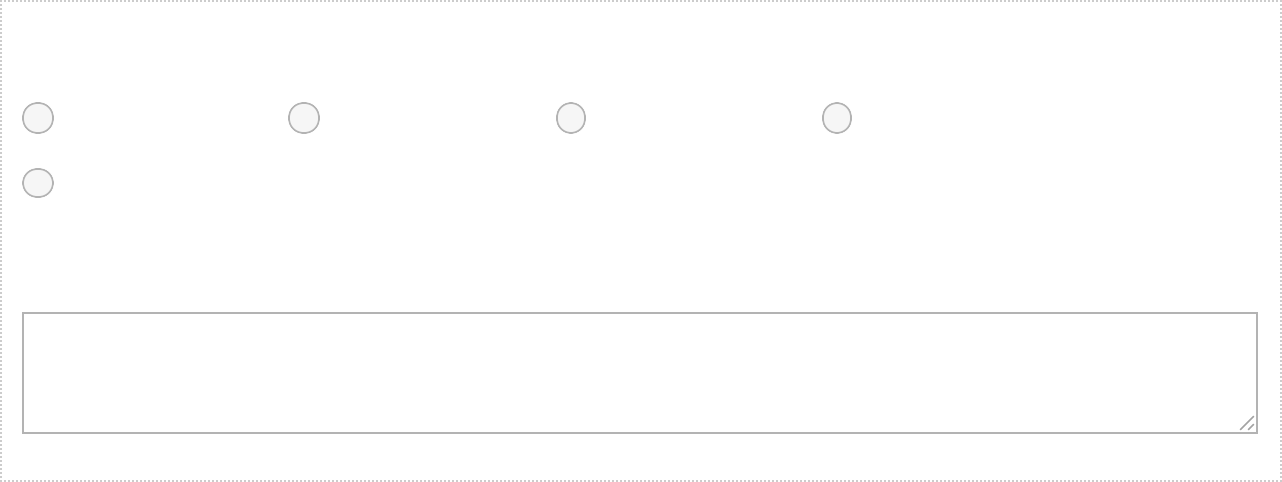


28. Prior to use of AI-system (information from developer etc.):

To low degree

To somewhat low degree

Uncertain

To somewhat high degree

To high degree

Comments


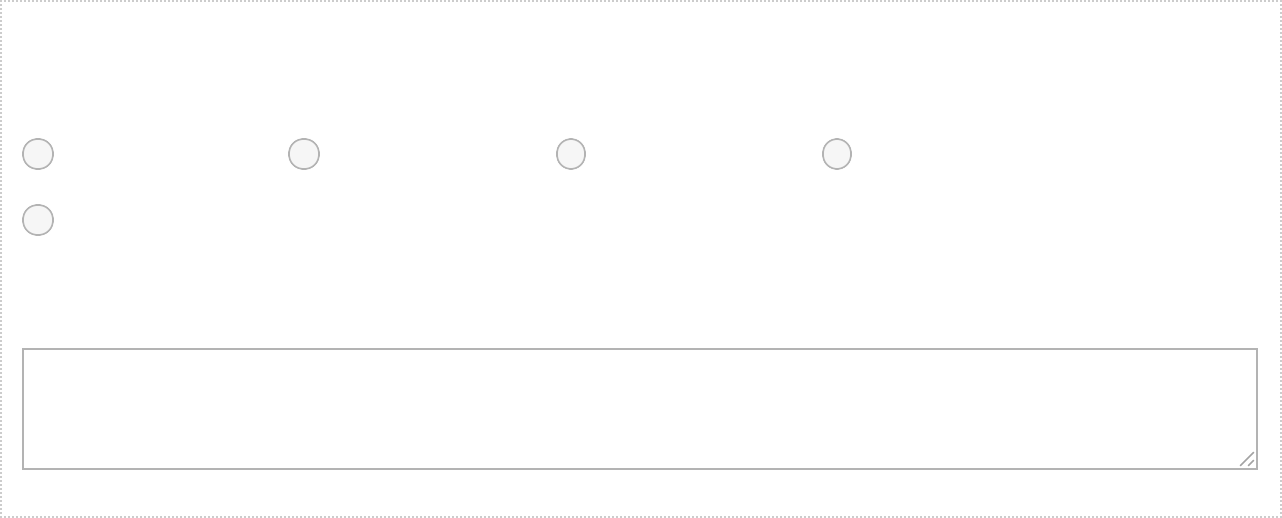


29. If you were to use an AI system in mammography screening, would you wish to receive information about how it was developed (for example, regarding what data were used for training)?

To low degree

To somewhat low degree

Uncertain

To somewhat high degree

To high degree

Comments

# 


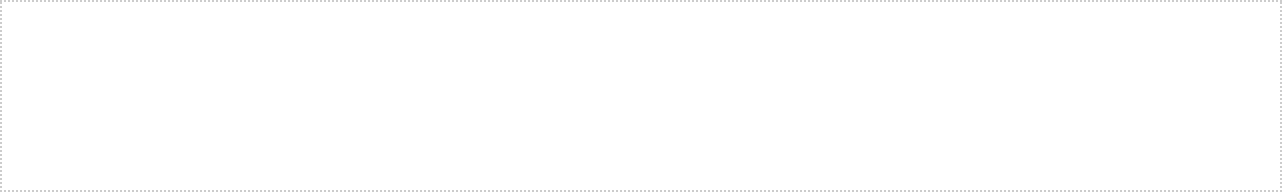


Do you think that the following information would have supported your trust evaluation of the assessments
made by an AI-system:


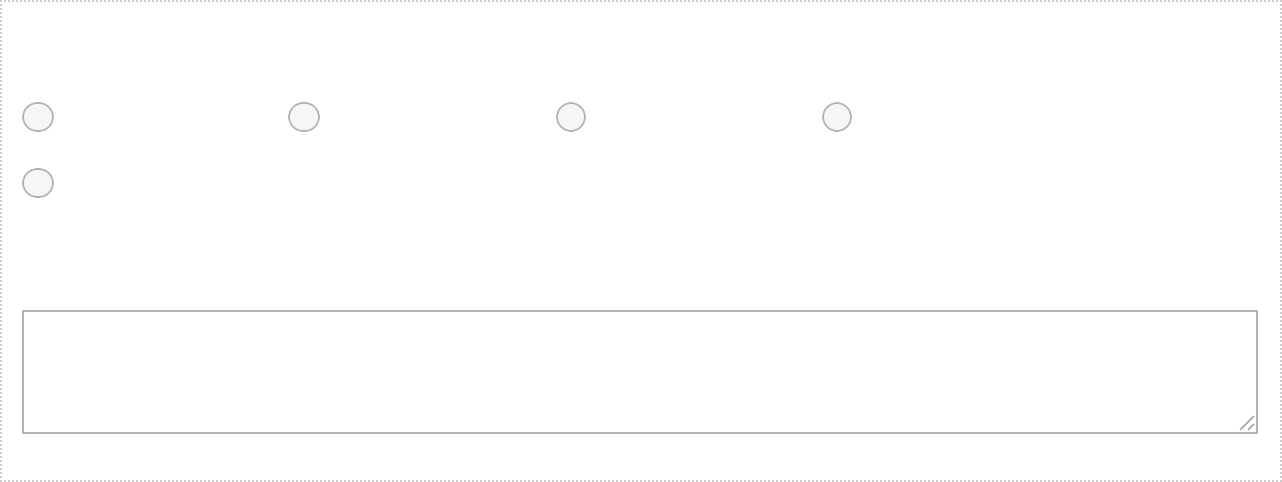


30. Information about what in the image that caused the given risk score?

To low degree

To somewhat low degree

Uncertain

To somewhat high degree

To high degree

Comments

# 


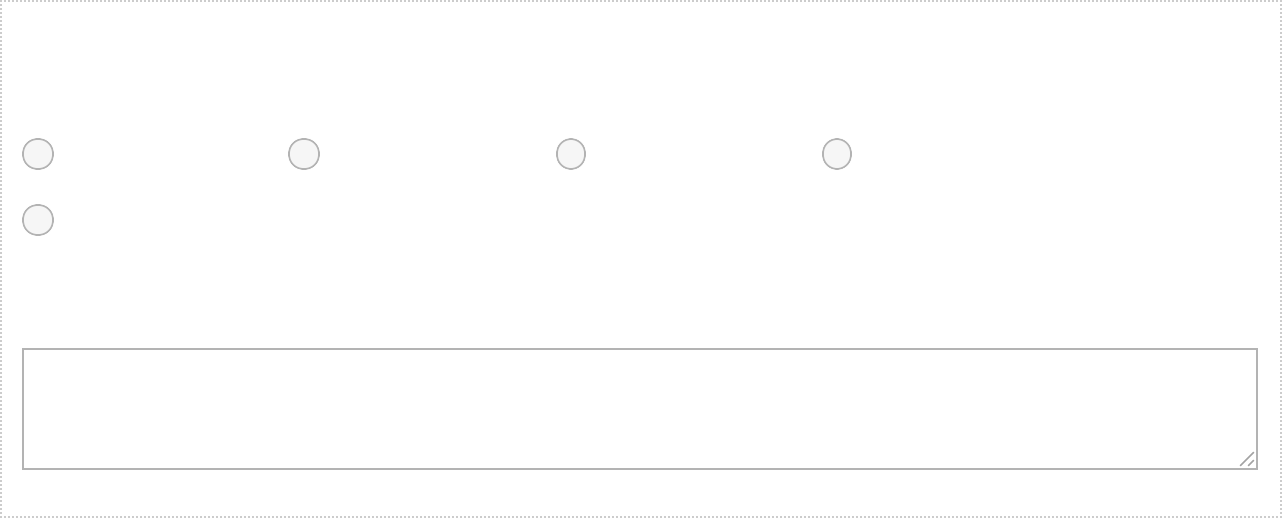


31. Information about what, and how much, in the image that would have had to be different for it to receive a lower versus higher risk score?

To low degree

To somewhat low degree

Uncertain

To somewhat high degree

To high degree

Comments

# 


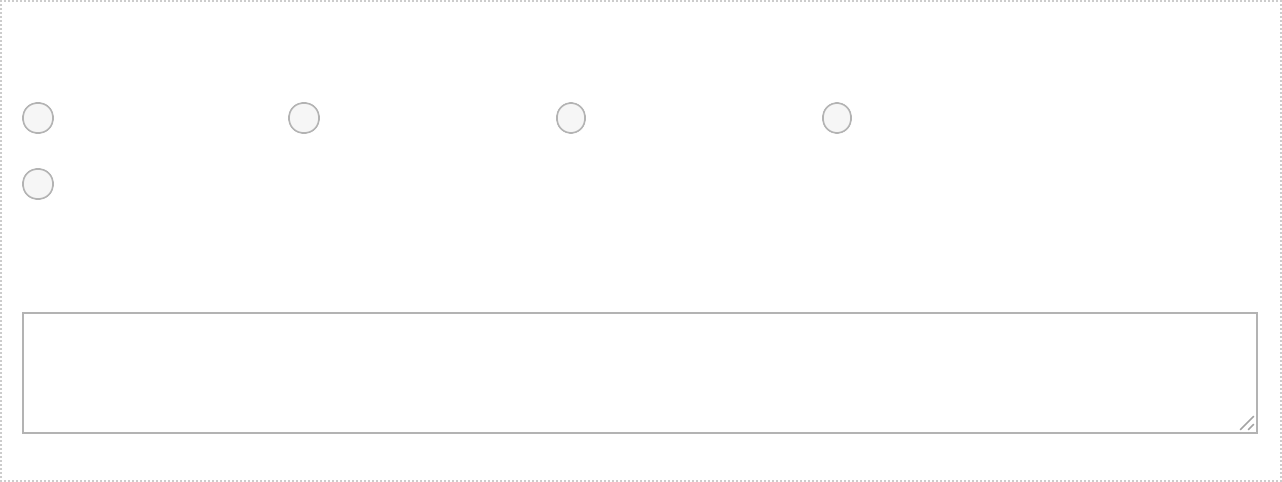


32. Information about the underlying code/algorithms?

To low degree

To somewhat low degree

Uncertain

To somewhat high degree

To high degree

Comments

# 


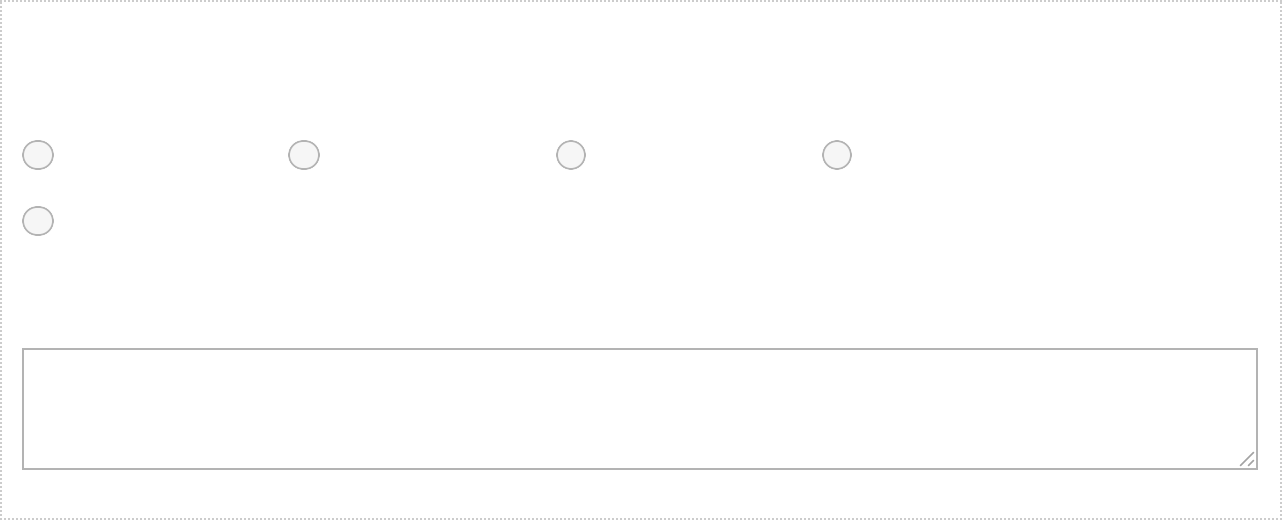


33. Information about competences involved in the development of the AI-system (e.g., breast radiologist, computer scientist, statisticians)?

To low degree

To somewhat low degree

Uncertain

To somewhat high degree

To high degree

Comments

# 


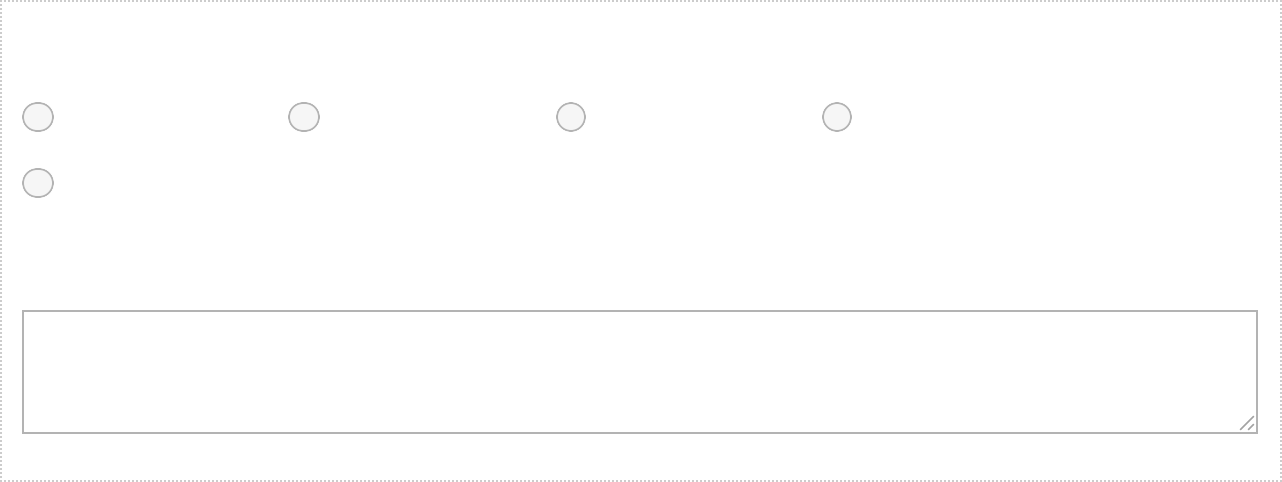


34. Information about training data?

To low degree

To somewhat low degree

Uncertain

To somewhat high degree

To high degree

Comments

# 


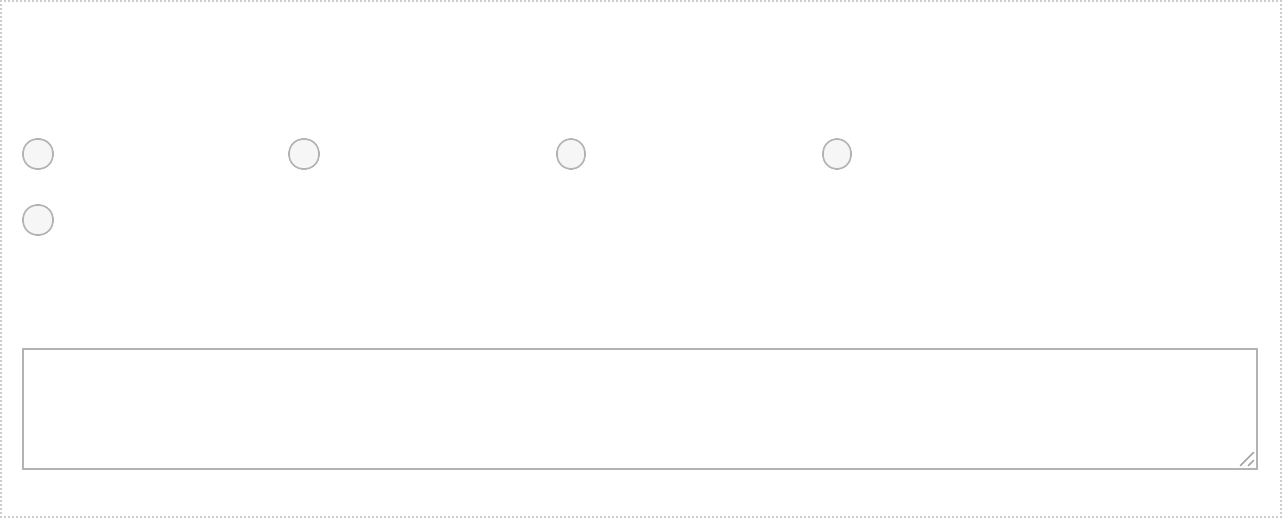


35. Information the labelling of the training data (e.g., how exams with cancer have been identified and annotated for the training of the AI-system)?

To low degree

To somewhat low degree

Uncertain

To somewhat high degree

To high degree

Comments

# 


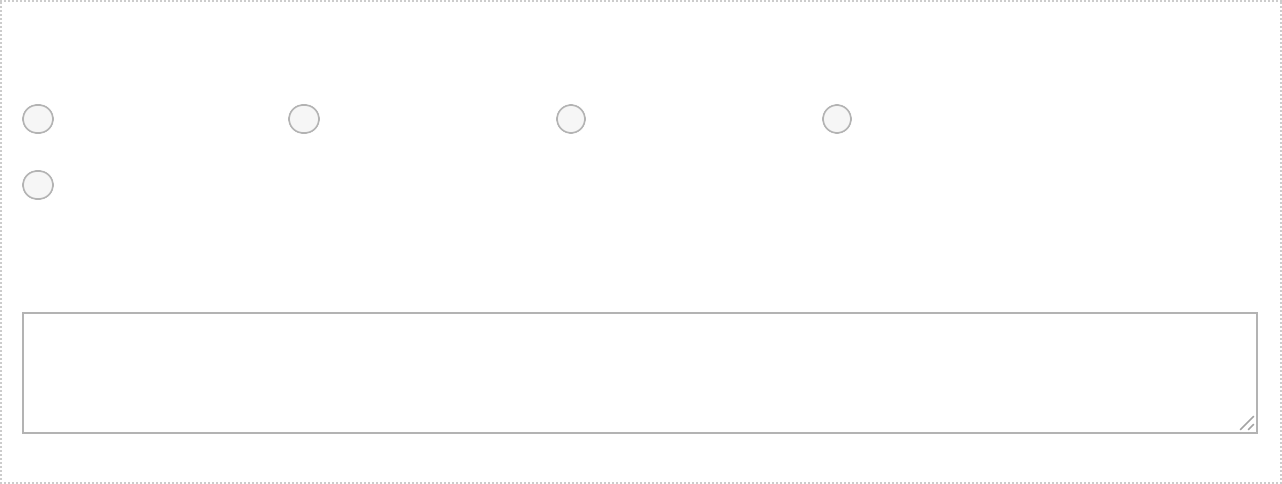


36. Information about continuous training of the AI-system after clinical implementation?

To low degree

To somewhat low degree

Uncertain

To somewhat high degree

To high degree

Comments

# 


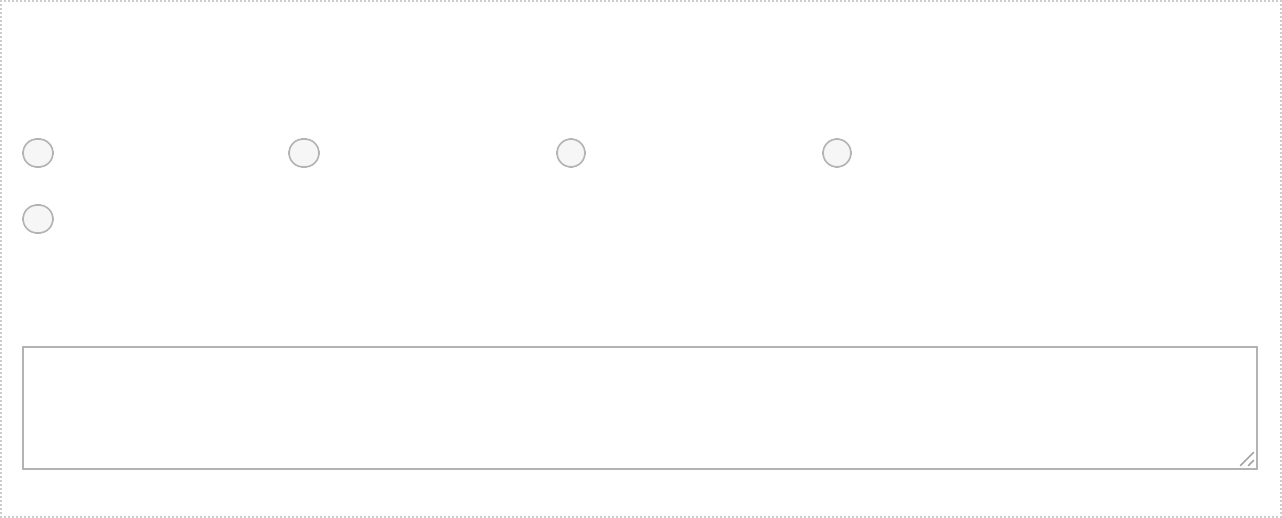


37. Do you consider that you would have competency enough to evaluate the accuracy of an AI-system’s assessment of screening exams?

To low degree

To somewhat low degree

Uncertain

To somewhat high degree

To high degree

Comments

# 


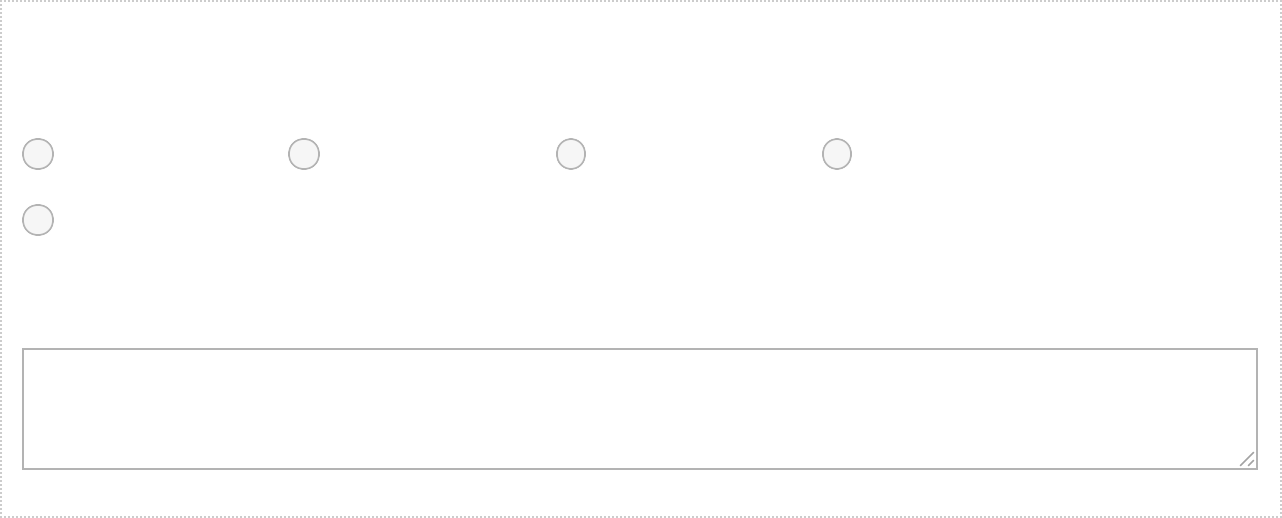


38. Do you think there is a risk of the training data for AI-system not being representative enough for the demographics of the target population?

To low degree

To somewhat low degree

Uncertain

To somewhat high degree

To high degree

Comments

# 


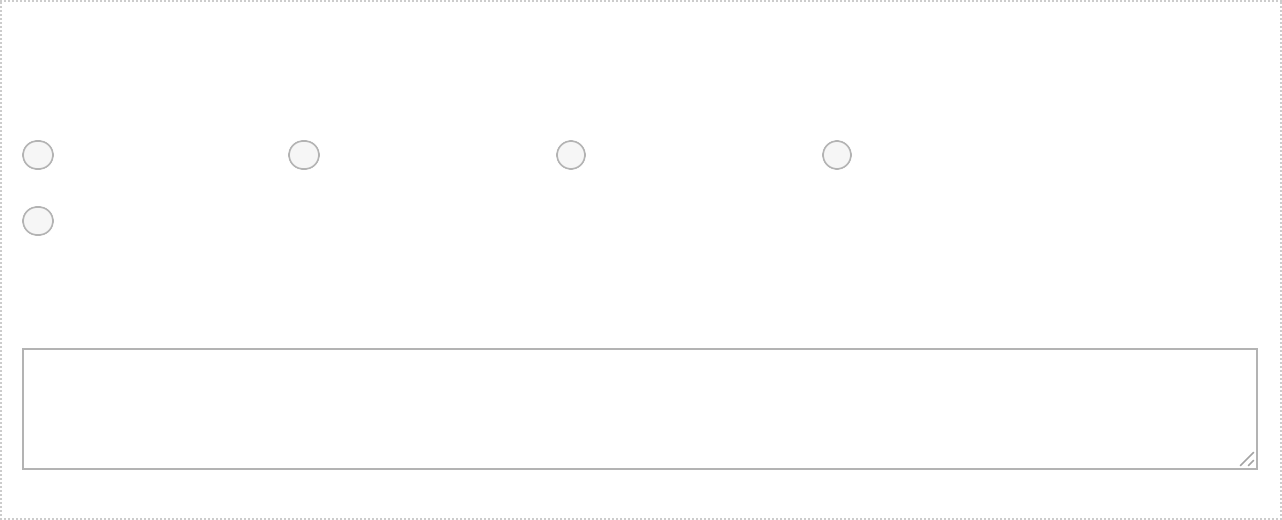


39. Do you think there is a risk that the performance of AI-systems is lower than radiologists’ regarding exams of certain risk groups or cases?

To low degree

To somewhat low degree

Uncertain

To somewhat high degree

To high degree

If so, describe which risk groups/cases:

# 


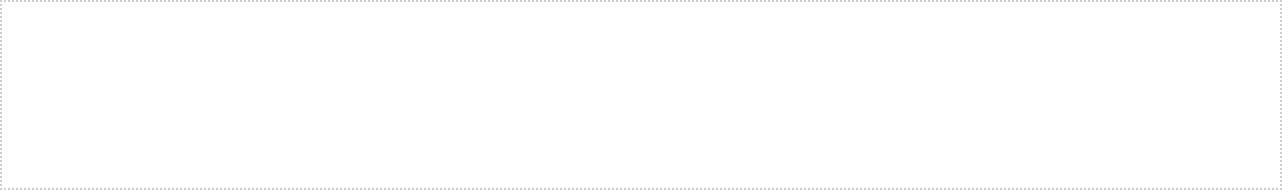


Development of the profession


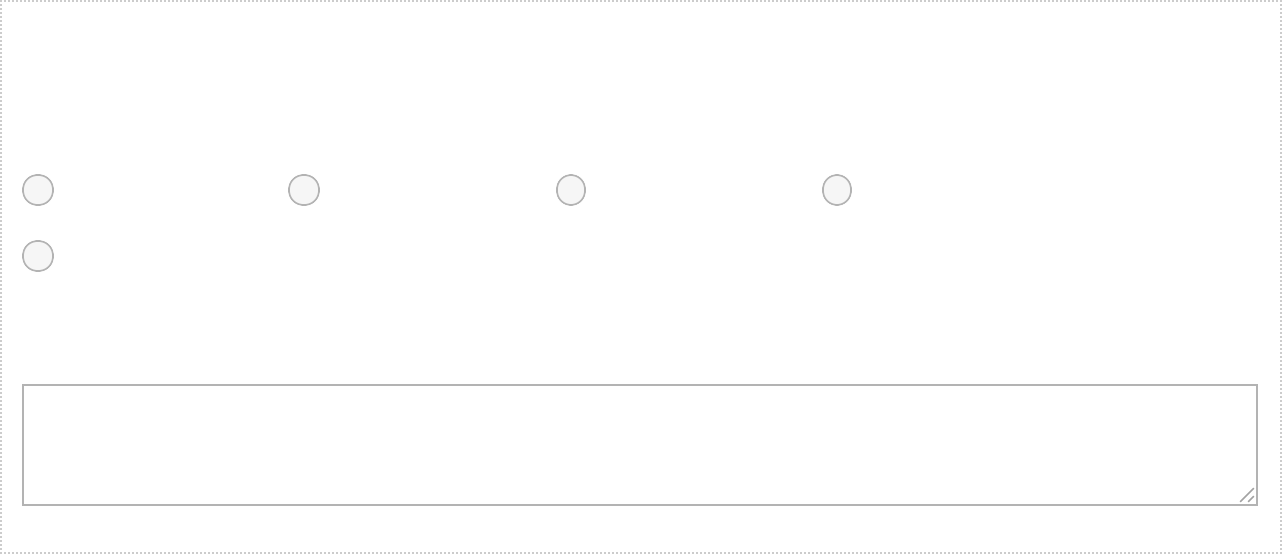


40. If you would be using AI in your work with mammography screening, would you wish for your evaluation of the AI assessment to be included in the continuous training of the system for improving its precision?

To low degree

To somewhat low degree

Uncertain

To somewhat high degree

To high degree

Comments

# 


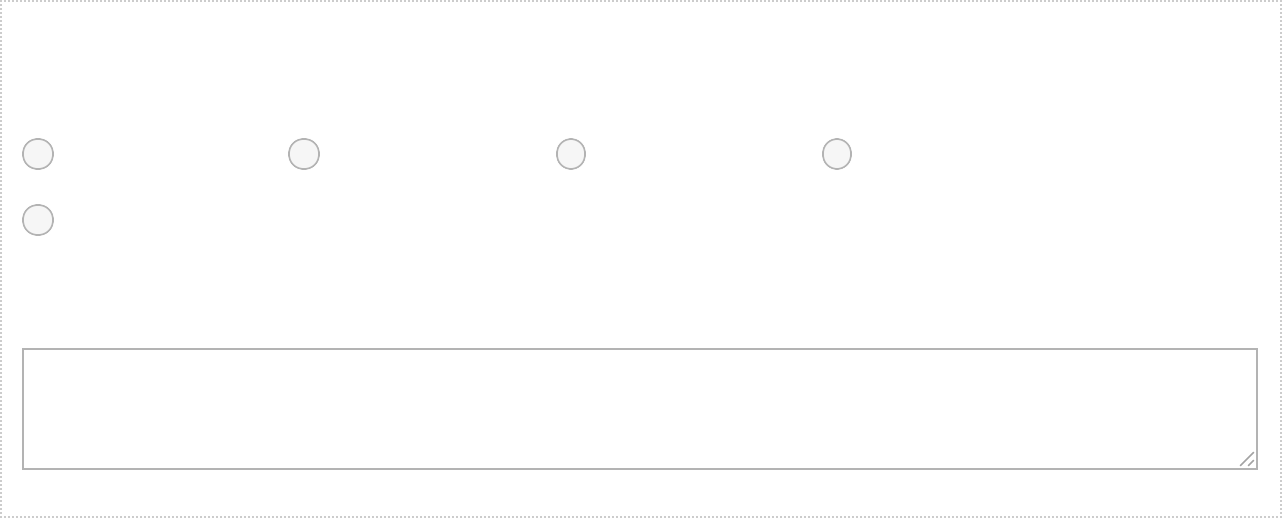


41. Do you consider there to be significant differences in implementing AI in mammography screening, in comparison to previous technologies (e.g., digital mammography, tomosynthesis)?

To low degree

To somewhat low degree

Uncertain

To somewhat high degree

To high degree

Comments

# 


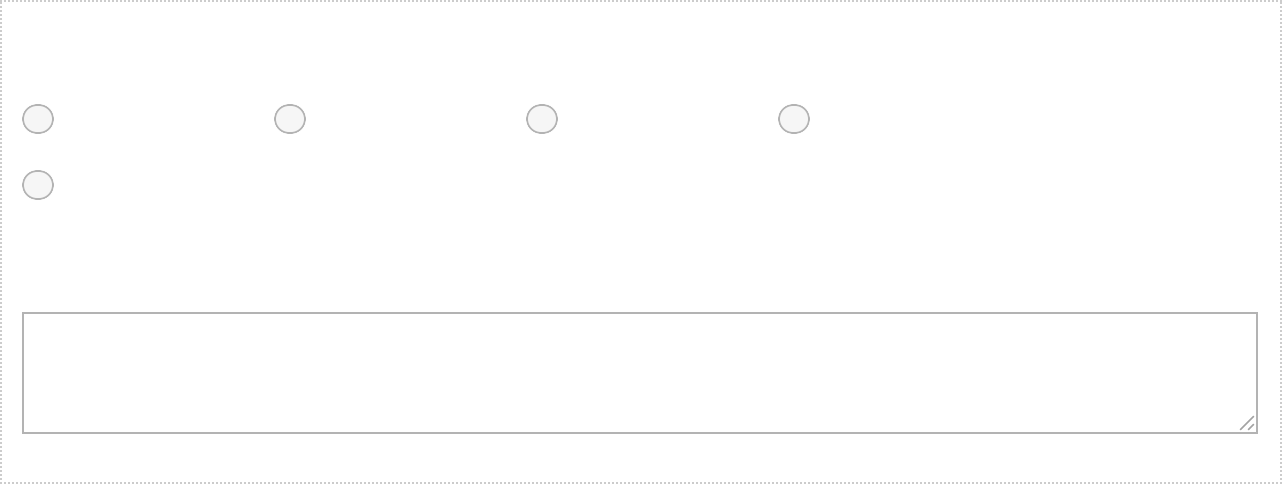


42. How do you think the role of the breast radiologist would change if AI-supported mammography screening was implemented?

Weakened

Somewhat weakened

Not at all

Somewhat strengthened

Strengthened

Comments

# 


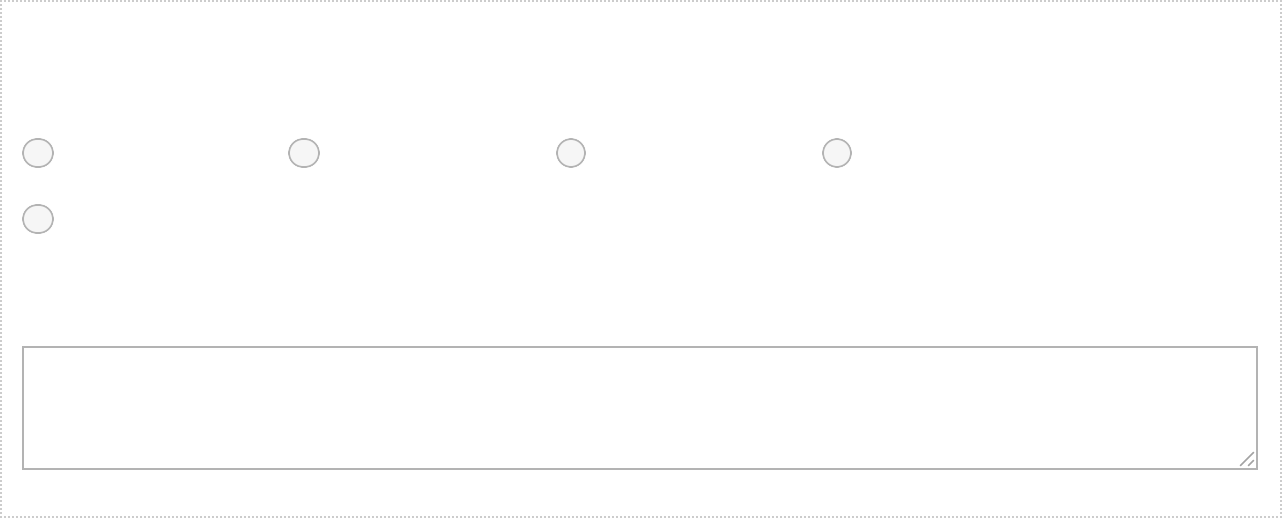


43. Do you think implementing AI-supported mammography screening would make recruitment of breast radiologists easier?

To low degree

To somewhat low degree

Uncertain

To somewhat high degree

To high degree

Comments

# 

44. Do you think that implementing AI-supported mammography screening would impact the relation between doctors/healthcare and screening participants? If so, how?

45. How do you think the profession will evolve, with regards to current technological development of AI?
